# Impact of Fluoxetine Treatment and Folic Acid Supplementation on the Mammary Gland Transcriptome During Peak Lactation

**DOI:** 10.3389/fphar.2022.828735

**Published:** 2022-02-23

**Authors:** Celeste M. Sheftel, Lihe Liu, Sena L. Field, Samantha R. Weaver, Chad M. Vezina, Francisco Peñagaricano, Laura L. Hernandez

**Affiliations:** ^1^ Molecular and Cellular Pharmacology Training Program, University of Wisconsin-Madison, Madison, WI, United States; ^2^ Department of Animal and Dairy Sciences, University of Wisconsin-Madison, Madison, WI, United States; ^3^ Department of Orthopedic Surgery, Mayo Clinic, Rochester, MN, United States; ^4^ Department of Comparative Biosciences, University of Wisconsin-Madison, Madison, WI, United States

**Keywords:** calcium signaling, serotonin, SSRI, folic acid, lactation, involution, lipid metabolism

## Abstract

Serotonin is a key regulator of mammary gland homeostasis during lactation. Selective serotonin reuptake inhibitors (SSRIs) are commonly used to treat peripartum depression, but also modulates mammary gland serotonin concentrations and signaling in part through DNA methylation. The objective of this study was to determine mouse mammary transcriptome changes in response to the SSRI fluoxetine and how methyl donor supplementation, achieved by folic acid supplementation, affected the transcriptome. Female C57BL/6J mice were fed either breeder diet (containing 4 mg/kg folic acid) or supplemented diet (containing 24 mg/kg folic acid) beginning 2 weeks prior to mating, then on embryonic day 13 mice were injected daily with either saline or 20 mg/kg fluoxetine. Mammary glands were harvested at peak lactation, lactation day 10, for transcriptomic analysis. Fluoxetine but not folic acid altered circulating serotonin and calcium concentrations, and folic acid reduced mammary serotonin concentrations, however only fluoxetine altered genes in the mammary transcriptome. Fluoxetine treatment altered fifty-six genes. *Elovl6* was the most significantly altered gene by fluoxetine treatment along with gene pathways involving *fatty acid homeostasis*, *PPARγ,* and *adipogenesis*, which are critical for milk fat synthesis. Enriched pathways in the mammary gland by fluoxetine revealed pathways including *calcium signaling, serotonin receptors, milk proteins*, and *cellular response to cytokine stimulus* which are important for lactation. Although folic acid did not impact specific genes, a less stringent pathway analysis revealed more diffuse effects where folic acid enriched pathways involving *negative regulation of gene expression* as expected, but additionally enriched pathways involving *serotonin*, *glycolysis*, and *lactalbumin* which are critical for lactation. In conclusion, peripartal SSRI use and folic acid supplementation altered critical genes related to milk synthesis and mammary gland function that are important to a successful lactation. However, folic acid supplementation did not reverse changes in the mammary gland transcriptome altered by peripartal SSRI treatment.

## 1 Introduction

Serotonin (5-hydroxytryptamine, 5HT) is a monoamine that acts as an important neurotransmitter for mood and behavior in the brain. However, non-neuronal 5HT accounts for the greatest amount (95%) of total body 5HT levels where it acts primarily as a hormone. Non-neuronal 5HT is involved in numerous biological functions and is particularly important in the mammary gland as a regulator of lactation, where the mammary gland contributes approximately 50% of the circulating non-neuronal 5HT content (Weaver et al., 2017). Mammary-derived 5HT can act locally in an autocrine/paracrine manner to regulate involution through changes in mammary tight junction permeability (Marshall et al., 2010; Pai et al., 2015), milk protein synthesis (Matsuda et al., 2004), and calcium homeostasis (Hernandez et al., 2012a; Laporta et al., 2014a). Additionally, mammary-derived 5HT participates in an integral systemic breast-to-bone communication during lactation by upregulating parathyroid hormone related protein (PTHrP), which liberates calcium from skeletal stores to maintain maternal calcium homeostasis (Hernandez et al., 2012a). 5HT regulates PTHrP in the mammary epithelial cells by multiple mechanisms: 5HT receptor 2B activation (Hernandez et al., 2012a), epigenetic activation of sonic hedgehog (Laporta et al., 2014b), and a transglutaminase-dependent serotonylation (Sheftel and Hernandez, 2020). Thus, the mammary gland maintains maternal calcium homeostasis by coordinating multiple systemic calcium signaling pathways to ensure sufficient calcium in milk for the nursing neonate.

Tryptophan hydroxylase 1 (TPH1) is the rate limiting enzyme in 5HT synthesis in the periphery, converting L-tryptophan to 5-hydroxytryptamine (5HTP), which is decarboxylated to create 5HT (Boadle-Biber, 1993). *Tph1*-knockout (KO) mice, which are deficient in non-neuronal 5HT, have a decreased serum calcium concentration, as well as altered mammary epithelial intracellular calcium transport through disrupted calcium transporter localization and decreased gene expression of the transporters (Laporta et al., 2014a). The *Tph1*-KO mouse mammary transcriptome exhibited alterations in calcium signaling, particularly calcium release from the endoplasmic reticulum, during lactation. Interrogation of the *Tph1*-KO lactating mammary transcriptome linked 5HT to biological pathways associated with lipid metabolism, fat cell differentiation, and insulin resistance (Laporta et al., 2015).

5HT signaling can be probed using various methods, including genetic *Tph1* ablation, 5HTP supplementation, and selective serotonin reuptake inhibitor (SSRI) treatment. SSRI antidepressants enhance the serotonergic system by increasing exposure of the neuron, or peripheral tissue, to 5HT through inhibition of the 5HT reuptake transporter (SERT). Additionally, SSRIs upregulate 5HT synthesis by increased TPH expression, concurrent with reduced 5HT degradation (Marshall et al., 2014). We previously reported that the SSRI fluoxetine increases mammary 5HT concentration and exacerbates lactation-associated bone loss, potentially by increasing PTHrP in the mammary gland (Weaver et al., 2018). Clinical depression is common during or after pregnancy, and SSRIs are the first-choice treatment for maternal depressive symptoms (Gavin et al., 2005; Koren and Nordeng, 2012). As such, exacerbated 5HT-PTHrP signaling may negatively impact the skeleton long-term for women taking SSRIs to treat maternal depression during the peripartum period. Further, given the widespread physiological roles of 5HT, it is possible that SSRI use during lactation may have numerous impacts on the mammary gland during lactation that may be critical for milk synthesis.

We have previously demonstrated that non-neuronal 5HT regulates PTHrP by decreasing DNA methylation of PTHrP and differential methylation of the sonic hedgehog DNA promoters (Laporta et al., 2014b; Weaver et al., 2018). In this study, we investigated transcriptomic changes in the mammary gland of lactating mice in response to peripartum fluoxetine treatment with or without the well-known methyl donor folic acid (FA) (Anderson et al., 2012) as a potential translatable rescue for these altered methylation states.

## 2 Materials and Methods

### 2.1 Animal Handling and Experimental Design

Mammary gland tissues used in this experiment were collected from a previous study (Weaver et al., 2018). All experiments were performed under protocol #V01426, approved by the Research Animal Care and Use Committee at the University of Wisconsin-Madison. C57BL/6J mice were maintained at a temperature of 25°C, 20%–50% humidity, with a 12-hour light/dark cycle with *ad libitum* access to food and water. Two weeks before mating, dams were randomly assigned into breeder diet (Harlan Diet 2019, Harlan Teklad, Madison, WI containing 4 mg/kg diet FA, *n* = 16) or the same base diet supplemented with FA (FA-enriched diet, Harlan Diet 120256 containing a final concentration of 24 mg/kg diet FA, *n* = 16). This diet formulation was previously used to induce methylation changes in our collaborator’s lab (Keil et al., 2015). In humans, the FA RDA increases from 400 µg to 600 µg per day during pregnancy, a 50% increase and then decreasing to 450 µg per day during lactation (Committee IoMUS, 1998). In our case we used a 100% increase from 2 mg/kg diet in AIN-93 (Reeves et al., 1993) to our 4 mg/kg diet to support breeding, pregnancy, and lactation and the 24 mg/kg diet FA is 10 times the daily requirement. The mice remained on their respective diet, either breeder diet or FA supplemented diet throughout the experimental period. Beginning at 6 weeks of age, female mice were bred overnight, and pregnancy was determined by observing a seminal plug, at which time the mice were housed individually and randomly assigned to fluoxetine or vehicle treatment. This resulted in 4 treatments: breeder diet + saline (*n* = 8), breeder diet + fluoxetine (*n* = 8), FA supplemented diet + saline (*n* = 8), or FA supplemented diet + fluoxetine (*n* = 7). Beginning on embryonic day 13 (E13) of pregnancy through day 10 of lactation (L10), dams received a daily intraperitoneal injection of either 20 mg/kg bodyweight fluoxetine hydrochloride (#F312, Sigma-Aldrich, St. Louis, MO, United States) in saline or sterile saline vehicle.

### 2.2 Sample Collection, Assays, and Analysis

Blood was collected at E13, L1, and L10 from the submandibular vein using a 5.5 mm lancet. Blood was then placed on ice for 20 min to allow for the disruption of platelets and centrifuged at 1,500 ×*g* at 4°C for 20 min to isolate serum, which was stored at −80°C until analysis. At L10, the dams were euthanized via carbon dioxide inhalation, followed by cervical dislocation. The mammary glands were rapidly extracted and frozen in liquid nitrogen to preserve tissue integrity, then stored at −80°C until RNA extractions.

Serotonin concentrations were determined using a 5HT Enzyme Immunoassay Kit (Beckman Coulter, #IM1749, Brea, CA, United States) using serum diluted 1:100 or 125 μg protein in radioimmunoprecipitation lysis buffer as previously described (Weaver et al., 2018). Total calcium was determined using a Calcium Assay Kit (Cayman Chemical Company, #701220, Ann Arbor, MI, United States) in serum diluted 1:2 according to the manufacturer’s instructions. 5HT assay had an intra-assay CV of <10% and calcium <5%.

Circulating 5HT, mammary gland 5HT concentration, and circulating calcium analyses were conducted using GraphPad Prism 9 (Version 9.2.0) or SAS (Version 9.4). Analysis between treatments without the effect of time were performed using a one-way ANOVA. Analyses with multiple time points were conducted using a mixed-model ANOVA with Tukey’s post-hoc pairwise comparisons test to detect differences between treatment groups. For all analyses, differences among means were considered significant if *p* < .05 or a tendency if .05 < *p* < .1. All values are reported as mean ± SEM.

### 2.3 RNA-Seq: RNA Extraction, Library Generation, Sequencing, Quality Control, and Read Mapping

Total RNA was extracted from the mammary gland using Qiagen RNeasy Micro Kit (#74004, Qiagen Germantown, MD, United States) according to the manufacturer’s protocol. Libraries from total RNA from individual samples were prepared following the standard procedures for the Illumina’s mRNA-Seq at Novogene in San Diego, CA, United States. The libraries were sequenced using the Illumina NovaSeq 6000 platform which generated 150 base-pair paired-end reads.

Quality of the sequencing reads was evaluated using FastQC (version 0.11.7, Babraham Bioinformatics, Cambridge, United Kingdom). Trimming was performed using Trim Galore (version 0.4.4, Babraham Bioinformatics, United Kingdom) with the following parameters: --paired --length 50 --clip_R1 15 --clip_R2 15 --three_prime_clip_R1 5 --three_prime_clip_R2 5. After processing, reads were mapped to the house mouse (Mus musculus) reference genome (GRCm39) using Hisat2 (v2.1.0) (Kim et al., 2015). The accession number GSE188845 can be used to access the sequencing data through NCBI GEO database.

### 2.4 RNA-Seq: Expression Analysis

The number of sequencing reads that mapped to each annotated gene in the annotation file (GTF file) was obtained using the python script htseq-count (v0.6.1p1) using the option intersection-nonempty (Anders et al., 2015). The differential expression analysis was performed using the *R* package edgeR (Robinson et al., 2010). This package combines 1) the use of the trimmed mean of M-values as normalization method, 2) the estimation of tagwise negative binomial dispersion values using an empirical Bayes approach, and finally 3) the fitting of negative binomial generalized log-linear models to detect significant genes. Here, the generalized log-linear linear models included the main effect of fluoxetine, the main effect of dietary FA, and the interaction effect fluoxetine-by-FA. The detection of differentially expressed genes in response to either fluoxetine, FA, or both, was conducted using likelihood ratio tests. Given that RNA-sequencing is now recognized as a mature, robust, and reliable technique, we did not validate the gene expression results using other techniques, such as qPCR.

### 2.5 Over-Representation Analysis

The over-representation or enrichment of gene-sets with differentially expressed genes was tested using Fisher’s exact test, a hypergeometric-based test commonly used to evaluate 2 × 2 contingency tables. Differentially expressed genes (*p*-value ≤ .01) that had ENSEMBL annotations were tested against the background set of all expressed genes with ENSEMBL annotations. Functional terms (gene-sets) from various databases, including Gene Ontology (GO), Kyoto Encyclopedia of Genes and Genomes (KEGG), and Medical Subject Headings (MeSH) were interrogated. All these analyses were performed using the R package EnrichKit (https://github.com/liulihe954/EnrichKit).

## 3 Results

Folic acid reverses fluoxetine-mediated increased mammary gland serotonin concentration without impacting circulating serotonin or calcium.

**FIGURE 1:**
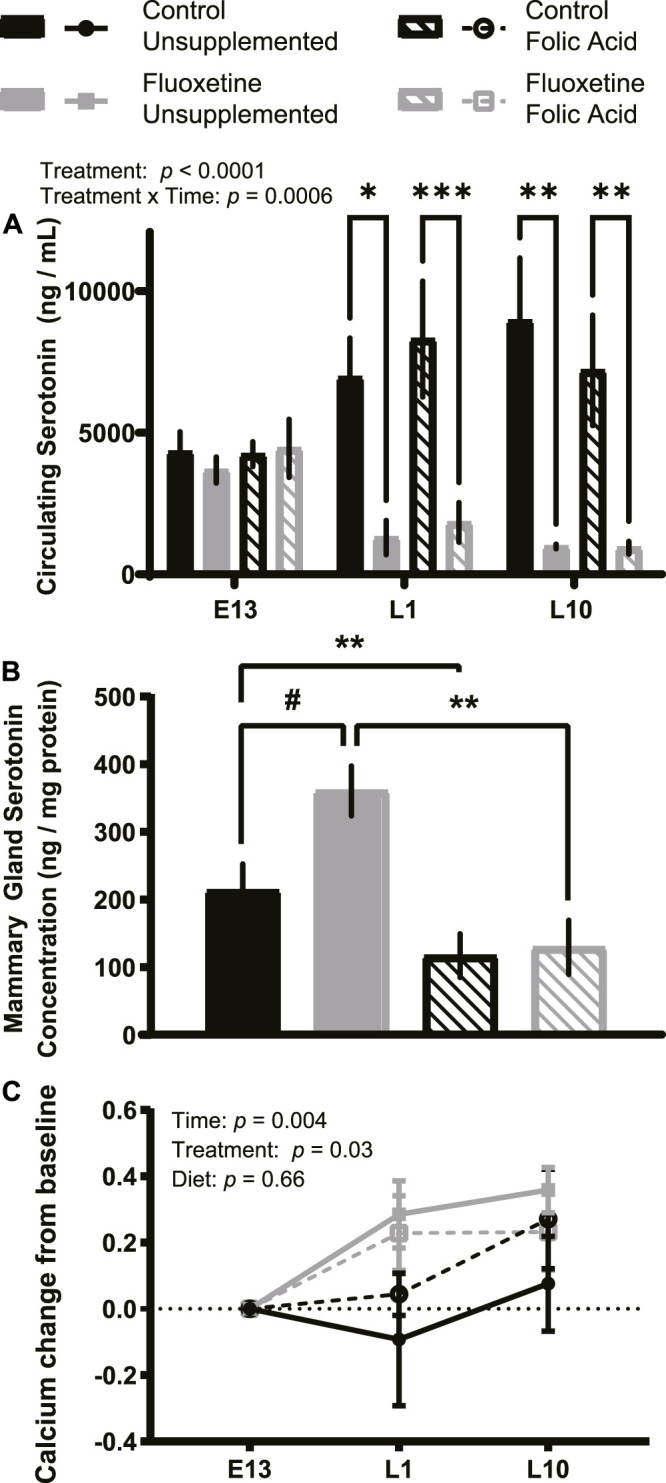
Circulating serotonin and calcium concentrations are impacted by fluoxetine but not folic acid (FA) supplementation. C57BL/6J mice were randomly assigned to a FA supplemented (24 mg/kg FA in diet) or breeder diet (4 mg/kg FA in diet) for 2 weeks prior to mating. On embryonic day 13 (E13), dams were randomly assigned into control (breeder diet, saline injection; *n* = 8), fluoxetine (breeder diet, 20 mg/kg/d fluoxetine injection; *n* = 8), FA (FA diet, saline injection; *n* = 8), or interaction (FA diet, 20 mg/kg/d fluoxetine injection; *n* = 8) groups. Blood samples were collected from all mice at the start of treatment on E13, lactation day 1 (L1), and lactation day 10 (L10), and serum was isolated. Serum serotonin (ng/ml) is shown **(A)**. Protein isolated from mammary glands were used to quantify mammary gland serotonin concentration (ng/mg protein) at L10 **(B)**. Serum calcium concentrations (nM) were generated by subtracting each mouse’s baseline (E13) serum calcium concentration from their serum calcium concentrations on L1 or L10 to correct for variation within each dam **(C)**. Data presented as mean ± SEM. ^#^ *p* < .1, *p < .05, **p < .01, ***p < .001, and ****p < .0001.

We examined the effects of peripartum fluoxetine treatment and dietary FA supplementation on maternal 5HT homeostasis. Maternal serum 5HT was decreased by fluoxetine treatment throughout the treatment period (L1 and L10) compared to start of treatment (E13) (*p* < .0001, Figure 1A). Serum 5HT concentrations did not vary over time, however there was an interaction between treatment and time (*p* < .001). This is consistent with previous studies demonstrating SSRI treatment reduces serum 5HT levels (Bismuth-Evenzal et al., 2012; Holck et al., 2019). FA supplementation did not impact circulating 5HT concentrations (*p* > .05).

Mammary 5HT concentration on L10 tended to increase in the fluoxetine-only treated group (*p* = .09; Figure 1B). Dams supplemented with FA had reduced mammary 5HT concentration compared to control dams (*p* < .05) and the dams treated with fluoxetine and supplemented with FA exhibited significantly reduced mammary 5HT concentration compared to fluoxetine-only (*p* < .01). Circulating calcium concentrations were altered over time (*p* < .01) and by fluoxetine treatment (*p* < .05), but not by FA supplementation (*p* > .05; Figure 1C).

### 3.1 Fluoxetine Alters the Mammary Gland Transcriptome

**FIGURE 2:**
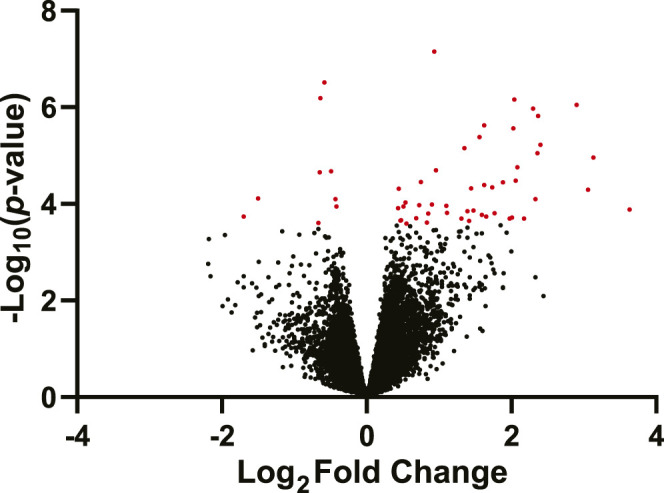
Volcano plot of differentially expressed genes in the mammary gland of mice treated with fluoxetine (20 mg/kg/d, *n* = 8) compared to saline control (*n* = 8). The y-axis denotes the −log_10_ *p*-value for each gene, while the x-axis denotes the log_2_ fold change for that gene relative to fluoxetine treatment. Red dots indicate significantly changed genes in the fluoxetine treatment group (FDR ≤10%); black dots indicate non-significantly changed genes or FDR ≥10%.

Fifty-six genes were differentially expressed (DEGs) between the fluoxetine and saline groups, of which 49 were upregulated and 7 were downregulated in fluoxetine treated dams relative to saline treated dams (False Discovery Rate (FDR) ≤ 10%, Figure 2; Supplementary Table S1). The most significantly affected DEG was *Elovl6* (ELOVL family member 6, elongation of long chain fatty acids, logFC = .45; *q-*value = .002), while the DEG with the largest fold change was *Saa2* (serum amyloid 2, logFC = 1.74, *q*-value = .076). The DEG with the second largest negative fold change was *Jazf1* (juxtaposed with another zinc finger protein 1, logFC = −.72, *q*-value = .06). Controlling using an FDR ≤10%, none of the genes showed differential expression due to FA supplementation. Similarly, none of the genes exhibited a significant fluoxetine treatment by FA supplementation interaction.

### 3.2 Fluoxetine Effects on Mammary Gland Pathways

**FIGURE 3:**
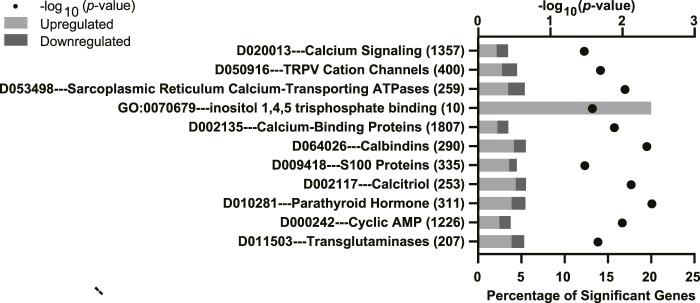
Significantly enriched calcium trafficking pathways from Gene Ontology (GO), Kyoto Encyclopedia of Genes and Genomes (KEGG), and Medical Subject headings (MeSH) pathway analyses in the mammary gland of mice treated with fluoxetine (20 mg/kg/d, *n* = 8) or saline control (*n* = 8) from embryonic day 13 through lactation day 10. The y-axis denotes the names and total number of genes in each pathway, and the lower x-axis denotes the percentage of significant genes within each pathway. The upper x-axis denotes the −log_10_ *p*-value. Light gray bars indicate upregulated DEGs and dark gray bars indicate downregulated DEGs within each pathway.

**FIGURE 4:**
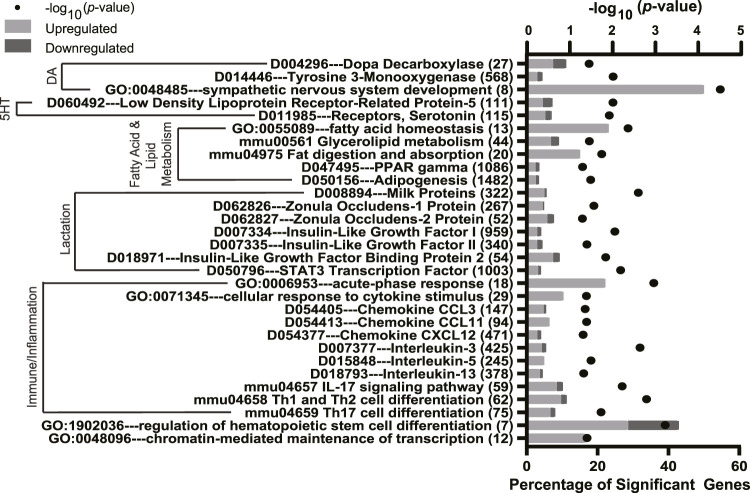
Significantly enriched pathways from Gene Ontology (GO), Kyoto Encyclopedia of Genes and Genomes (KEGG), and Medical Subject headings (MeSH) pathway analyses in the mammary gland of mice treated with fluoxetine (20 mg/kg/d, *n* = 8) or saline control (*n* = 8) from embryonic day 13 through lactation day 10. The y-axis denotes the names and total number of genes in each pathway, and the lower x-axis denotes the percentage of significant genes within each pathway. The upper x-axis denotes the −log_10_ *p*-value. Light gray bars indicate upregulated DEGs and dark gray bars indicate downregulated DEGs within each pathway. Abbreviations: DA, dopamine; 5HT, serotonin; IL, interleukin.

To further characterize the effect of fluoxetine on the mammary gland, the potential enrichment of GO, KEGG, and MeSH terms with DEGs was analyzed using a Fisher’s exact test. The full list of significant terms is reported in Supplementary Tables S2–S4. Calcium signaling pathways were impacted by fluoxetine treatment (Figure 3) which are important for mammary epithelial calcium trafficking for milk synthesis (e.g., *D010281, parathyroid hormone, GO:0070679 inositol 1,4,5 triphosphate binding, D064026 calbindins*, and *D020013 calcium signaling*). Notably, several functional terms related to lactation were altered by fluoxetine treatment (Figure 4). Fatty acid and lipid metabolism were among the most abundantly enriched pathways (e.g., *GO:0055089 fatty acid homeostasis, mmu00561 glycerolipid metabolism*, and *D047495 PPAR gamma*), along with immune pathways (e.g., *GO:0006953 acute-phase response, mmu04658 Th1 and Th2 cell differentiation*, and *GO:0071345 cellular response to cytokine stimulus*). Dopamine and serotonin pathways were enriched (*D014446 tyrosine 3-monooxygenase, D004296 dopa decarboxylase, D060492 low density lipoprotein receptor-related protein 5*, and *D011985 receptors, serotonin*), as well as pathways related to milk protein synthesis and tight junctions (*D008894 milk proteins* and *D062826 zonula occludens-1 protein*) in mammary glands exposed to fluoxetine.

### 3.3 Effects of Folic Acid Supplementation on Pathways in the Mammary Gland

**FIGURE 5:**
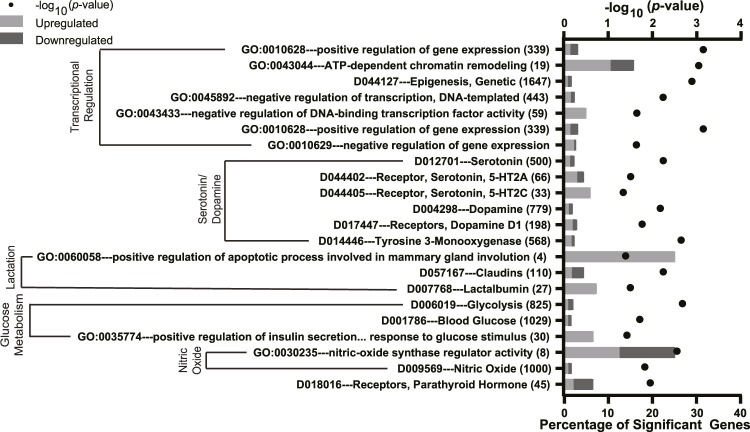
Significantly enriched Gene Ontology (GO), Kyoto Encyclopedia of Genes and Genomes (KEGG), and Medical Subject headings (MeSH) pathways in the mammary gland of mice with folic acid (FA) supplementation (24 mg/kg FA in diet) or breeder diet (4 mg/kg FA in diet) for 5 ± 2 weeks. The y-axis denotes the names and total number of genes in each pathway, and the lower x-axis denotes the percentage of significant genes within each pathway. The upper x-axis denotes the −log_10_ *p*-value. Light gray bars indicate upregulated DEGs and dark gray bars indicate downregulated DEGs within each pathway.

We also assessed the role of FA supplementation on mammary gland gene expression using an over-representation analysis. The full list of significant terms is reported in Supplementary Tables S5–S7. We identified 22 pathways of interest that were altered by FA supplementation (Figure 5). The most enriched pathways were those involving transcriptional regulation (e.g., *GO:0010628 positive regulation of gene expression, GO:0043044 ATP-dependent chromatin remodeling*, and *D044127 epigenesis, genetic*). There were several significant terms related to lactation, including milk production (*GO:0060058 positive regulation of apoptotic process involved in mammary gland involution* and *D007768 lactalbumin*) and glucose metabolism (*D006019 glycolysis* and *D00786 blood glucose*). FA supplementation also impacted terms associated with 5HT and dopamine pathways (e.g., *D012701 serotonin, D044402 serotonin 5-HT2A receptor* and *D004298 dopamine, D017447 dopamine D1 receptors*). FA supplementation downregulated *D019016 receptors, parathyroid hormone*.

### 3.4 Common Pathways Between Fluoxetine Treatment and Folic Acid Supplementation

**FIGURE 6:**
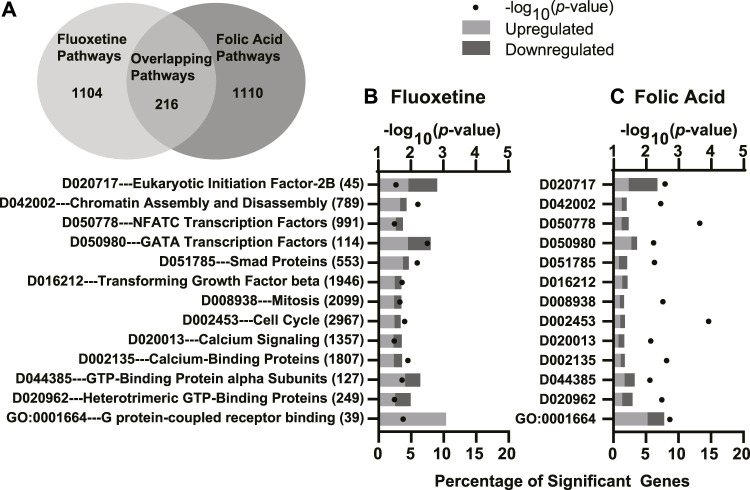
Significantly enriched Gene Ontology (GO), Kyoto Encyclopedia of Genes and Genomes (KEGG), and Medical Subject headings (MeSH) pathways simultaneously enriched in both comparisons for fluoxetine treatment and folic acid (FA) supplementation. There were 216 overlapping pathways between the two comparisons **(A)**. GO, KEGG, and MeSH enriched pathways in the mammary glands of mice treated with fluoxetine (20 mg/kg/d) compared to saline control **(B)** and FA supplementation (24 mg/kg FA diet) compared to control (4 mg/kg FA diet) **(C)**. The y-axis denotes the names and total number of genes in each pathway, and the lower x-axis denotes the percentage of significant genes within each pathway. The upper x-axis denotes the −log_10_ *p*-value. Light gray bars indicate upregulated DEGs and dark gray bars indicate downregulated DEGs within each pathway.

The GO overrepresentation analysis identified one common pathway shared between fluoxetine treatment and FA supplementation. *G-coupled protein receptor binding* was upregulated in mammary glands of dams receiving fluoxetine treatment and FA supplementation. The MeSH analysis identified 215 overlapping pathways (Figure 6A). Combining all analyses, we identified 12 functional terms of interest overlapping between fluoxetine treatment and FA supplementation (Figure 6B). These significant terms included G-protein signaling (*GO:0001664 G protein-coupled receptor binding* and *D044385 GTP-binding protein alpha subunits*), calcium signaling (*D020013 calcium signaling* and *D002135 calcium-binding proteins*), cell cycle progression (*D002453 cell cycle* and *D008938 mitosis*) and cell proliferation (*D016212 transforming growth factor beta* and *D051785 smad proteins*). In addition, the combination of both treatments altered pathways involving transcriptional regulation (*D042002 chromatin assembly and disassembly*).

## 4 Discussion

Mammary 5HT signaling regulates PTHrP synthesis and calcium signaling during lactation (Hernandez et al., 2012a; Laporta et al., 2014a). In *Tph1*-KO mice, the methylation status of the sonic hedgehog (SHH) promoters in the mammary gland were altered compared to wildtypes, which was rescued by treatment with the 5HT precursor, 5HTP (Laporta et al., 2014b). Furthermore, peripartal fluoxetine treatment resulted in hypomethylation of the PTHrP promoter in the mammary gland (Weaver et al., 2018). Therefore, we sought to investigate the transcriptome changes in the mammary gland during peak lactation with fluoxetine treatment and further determine whether supplementation with a known methyl donor, FA, would mitigate the fluoxetine-induced signaling.

SSRIs bind to SERT, which is located both centrally and peripherally, increasing the exposure of the neuron or peripheral tissue to 5HT. Consistent with this, fluoxetine increased mammary 5HT concentrations while decreasing circulating 5HT. Platelets, which transport 5HT in the circulation, lack the tryptophan hydroxylase enzyme required for *de novo* 5HT synthesis, and therefore can only acquire 5HT through SERT (Mercado and Kilic, 2010). Thus, treatment with an SSRI reduces blood 5HT concentrations, which is in congruence with previous studies by our lab and others (Weaver et al., 2018; Holck et al., 2019).

Although SSRIs like fluoxetine inhibit SERT, fluoxetine enriched pathways in the mammary gland involved in dopamine synthesis and metabolism such as *dopa decarboxylase*, which also decarboxylates 5HTP to produce 5HT, and *tyrosine 3-monooxygenase*. Furthermore, the *sympathetic nervous system development* GO pathway was upregulated by fluoxetine treatment. Fluoxetine increases norepinephrine and dopamine in addition to 5HT in the prefrontal cortex of rats (Bymaster et al., 2002). Plasma dopamine concentrations are largely attributed to sympathetic nerves, which release the dopamine precursor and noradrenaline (Goldstein and Holmes, 2008), and fluoxetine can modulate the sympathetic nervous system during hypoglycemia in healthy adults (Briscoe et al., 2008). Thus, fluoxetine increases in dopamine signaling during lactation warrants future research regarding the interaction between 5HT and dopamine during pregnancy and lactation.

SSRIs alter both central and peripheral 5HT concentrations, and 5HT is vital in calcium homeostasis during lactation (Laporta et al., 2014a). Herein, fluoxetine increased circulating calcium concentrations and enriched multiple pathways in the mammary gland related to calcium signaling. Intricate calcium trafficking in the mammary gland, including calcium channels, receptors, binding proteins, pumps, transporters and hormones, ensures calcium is delivered to the neonate via milk (Grinman et al., 2020). One such model of calcium transfer, termed CALTRANS, occurs via the calcium release-activated calcium channel protein 1 (ORAI1) transporter, and potentially the transient receptor potential cation channels (TRPV) channels, moving calcium from the blood into the mammary epithelial cell (Cross et al., 1995). Calcium can then be sequestered into the endoplasmic reticulum via the sarco/endoplasmic reticulum calcium ATPase (SERCA2) transporter and subsequently released from the endoplasmic reticulum through 1,4,5 inositol triphosphate (IP_3_) binding to the IP_3_ receptor. In the present study, multiple pathways involved in CALTRANS were upregulated (e.g., *TRPV* *cation channels*, *SERCA*, and *IP*_*3*_ *binding*). Calcium is then transported into milk following binding to milk proteins or directly through the PMCA2 transporter, which is aided by calcium binding proteins such as calbindin and calmodulin (Cross et al., 1995). Fluoxetine upregulated multiple pathways involved in intracellular calcium transportation during lactation (e.g., *calcium-binding proteins* and *calbindins*). These upregulated calcium flux pathways, combined with the increased circulating calcium with fluoxetine treatment, could theoretically increase milk calcium concentrations and should be examined in future studies.

Mammary-derived PTHrP regulates calcium homeostasis during lactation in part through 5HT signaling (VanHouten et al., 2004; Hernandez et al., 2012a). Fluoxetine upregulated the *parathyroid hormone* pathway in the mammary gland, as well as *cyclic AMP* pathway. The *Pthr1* gene was upregulated within the *cyclic AMP* pathway. PTHrP can stimulate cyclic AMP (cAMP) production in mammary epithelial cells, suggesting that PTHrP not only has endocrine functions to liberate calcium from bone, but also has autocrine/paracrine functions in the mammary gland through binding to mammary PTHR1 (Ferrari et al., 1993). In the present study, fluoxetine upregulated the *transglutaminases* pathway, suggesting regulation of serotonylation. During serotonylation, 5HT is covalently linked to a target protein, often small G-proteins, through the calcium-dependent enzyme transglutaminase (Muma and Mi, 2015). Recently, we demonstrated that transglutaminase-dependent protein serotonylation is an important intermediate step in PTHrP induction *in vitro* in mammary epithelial cells treated with fluoxetine or 5HTP (Sheftel and Hernandez, 2020), but further investigation is required to determine the identity of the modified proteins and how modifying the serotonylation substrate(s) with SSRIs ultimately influences calcium metabolism.

During lactation, the mammary gland is a lipid-metabolizing organ, producing free fatty acids for milk synthesis (Suburu et al., 2014). The most altered gene by fluoxetine treatment was *Elovl6*, which is involved in the elongation of fatty acids as well as energy metabolism. Additionally, fatty acid and lipid metabolism pathways were some of the most numerous fluoxetine-enriched pathways in all analyses. In the Fatty acid homeostasis pathway, there were 3 upregulated genes (*Dgat1, Dgat2*, and *Pold1*). *Dgat1* and *Dgat2* are diacylglycerol O-acyltransferases, which are involved in the conversion of diacylglycerol (DAG) to triacylglycerol (TAG) (Cases et al., 1998). The onset of lactation results in significant upregulation of lipid synthesis genes, resulting in milk containing 30% lipid content as TAGs (Rudolph et al., 2007). A previous transcriptomic study by our lab demonstrated that 5HT-depletion in *Tph1*-KO mice downregulated genes involved in lipid metabolism (Cheng et al., 2020). Both studies together demonstrate the impact of 5HT on lipid metabolism during lactation. Future studies should be directed towards elucidating role of SSRIs in milk fat synthesis.

The mammary fat pad, a mix of adipose and connective tissue, is essential for mammary epithelium development but also undergoes remodeling during the peripartum period. Activation of the 5HT receptor 2A is necessary for the early adipocyte differentiation process, and mediates expression of adipogenic genes such as peroxisome proliferator-activator receptor γ (*Ppar-*γ) (Yu et al., 2021). Fluoxetine increased *PPAR-γ* and *adipogenesis* pathways. In addition, fluoxetine downregulated the *Jazf1* gene, a co-repressor, linked to glucose and lipid metabolism, which reduces PPAR-γ expression in adipocytes (Ming et al., 2014). 5HT metabolites can act as endogenous agonists for PPAR-γ to regulate adipogenesis (Watanabe et al., 2011). Increased adipogenesis is associated with obesity and mice fed a high fat diet display an obese phenotype in the mammary gland with increased adipocytes (Hernandez et al., 2012b). This phenotype can be alleviated with 5HT-deficiency (Weaver et al., 2016), suggesting 5HT may modulate the effects of obesity. Prepartum obesity can delay the onset of lactation (Flint et al., 2005), which can be a barrier to breastfeeding. In addition, fluoxetine has been shown to delay lactogenesis as well as accelerate mammary gland involution (Marshall et al., 2010). Together, these data raise questions about the impact of peripartal fluoxetine use on successful lactation.

Fluoxetine enriched pro-lactation pathways and potential anti-lactation pathways. 5HT has biphasic actions on mammary gland tight junctions that are concentration and time-dependent (Pai and Horseman, 2008). Due to the numerous 5HT receptors that exist, this allows for complex physiological effects of 5HT on a multitude of potential pathways in various tissues (Nichols and Nichols, 2008). In the present study, *milk protein* pathway and tight junction pathways (*zonula occludens-1 protein*, *zonula occludens-2 protein*) were upregulated by fluoxetine, suggesting that milk production pathways are not being limited in the mammary glands of fluoxetine-treated dams. Further, fluoxetine also upregulated multiple IGF pathways, including *insulin-like growth factor I*, which can delay mammary gland involution (Neuenschwander et al., 1996). However, fluoxetine simultaneously upregulated *signal transducer and activator of transcription 3* (*Stat3*) pathway. STAT3 is induced as mammary epithelial cells undergo involution (Clarkson and Watson, 2003). These results suggest that the timing of the initiation of SSRI treatment and the duration of SSRI treatment may have differential impacts on the mammary gland. Indeed, during mammary gland involution the primary molecular signature displayed is inflammation and the acute phase response (Clarkson and Watson, 2003). Fluoxetine upregulated proinflammatory mediator pathways such as *cellular response to cytokine stimulus, chemokine *pathways, *t helper cell (−1, −2, and −17) differentiation,* and the downstream *interleukin* pathways. Stimulation of mammary gland inflammation pathways by fluoxetine treatment should be further examined in peripartal women taking SSRIs as they may result in delayed onset of lactation and/or earlier weaning.

In addition to inflammation-mediated involution, 5HT is also involved in a variety of immunomodulatory functions including chemotaxis, cytokine secretion, and leukocyte activation (Arreola et al., 2015). Here, fluoxetine impacted numerous genes and pathways involved in immune system function, including SAA genes (*Saa1*, *Saa2*, and *Saa3*). SAA proteins are apolipoproteins associated with high density lipoprotein in the plasma and are part of the acute phase inflammation response that occurs upon macrophages releasing pro-inflammatory cytokines (Sipe et al., 1979; Coetzee et al., 1986). In a previous study, mice fed a high fat diet exhibited increased 5HT signaling and elevated inflammatory markers in the mammary gland, suggesting a role for 5HT on inflammation in high fat diet-induced obesity (Hernandez et al., 2012b).

Fluoxetine treatment upregulated pathways involving transcription, including *chromatin-mediated maintenance of transcription*. We therefore sought to alter DNA methylation using a known DNA methyl donor, FA. Interestingly, FA supplementation did not result in any significantly altered gene after controlling for multiple testing, suggesting that any FA effects on the mammary gland may be more diffuse. We then performed gene-set analysis using a more liberal threshold (*p*-value < .01), which revealed insights as to how FA supplementation throughout the peripartal period may impact the mammary gland. Unsurprisingly, the most numerously enriched pathways involve transcriptional regulation (e.g., *positive regulation of gene expression, ATP-dependent chromatin remodeling, epigenesis*, and *negative regulation of gene expression*). Positive regulation of gene expression was downregulated while negative regulation of gene expression was upregulated, together suggesting decreased overall gene expression. Methylation of DNA cytosine bases results in a compaction of the chromatin and inaccessibility of transcription factors from binding to their regulatory elements, thereby reducing transcription (Attwood et al., 2002). As FA is a known methyl donor, these results suggest that FA supplementation may alter gene expression in the mammary gland, potentially through DNA methylation.

Similar to fluoxetine, FA supplementation enhanced both 5HT pathways (e.g., *serotonin* and *serotonin receptor* *5HT2A* and *5HT2C*) as well as dopamine pathways (e.g., *dopamine, dopamine receptor 1,* and *tyrosine 3—monooxygenase*). Despite upregulation 5HT pathways, mammary 5HT concentrations were reduced by FA supplementation and the effect of fluoxetine treatment combined with FA supplementation reduced mammary 5HT concentrations compared to fluoxetine treatment alone. However, 5HT receptor pathways were increased, which may have effects on specific 5HT receptor abundance with FA supplementation. There are 6 5HT receptor subtypes in the mammary gland, so it is possible that there may be compensatory expression of these 5HT receptors to maintain critical signaling pathways (Nichols and Nichols, 2008; Hernandez et al., 2009; Pai et al., 2009).

FA is needed during pregnancy for proper development of the brain and spinal cord in the developing infant and fortification of various foods in the 1990’s significantly reduced neural tube defects (Czeizel and Dudás, 1992; Honein et al., 2001; De Wals et al., 2007). Little is known regarding the effects of FA supplementation during lactation and the impact on the mammary gland in rodents and humans. In the present study, FA supplementation enriched lactation pathways (e.g., *positive regulation of apoptotic process involved in mammary gland involution, claudins*, and *lactalbumin*). Claudins are a family of proteins that are components of the tight junctions necessary for lactation (Kobayashi and Kumura, 2011). α-Lactalbumin is a milk protein that is critical for the synthesis of lactose, the major sugar in human breast milk that is critical for osmoregulation of milk (Lönnerdal and Lien, 2003). The upregulation of involution and downregulation of claudins pathway suggests that FA may have a critical role in mammary cell turnover, which maintains milk synthesis.

The mammary gland is a key organ for calcium flux during lactation, when calcium is drawn from bone stores and kidney resorption from urine to the mammary gland to supply milk calcium. Fluoxetine and FA both impacted calcium signaling pathways (e.g., *calcium signaling* and *calcium-binding proteins*). However, only the fluoxetine treatment increased circulating calcium levels in the dam. Notably, fluoxetine upregulated the *Pthr1* gene in multiple pathways including the parathyroid hormone pathway, but FA downregulated the *parathyroid hormone receptors* pathway, which could suggest a mitigating role for FA in fluoxetine treatment. The parathyroid hormone receptor pathway is not only important in the mammary gland and kidney, but it is also the receptor for PTHrP on the bone. As an exploratory analysis, we therefore examined genes and pathways in these mammary cells that are known to also be important in calcium flux in bone. Although we cannot definitively say that signaling in the bone will occur similarly to that in the mammary gland in response to fluoxetine and/or FA, we observed alterations in genes important for bone turnover. Fluoxetine altered multiple genes that resulted in bone pathways being enriched in the mammary gland. Multiple pathways involving differentiating hematopoietic precursors were altered, including *regulation of hematopoietic stem cell differentiation*, which is relevant in mammary cells for immunomodulatory functions. If these same genes/pathways were upregulated by fluoxetine in the bone, it is plausible that this could result in increased osteoclastogenesis, since osteoclasts are derived from hematopoietic stem cells (Yavropoulou and Yovos, 2008), supporting our previous results that peripartum fluoxetine use exacerbated maternal bone loss postweaning (Weaver et al., 2018). Together, these results warrant additional studies to determine the alterations in the transcriptome in the bone with fluoxetine and/or FA. Functional studies determining bone microarchitecture are needed to determine whether FA supplementation could mitigate the fluoxetine-mediated bone loss during lactation.

There are several limitations to this study that should be addressed in future studies, such as milk composition and bone microarchitecture with fluoxetine and/or FA supplementation. Additionally, histological examination using immunofluorescence should be used in future studies to determine how fluoxetine treatment impacts mammary involution since both pro-lactation and anti-lactation pathways were enriched. The mice used in this study are wildtype mice and therefore did not need SSRI treatment to ameliorate depressive symptoms. 5HT is unable to cross the blood brain barrier and there are two TPH enzymes depending on neuronal or peripheral location, together resulting in two distinct pools of 5HT: neuronal and peripheral (Walther et al., 2003). In the present study, we are examining how SSRI treatment alters the peripheral serotonergic system, particularly in the mammary gland, rather than neuronal 5HT. Therefore, this system would be altered independent of depressive symptoms.

## 5.Conclusion

In summary, peripartal treatment with an SSRI perturbs the peripheral serotonergic system, which is independent of neuronal 5HT, finding fluoxetine reduced circulating 5HT concentrations, increased circulating calcium concentrations, and increased mammary 5HT concentration. Fluoxetine altered genes and gene-sets (5HT and dopamine metabolism, calcium trafficking, lactation, lipid metabolism, and immune function) which revealed mechanisms impacted by peripartal SSRIs in the mammary gland. Notably, fluoxetine enriched pathways could positively or negatively impact lactation, raising questions on whether timing and duration of treatment may have differential effects on lactation success. FA supplementation did not significantly impact specific genes with or without the SSRI, contrary to our hypothesis that FA supplementation could reverse SSRI-induced altered DNA methylation and downstream transcriptional changes. However, FA supplementation did alter several pathways, such as milk protein production which could be beneficial to mothers already taking FA as a preventative for neural tube defects according to US guidelines (Honein et al., 2001). Future research should examine the impacts of FA supplementation on mammary gland-bone signaling during lactation to determine whether FA supplementation may reverse possible excessive bone loss due to lactation and/or SSRI treatments through diffuse pathway effects or downstream mechanisms rather than affecting individual genes.

## Data Availability

The datasets presented in this study can be found in online repositories. The names of the repository/repositories and accession number(s) can be found in the article/Supplementary Material.

## Abbreviations

5HTP, 5-hydroxytrypamine; ORAI1, Calcium release-activated calcium channel protein 1; DAG, Diacylglycerol; DEG, Differentially expressed genes; ELOVL6, Elongation of long chain fatty acids family member 6; FDR, False discovery rate; FA, Folic acid; GO, Gene ontology; IP_3_, I,4,5 inositol triphosphate; IGF, Insulin-like growth factor; JAZF1, Juxtaposed with another zinc finger protein 1; KO, Knockout; KEGG, Kyoto encyclopedia of genes and genomes; LRP5Low density lipoprotein receptor-related protein 5; MeSH, Medical subject headings; PTHR1, Parathyroid hormone receptor type 1; PTHrP, Parathyroid hormone related protein; PTH, Parathyroid hormone; PPAR-γ, Peroxisome proliferator-activator receptor γ; SERCA2, Sarco/endoplasmic reticulum calcium ATPase; SSRI, Selective serotonin reuptake inhibitor; SERT, Serotonin reuptake transporter; 5HT, Serotonin; SAA, Serum amyloid; TRPV, Transient receptor potential cation channels; TAG, Triacylglycerol; TPH, Tryptophan hydroxylase.
