# Supplementary material for: Impact of Fluoxetine Treatment and Folic Acid Supplementation on the Mammary Gland Transcriptome During Peak Lactation
Source: Front Pharmacol. 2022 Feb 23;13:828735. doi: 10.3389/fphar.2022.828735 (PMC8904566; doi:10.3389/fphar.2022.828735)
Supplement: Supplementary file 1 [file DataSheet1.docx]

Supplementary Material

# Supplementary Data

**Supplemental Table 1.** Differentially expressed genes (DEGs) with fluoxetine treatment (FDR ≤ 20%). Sorted most significant *q*-value to least significant.

|  | Differentially Expressed Genes: Fluoxetine Treatment 20% FDR | | | | | |
| --- | --- | --- | --- | --- | --- | --- |
| **Gene Name** | | **Gene Abbr.** | **Ensembl code** | **logFC** | ***p*-value** | **Q-Value** |
| ELOVL family member 6, elongation of long chain fatty acids (yeast) | | Elovl6 | ENSMUSG00000041220 | 0.45 | 1.46E-07 | 2.05E-03 |
| serine hydrolase-like | | Serhl | ENSMUSG00000058586 | -0.28 | 6.00E-07 | 4.21E-03 |
| asparaginase | | Aspg | ENSMUSG00000037686 | -0.31 | 1.23E-06 | 4.59E-03 |
| phosphoglucomutase 5 | | Pgm5 | ENSMUSG00000041731 | 0.98 | 1.31E-06 | 4.59E-03 |
| orosomucoid 2 | | Orm2 | ENSMUSG00000061540 | 1.40 | 1.67E-06 | 4.65E-03 |
| Gm47445 TEC? | |  | ENSMUSG00000111361 | 1.10 | 1.99E-06 | 4.65E-03 |
| secretory leukocyte peptidase inhibitor | | Slpi | ENSMUSG00000017002 | 1.14 | 2.79E-06 | 5.60E-03 |
| Gm27010 LncRNA | |  | ENSMUSG00000098183 | 0.78 | 4.24E-06 | 7.44E-03 |
| Gm43813 LncRNA | |  | ENSMUSG00000106636 | 0.98 | 4.88E-06 | 7.62E-03 |
| complement component 4B (Chido blood group) | | C4b | ENSMUSG00000073418 | 0.75 | 7.35E-06 | 1.03E-02 |
| Gm42876 TEC? | |  | ENSMUSG00000105691 | 1.15 | 1.03E-05 | 1.32E-02 |
| Gm12953 LncRNA | |  | ENSMUSG00000087299 | 0.65 | 1.22E-05 | 1.42E-02 |
| Gm49474 TEC? | |  | ENSMUSG00000116021 | 1.14 | 1.52E-05 | 1.64E-02 |
| KIT proto-oncogene receptor tyrosine kinase | | Kit | ENSMUSG00000005672 | 1.50 | 1.87E-05 | 1.88E-02 |
| WAP four-disulfide core domain 17 | | Wfdc17 | ENSMUSG00000069792 | 1.00 | 2.90E-05 | 2.72E-02 |
| coiled-coil domain containing 163 | | Ccdc163 | ENSMUSG00000028689 | 0.46 | 3.33E-05 | 2.84E-02 |
| sodium channel, nonvoltage-gated 1 beta | | Scnn1b | ENSMUSG00000030873 | -0.23 | 3.48E-05 | 2.84E-02 |
| methyltransferase like 7A1 | | Mettl7a1 | ENSMUSG00000054619 | -0.31 | 3.64E-05 | 2.84E-02 |
| Gm17160 LncRNA? | |  | ENSMUSG00000090329 | 0.99 | 5.39E-05 | 3.85E-02 |
| A130071D04Rik TEC? | |  | ENSMUSG00000104291 | 0.36 | 5.67E-05 | 3.85E-02 |
| D630033A02Rik psuedogene? | |  | ENSMUSG00000112237 | 0.90 | 5.76E-05 | 3.85E-02 |
| transcription elongation factor A (SII), 2 | | Tcea2 | ENSMUSG00000059540 | 0.78 | 6.52E-05 | 4.16E-02 |
| bromodomain adjacent to zinc finger domain, 1B | | Baz1b | ENSMUSG00000002748 | 0.83 | 7.33E-05 | 4.37E-02 |
| leucine-rich alpha-2-glycoprotein 1 | | Lrg1 | ENSMUSG00000037095 | 0.69 | 7.61E-05 | 4.37E-02 |
| RIKEN cDNA A530016L24 gene | |  | ENSMUSG00000043122 | 0.21 | 7.77E-05 | 4.37E-02 |
| serum amyloid A 1 | | Saa1 | ENSMUSG00000074115 | 1.47 | 8.10E-05 | 4.37E-02 |
| pyridoxal-dependent decarboxylase domain containing 1 | | Pdxdc1 | ENSMUSG00000022680 | -0.72 | 1.21E-04 | 6.09E-02 |
| serum amyloid A 3 | | Saa3 | ENSMUSG00000040026 | 1.12 | 1.24E-04 | 6.09E-02 |
| JAZF zinc finger 1 | | Jazf1 | ENSMUSG00000063568 | -0.21 | 1.26E-04 | 6.09E-02 |
| histocompatibility 2, Q region locus 4 | | H2-Q4 | ENSMUSG00000035929 | 0.26 | 1.45E-04 | 6.77E-02 |
| cytochrome b-245, alpha polypeptide | | Cyba | ENSMUSG00000006519 | 0.43 | 1.59E-04 | 7.08E-02 |
| poly(A) binding protein, cytoplasmic 4 | | Pabpc4 | ENSMUSG00000011257 | 0.35 | 1.64E-04 | 7.08E-02 |
| testin LIM domain protein | | Tes | ENSMUSG00000029552 | 0.53 | 1.69E-04 | 7.08E-02 |
| protein kinase C, epsilon | | Prkce | ENSMUSG00000045038 | -0.20 | 1.75E-04 | 7.08E-02 |
| AI463229 LncRNA? | |  | ENSMUSG00000114828 | 0.25 | 1.76E-04 | 7.08E-02 |
| pleckstrin homology domain containing, family J member 1 | | Plekhj1 | ENSMUSG00000035278 | 0.21 | 1.89E-04 | 7.37E-02 |
| serum amyloid A 2 | | Saa2 | ENSMUSG00000057465 | 1.74 | 2.02E-04 | 7.66E-02 |
|  | |  | ENSMUSG00000085078 | 0.71 | 2.09E-04 | 7.73E-02 |
| small nucleolar RNA host gene 11 | | Snhg11 | ENSMUSG00000044349 | 0.67 | 2.18E-04 | 7.86E-02 |
|  | |  | ENSMUSG00000038775 | 0.53 | 2.36E-04 | 8.11E-02 |
| sterile alpha motif domain containing 5 | | Samd5 | ENSMUSG00000060487 | 0.85 | 2.37E-04 | 8.11E-02 |
| Cbp/p300-interacting transactivator with Glu/Asp-rich carboxy-terminal domain 1 | | Cited1 | ENSMUSG00000051159 | 0.41 | 2.43E-04 | 8.14E-02 |
| Gm3235 LncRNA? | |  | ENSMUSG00000090778 | 0.77 | 2.56E-04 | 8.37E-02 |
| ferredoxin reductase | | Fdxr | ENSMUSG00000018861 | -0.82 | 2.76E-04 | 8.58E-02 |
| suprabasin | | Sbsn | ENSMUSG00000046056 | 0.79 | 2.78E-04 | 8.58E-02 |
| potassium large conductance calcium-activated channel, subfamily M, alpha member 1 | | Kcnma1 | ENSMUSG00000063142 | 0.96 | 2.92E-04 | 8.58E-02 |
| Mir99a and Mirlet7c-1 host gene (non-protein coding) | | Mir99ahg | ENSMUSG00000090386 | 0.33 | 3.00E-04 | 8.58E-02 |
| Gm37490 TEC? | |  | ENSMUSG00000102211 | 0.95 | 3.02E-04 | 8.58E-02 |
| Gm49539 LncRNA? | |  | ENSMUSG00000116004 | 0.63 | 3.04E-04 | 8.58E-02 |
| Gm53028 LncRNA? | |  | ENSMUSG00000116590 | 1.04 | 3.06E-04 | 8.58E-02 |
| catechol-O-methyltransferase | | Comt | ENSMUSG00000000326 | 0.23 | 3.28E-04 | 8.96E-02 |
| transformer 2 alpha | | Tra2a | ENSMUSG00000029817 | 0.23 | 3.32E-04 | 8.96E-02 |
| pseudogene? | |  | ENSMUSG00000118642 | 0.68 | 3.39E-04 | 8.97E-02 |
|  | |  | ENSMUSG00000024909 | 0.40 | 3.67E-04 | 9.48E-02 |
| secreted frizzled-related protein 2 | | Sfrp2 | ENSMUSG00000027996 | -0.32 | 3.71E-04 | 9.48E-02 |
| transmembrane channel-like gene family 5 | | Tmc5 | ENSMUSG00000030650 | 0.26 | 3.81E-04 | 9.55E-02 |
| cyclin-dependent kinase 9 (CDC2-related kinase) | | Cdk9 | ENSMUSG00000009555 | 0.89 | 4.16E-04 | 1.01E-01 |
| Gm11510, psuedogene | |  | ENSMUSG00000084411 | 0.20 | 4.18E-04 | 1.01E-01 |
| T cell, immune regulator 1, ATPase, H+ transporting, lysosomal V0 protein A3 | | Tcirg1 | ENSMUSG00000001750 | 0.29 | 4.36E-04 | 1.04E-01 |
| homeobox B6 | | Hoxb6 | ENSMUSG00000000690 | 0.67 | 4.62E-04 | 1.08E-01 |
| branched chain ketoacid dehydrogenase E1, alpha polypeptide | | Bckdha | ENSMUSG00000060376 | -0.32 | 4.89E-04 | 1.13E-01 |
| solute carrier family 36 (proton/amino acid symporter), member 2 | | Slc36a2 | ENSMUSG00000020264 | 0.46 | 5.13E-04 | 1.16E-01 |
| neuropilin 1 | | Nrp1 | ENSMUSG00000025810 | 0.45 | 5.19E-04 | 1.16E-01 |
| growth arrest and DNA-damage-inducible 45 alpha | | Gadd45a | ENSMUSG00000036390 | 0.63 | 5.43E-04 | 1.18E-01 |
| Btf3-ps1, pseudogene | |  | ENSMUSG00000089782 | -0.56 | 5.49E-04 | 1.18E-01 |
| 9930017N22Rik, TEC | |  | ENSMUSG00000115276 | 0.62 | 5.54E-04 | 1.18E-01 |
| NIN1/RPN12 binding protein 1 homolog | | Nob1 | ENSMUSG00000003848 | 0.21 | 5.63E-04 | 1.18E-01 |
| BCL2-like 2 or poly(A) binding protein, nuclear 1 | | Bcl2l2 or Pabpn1 | ENSMUSG00000022194 | 0.33 | 5.77E-04 | 1.18E-01 |
| cDNA sequence BC006965 | |  | ENSMUSG00000041674 | 0.93 | 5.83E-04 | 1.18E-01 |
| Gm10052, pseudogene | |  | ENSMUSG00000058922 | -0.35 | 5.91E-04 | 1.18E-01 |
| Gm37033, TEC | |  | ENSMUSG00000104388 | 0.77 | 5.98E-04 | 1.18E-01 |
| homeobox C8 | | Hoxc8 | ENSMUSG00000001657 | 0.73 | 6.09E-04 | 1.19E-01 |
| opticin | | Optc | ENSMUSG00000010311 | -0.44 | 6.31E-04 | 1.21E-01 |
| eukaryotic translation initiation factor 5B | | Eif5b | ENSMUSG00000026083 | -0.94 | 6.44E-04 | 1.22E-01 |
| immunoglobulin kappa chain variable 13-85 | | Igkv13-85 | ENSMUSG00000079543 | 0.27 | 6.51E-04 | 1.22E-01 |
| heterogeneous nuclear ribonucleoprotein H3 | | Hnrnph3 | ENSMUSG00000020069 | -0.28 | 6.84E-04 | 1.22E-01 |
| nidogen 2 | | Nid2 | ENSMUSG00000021806 | 0.48 | 6.87E-04 | 1.22E-01 |
| diacylglycerol O-acyltransferase 1 | | Dgat1 | ENSMUSG00000022555 | 0.61 | 6.94E-04 | 1.22E-01 |
| cyclin-dependent kinase 6 | | Cdk6 | ENSMUSG00000040274 | 0.29 | 7.23E-04 | 1.22E-01 |
| ATPase, Na+/K+ transporting, alpha 3 polypeptide | | Atp1a3 | ENSMUSG00000040907 | -0.25 | 7.24E-04 | 1.22E-01 |
| regulator of chromosome condensation 2 | | Rcc2 | ENSMUSG00000040945 | 0.35 | 7.26E-04 | 1.22E-01 |
| solute carrier family 39 (metal ion transporter), member 11 | | Slc39a11 | ENSMUSG00000041654 | 0.67 | 7.33E-04 | 1.22E-01 |
| mitogen-activated protein kinase 11 | | Mapk11 | ENSMUSG00000053137 | -0.27 | 7.34E-04 | 1.22E-01 |
| Gm12689 | |  | ENSMUSG00000070891 | 0.13 | 7.45E-04 | 1.22E-01 |
| small nucleolar RNA host gene 20 | | Snhg20 | ENSMUSG00000086859 | 0.22 | 7.47E-04 | 1.22E-01 |
| deleted in lymphocytic leukemia, 2 | | Dleu2 | ENSMUSG00000097589 | 0.17 | 7.48E-04 | 1.22E-01 |
| Gm2830, pseudogene | |  | ENSMUSG00000086567 | 0.53 | 7.63E-04 | 1.23E-01 |
| immunoglobulin kappa chain variable 4-91 | | Igkv4-91 | ENSMUSG00000076532 | -1.04 | 7.78E-04 | 1.24E-01 |
| cyclin-dependent kinase 11B | | Cdk11b | ENSMUSG00000029062 | 0.24 | 8.06E-04 | 1.27E-01 |
| solute carrier family 7, member 6 opposite strand | | Slc7a6os | ENSMUSG00000033106 | 0.16 | 8.38E-04 | 1.31E-01 |
| troponin I, cardiac 3 | | Tnni3 | ENSMUSG00000035458 | 0.40 | 8.99E-04 | 1.39E-01 |
| uridine phosphorylase 2 | | Upp2 | ENSMUSG00000026839 | 0.49 | 9.79E-04 | 1.48E-01 |
| caspase recruitment domain family, member 9 | | Card9 | ENSMUSG00000026928 | 0.52 | 9.81E-04 | 1.48E-01 |
| midline 1 | | Mid1 | ENSMUSG00000035299 | 0.24 | 1.00E-03 | 1.50E-01 |
| erythrocyte membrane protein band 4.1 like 4a, opposite strand | | Epb4l4aos | ENSMUSG00000087590 | 0.29 | 1.09E-03 | 1.61E-01 |
| cholinergic receptor, nicotinic, beta polypeptide 1 (muscle) | | Chrnb1 | ENSMUSG00000041189 | 0.71 | 1.12E-03 | 1.62E-01 |
| Meis homeobox 3 | | Meis3 | ENSMUSG00000041420 | 0.36 | 1.12E-03 | 1.62E-01 |
| Gm49172, LncRNA | |  | ENSMUSG00000115049 | 0.50 | 1.13E-03 | 1.62E-01 |
| Fyn proto-oncogene | | Fyn | ENSMUSG00000019843 | 0.27 | 1.15E-03 | 1.63E-01 |
| tRNA methyltransferase 1 like | | Trmt1l | ENSMUSG00000053286 | 0.38 | 1.16E-03 | 1.63E-01 |
| beta-1,3-glucuronyltransferase 3 (glucuronosyltransferase I) | | B3gat3 | ENSMUSG00000071649 | 0.13 | 1.17E-03 | 1.63E-01 |
| methylcrotonoyl-Coenzyme A carboxylase 1 (alpha) | | Mccc1 | ENSMUSG00000027709 | -0.18 | 1.23E-03 | 1.68E-01 |
| N-acetyltransferase 8-like | | Nat8l | ENSMUSG00000048142 | 0.52 | 1.23E-03 | 1.68E-01 |
| cyclin-dependent kinase 10 | | Cdk10 | ENSMUSG00000033862 | 0.33 | 1.25E-03 | 1.69E-01 |
| deoxyribonuclease II alpha | | Dnase2a | ENSMUSG00000003812 | 0.40 | 1.30E-03 | 1.74E-01 |
| SPARC related modular calcium binding 1 | | Smoc1 | ENSMUSG00000021136 | 0.50 | 1.35E-03 | 1.74E-01 |
| interferon induced transmembrane protein 1 | | Ifitm1 | ENSMUSG00000025491 | 0.38 | 1.35E-03 | 1.74E-01 |
| secreted phosphoprotein 1 | | Spp1 | ENSMUSG00000029304 | -0.25 | 1.36E-03 | 1.74E-01 |
| protocadherin 17 | | Pcdh17 | ENSMUSG00000035566 | 0.96 | 1.37E-03 | 1.74E-01 |
| Rho guanine nucleotide exchange factor (GEF) 37 | | Arhgef37 | ENSMUSG00000045094 | 0.47 | 1.37E-03 | 1.74E-01 |
| Gm17066, LncRNA | |  | ENSMUSG00000091509 | 0.65 | 1.39E-03 | 1.74E-01 |
| Gm28438, pseudogene | |  | ENSMUSG00000101939 | 0.17 | 1.39E-03 | 1.74E-01 |
| coatomer protein complex, subunit gamma 2 | | Copg2 | ENSMUSG00000025607 | -0.18 | 1.42E-03 | 1.76E-01 |
| coenzyme Q9 | | Coq9 | ENSMUSG00000031782 | 0.19 | 1.45E-03 | 1.78E-01 |
| pygopus 2 | | Pygo2 | ENSMUSG00000047824 | -0.16 | 1.46E-03 | 1.78E-01 |
| RAB4B, member RAS oncogene family | | Rab4b | ENSMUSG00000053291 | 0.28 | 1.48E-03 | 1.78E-01 |
| small integral membrane protein 22 | | Smim22 | ENSMUSG00000096215 | 0.43 | 1.48E-03 | 1.78E-01 |
|  | |  | ENSMUSG00000100747 | -0.34 | 1.49E-03 | 1.78E-01 |
| WD repeat domain 91 | | Wdr91 | ENSMUSG00000058486 | 0.40 | 1.52E-03 | 1.79E-01 |
|  | |  | ENSMUSG00000029534 | -0.20 | 1.60E-03 | 1.87E-01 |
| transcription factor 7 like 2, T cell specific, HMG box | | Tcf7l2 | ENSMUSG00000024985 | 0.19 | 1.64E-03 | 1.90E-01 |
| 1700084E18Rik, LncRNA | |  | ENSMUSG00000117912 | 0.81 | 1.65E-03 | 1.90E-01 |
| RAB1B, member RAS oncogene family | | Rab1b | ENSMUSG00000024870 | -0.16 | 1.67E-03 | 1.91E-01 |
| dynein light chain Tctex-type 1C | | Dynlt1c | ENSMUSG00000000579 | -0.49 | 1.73E-03 | 1.94E-01 |
| sorting nexin 25 | | Snx25 | ENSMUSG00000038291 | 0.39 | 1.74E-03 | 1.94E-01 |
| FXYD domain-containing ion transport regulator 2 | | Fxyd2 | ENSMUSG00000059412 | -0.22 | 1.74E-03 | 1.94E-01 |
| insulin-like growth factor binding protein 5 | | Igfbp5 | ENSMUSG00000026185 | 0.18 | 1.79E-03 | 1.97E-01 |
| RNA binding motif protein 6 | | Rbm6 | ENSMUSG00000032582 | 0.82 | 1.80E-03 | 1.97E-01 |
| potassium channel, subfamily K, member 3 | | Kcnk3 | ENSMUSG00000049265 | 0.69 | 1.81E-03 | 1.97E-01 |

**Supplemental Table 2.** Gene Ontology (GO) pathways enriched with fluoxetine treatment (FDR ≤ 5%). Sorted most significant to least significant using *p*-value. Abbreviations: Sig, significant; UP, upregulated; DOWN, downregulated

| **Term** | **Total genes** | **Sig. genes** | **# UP** | **# DOWN** | ***p* – value** |
| --- | --- | --- | --- | --- | --- |
| GO:0045667---regulation of osteoblast differentiation | 13 | 5 | 4 | 1 | 1.32E-05 |
| GO:0048485---sympathetic nervous system development | 8 | 4 | 4 | 0 | 3.02E-05 |
| GO:1904045---cellular response to aldosterone | 4 | 3 | 0 | 3 | 7.06E-05 |
| GO:0042493---response to drug | 232 | 17 | 12 | 5 | 0.000137 |
| GO:0006883---cellular sodium ion homeostasis | 13 | 4 | 2 | 2 | 0.000278 |
| GO:0001503---ossification | 66 | 8 | 7 | 1 | 0.000319 |
| GO:0035336---long-chain fatty-acyl-CoA metabolic process | 6 | 3 | 2 | 1 | 0.00034 |
| GO:0035556---intracellular signal transduction | 323 | 20 | 15 | 5 | 0.00035 |
| GO:0005615---extracellular space | 714 | 34 | 22 | 12 | 0.00057 |
| GO:1902036---regulation of hematopoietic stem cell differentiation | 7 | 3 | 2 | 1 | 0.000583 |
| GO:0016747---transferase activity, transferring acyl groups other than amino-acyl groups | 17 | 4 | 4 | 0 | 0.000851 |
| GO:0035019---somatic stem cell population maintenance | 29 | 5 | 3 | 2 | 0.000861 |
| GO:0070652---HAUS complex | 8 | 3 | 2 | 1 | 0.000914 |
| GO:0006953---acute-phase response | 18 | 4 | 4 | 0 | 0.001072 |
| GO:0034364---high-density lipoprotein particle | 10 | 3 | 3 | 0 | 0.001883 |
| GO:0052547---regulation of peptidase activity | 10 | 3 | 3 | 0 | 0.001883 |
| GO:0005229---intracellular calcium activated chloride channel activity | 11 | 3 | 3 | 0 | 0.00254 |
| GO:0045670---regulation of osteoclast differentiation | 11 | 3 | 3 | 0 | 0.00254 |
| GO:0043278---response to morphine | 24 | 4 | 3 | 1 | 0.003288 |
| GO:0050684---regulation of mRNA processing | 12 | 3 | 3 | 0 | 0.003321 |
| GO:0071361---cellular response to ethanol | 12 | 3 | 2 | 1 | 0.003321 |
| GO:0016740---transferase activity | 1397 | 53 | 43 | 10 | 0.003749 |
| GO:0004693---cyclin-dependent protein serine/threonine kinase activity | 25 | 4 | 3 | 1 | 0.003834 |
| GO:0006640---monoacylglycerol biosynthetic process | 4 | 2 | 2 | 0 | 0.003992 |
| GO:0016936---galactoside binding | 4 | 2 | 1 | 1 | 0.003992 |
| GO:0030149---sphingolipid catabolic process | 4 | 2 | 1 | 1 | 0.003992 |
| GO:0038085---vascular endothelial growth factor binding | 4 | 2 | 1 | 1 | 0.003992 |
| GO:0097720---calcineurin-mediated signaling | 4 | 2 | 1 | 1 | 0.003992 |
| GO:0016018---cyclosporin A binding | 13 | 3 | 2 | 1 | 0.004233 |
| GO:0055089---fatty acid homeostasis | 13 | 3 | 3 | 0 | 0.004233 |
| GO:1990498---mitotic spindle microtubule | 13 | 3 | 2 | 1 | 0.004233 |
| GO:0008045---motor neuron axon guidance | 14 | 3 | 3 | 0 | 0.005284 |
| GO:0006811---ion transport | 324 | 17 | 13 | 4 | 0.0054 |
| GO:0010867---positive regulation of triglyceride biosynthetic process | 15 | 3 | 3 | 0 | 0.006478 |
| GO:0044297---cell body | 84 | 7 | 3 | 4 | 0.006537 |
| GO:0005955---calcineurin complex | 5 | 2 | 1 | 1 | 0.006538 |
| GO:0021785---branchiomotor neuron axon guidance | 5 | 2 | 2 | 0 | 0.006538 |
| GO:0033192---calmodulin-dependent protein phosphatase activity | 5 | 2 | 1 | 1 | 0.006538 |
| GO:0045656---negative regulation of monocyte differentiation | 5 | 2 | 1 | 1 | 0.006538 |
| GO:0051562---negative regulation of mitochondrial calcium ion concentration | 5 | 2 | 1 | 1 | 0.006538 |
| GO:0006520---cellular amino acid metabolic process | 16 | 3 | 3 | 0 | 0.007819 |
| GO:0022904---respiratory electron transport chain | 17 | 3 | 1 | 2 | 0.009312 |
| GO:0002318---myeloid progenitor cell differentiation | 6 | 2 | 1 | 1 | 0.009638 |
| GO:0019068---virion assembly | 6 | 2 | 0 | 2 | 0.009638 |
| GO:0030644---cellular chloride ion homeostasis | 6 | 2 | 2 | 0 | 0.009638 |
| GO:0035814---negative regulation of renal sodium excretion | 6 | 2 | 2 | 0 | 0.009638 |
| GO:0042587---glycogen granule | 6 | 2 | 1 | 1 | 0.009638 |
| GO:0043219---lateral loop | 6 | 2 | 2 | 0 | 0.009638 |
| GO:0045646---regulation of erythrocyte differentiation | 6 | 2 | 1 | 1 | 0.009638 |
| GO:0046050---UMP catabolic process | 6 | 2 | 2 | 0 | 0.009638 |
| GO:0050650---chondroitin sulfate proteoglycan biosynthetic process | 6 | 2 | 2 | 0 | 0.009638 |
| GO:2000491---positive regulation of hepatic stellate cell activation | 6 | 2 | 1 | 1 | 0.009638 |
| GO:0004715---non-membrane spanning protein tyrosine kinase activity | 33 | 4 | 4 | 0 | 0.010522 |
| GO:0045668---negative regulation of osteoblast differentiation | 33 | 4 | 2 | 2 | 0.010522 |
| GO:0006821---chloride transport | 34 | 4 | 4 | 0 | 0.011683 |
| GO:0030217---T cell differentiation | 34 | 4 | 4 | 0 | 0.011683 |
| GO:0008143---poly(A) binding | 19 | 3 | 3 | 0 | 0.012766 |
| GO:0003215---cardiac right ventricle morphogenesis | 7 | 2 | 2 | 0 | 0.01326 |
| GO:0003857---3-hydroxyacyl-CoA dehydrogenase activity | 7 | 2 | 1 | 1 | 0.01326 |
| GO:0006249---dCMP catabolic process | 7 | 2 | 2 | 0 | 0.01326 |
| GO:0008611---ether lipid biosynthetic process | 7 | 2 | 1 | 1 | 0.01326 |
| GO:0033689---negative regulation of osteoblast proliferation | 7 | 2 | 2 | 0 | 0.01326 |
| GO:0035634---response to stilbenoid | 7 | 2 | 2 | 0 | 0.01326 |
| GO:0042742---defense response to bacterium | 97 | 7 | 3 | 4 | 0.013915 |
| GO:0004672---protein kinase activity | 420 | 19 | 16 | 3 | 0.015316 |
| GO:0045087---innate immune response | 334 | 16 | 11 | 5 | 0.015419 |
| GO:0030514---negative regulation of BMP signaling pathway | 37 | 4 | 2 | 2 | 0.015649 |
| GO:0007169---transmembrane receptor protein tyrosine kinase signaling pathway | 77 | 6 | 6 | 0 | 0.015677 |
| GO:0032874---positive regulation of stress-activated MAPK cascade | 21 | 3 | 3 | 0 | 0.016859 |
| GO:0005890---sodium:potassium-exchanging ATPase complex | 8 | 2 | 2 | 0 | 0.017375 |
| GO:0005979---regulation of glycogen biosynthetic process | 8 | 2 | 1 | 1 | 0.017375 |
| GO:0009083---branched-chain amino acid catabolic process | 8 | 2 | 1 | 1 | 0.017375 |
| GO:0048557---embryonic digestive tract morphogenesis | 8 | 2 | 2 | 0 | 0.017375 |
| GO:0048680---positive regulation of axon regeneration | 8 | 2 | 0 | 2 | 0.017375 |
| GO:0051569---regulation of histone H3-K4 methylation | 8 | 2 | 2 | 0 | 0.017375 |
| GO:0070016---armadillo repeat domain binding | 8 | 2 | 2 | 0 | 0.017375 |
| GO:0070257---positive regulation of mucus secretion | 8 | 2 | 2 | 0 | 0.017375 |
| GO:1904645---response to amyloid-beta | 8 | 2 | 2 | 0 | 0.017375 |
| GO:0001664---G protein-coupled receptor binding | 39 | 4 | 4 | 0 | 0.018711 |
| GO:0007098---centrosome cycle | 39 | 4 | 3 | 1 | 0.018711 |
| GO:0016746---transferase activity, transferring acyl groups | 127 | 8 | 8 | 0 | 0.018878 |
| GO:0030282---bone mineralization | 22 | 3 | 2 | 1 | 0.019148 |
| GO:0046676---negative regulation of insulin secretion | 22 | 3 | 2 | 1 | 0.019148 |
| GO:0009952---anterior/posterior pattern specification | 59 | 5 | 4 | 1 | 0.019157 |
| GO:0004714---transmembrane receptor protein tyrosine kinase activity | 82 | 6 | 6 | 0 | 0.020785 |
| GO:0019731---antibacterial humoral response | 23 | 3 | 3 | 0 | 0.021601 |
| GO:0048872---homeostasis of number of cells | 23 | 3 | 3 | 0 | 0.021601 |
| GO:0019215---intermediate filament binding | 9 | 2 | 1 | 1 | 0.021955 |
| GO:0035455---response to interferon-alpha | 9 | 2 | 2 | 0 | 0.021955 |
| GO:0019838---growth factor binding | 41 | 4 | 4 | 0 | 0.02212 |
| GO:0106310---protein serine kinase activity | 292 | 14 | 11 | 3 | 0.02245 |
| GO:0106311---protein threonine kinase activity | 293 | 14 | 11 | 3 | 0.023037 |
| GO:0008157---protein phosphatase 1 binding | 24 | 3 | 2 | 1 | 0.024217 |
| GO:0036120---cellular response to platelet-derived growth factor stimulus | 24 | 3 | 2 | 1 | 0.024217 |
| GO:0008542---visual learning | 43 | 4 | 2 | 2 | 0.025884 |
| GO:0000164---protein phosphatase type 1 complex | 10 | 2 | 1 | 1 | 0.026973 |
| GO:0021675---nerve development | 10 | 2 | 2 | 0 | 0.026973 |
| GO:0030007---cellular potassium ion homeostasis | 10 | 2 | 1 | 1 | 0.026973 |
| GO:0032482---Rab protein signal transduction | 10 | 2 | 0 | 2 | 0.026973 |
| GO:0033173---calcineurin-NFAT signaling cascade | 10 | 2 | 1 | 1 | 0.026973 |
| GO:0033539---fatty acid beta-oxidation using acyl-CoA dehydrogenase | 10 | 2 | 1 | 1 | 0.026973 |
| GO:0046339---diacylglycerol metabolic process | 10 | 2 | 2 | 0 | 0.026973 |
| GO:0048667---cell morphogenesis involved in neuron differentiation | 10 | 2 | 1 | 1 | 0.026973 |
| GO:0048771---tissue remodeling | 10 | 2 | 2 | 0 | 0.026973 |
| GO:0055075---potassium ion homeostasis | 10 | 2 | 1 | 1 | 0.026973 |
| GO:0060394---negative regulation of pathway-restricted SMAD protein phosphorylation | 10 | 2 | 0 | 2 | 0.026973 |
| GO:0060416---response to growth hormone | 10 | 2 | 1 | 1 | 0.026973 |
| GO:0070679---inositol 1,4,5 trisphosphate binding | 10 | 2 | 2 | 0 | 0.026973 |
| GO:1902287---semaphorin-plexin signaling pathway involved in axon guidance | 10 | 2 | 2 | 0 | 0.026973 |
| GO:1990907---beta-catenin-TCF complex | 10 | 2 | 2 | 0 | 0.026973 |
| GO:0050775---positive regulation of dendrite morphogenesis | 25 | 3 | 2 | 1 | 0.026996 |
| GO:0004713---protein tyrosine kinase activity | 88 | 6 | 6 | 0 | 0.028274 |
| GO:0001501---skeletal system development | 66 | 5 | 5 | 0 | 0.029521 |
| GO:0003841---1-acylglycerol-3-phosphate O-acyltransferase activity | 11 | 2 | 2 | 0 | 0.032402 |
| GO:0004866---endopeptidase inhibitor activity | 11 | 2 | 2 | 0 | 0.032402 |
| GO:0008210---estrogen metabolic process | 11 | 2 | 2 | 0 | 0.032402 |
| GO:0008374---O-acyltransferase activity | 11 | 2 | 2 | 0 | 0.032402 |
| GO:0017154---semaphorin receptor activity | 11 | 2 | 2 | 0 | 0.032402 |
| GO:0034713---type I transforming growth factor beta receptor binding | 11 | 2 | 0 | 2 | 0.032402 |
| GO:0042610---CD8 receptor binding | 11 | 2 | 2 | 0 | 0.032402 |
| GO:0043596---nuclear replication fork | 11 | 2 | 2 | 0 | 0.032402 |
| GO:0048038---quinone binding | 11 | 2 | 1 | 1 | 0.032402 |
| GO:0048066---developmental pigmentation | 11 | 2 | 2 | 0 | 0.032402 |
| GO:0010811---positive regulation of cell-substrate adhesion | 27 | 3 | 2 | 1 | 0.033043 |
| GO:0021510---spinal cord development | 27 | 3 | 2 | 1 | 0.033043 |
| GO:0030511---positive regulation of transforming growth factor beta receptor signaling pathway | 27 | 3 | 3 | 0 | 0.033043 |
| GO:0016301---kinase activity | 519 | 21 | 17 | 4 | 0.033334 |
| GO:0006468---protein phosphorylation | 490 | 20 | 17 | 3 | 0.034486 |
| GO:0048469---cell maturation | 28 | 3 | 3 | 0 | 0.036308 |
| GO:0060079---excitatory postsynaptic potential | 28 | 3 | 3 | 0 | 0.036308 |
| GO:0009615---response to virus | 70 | 5 | 4 | 1 | 0.036772 |
| GO:0071944---cell periphery | 70 | 5 | 3 | 2 | 0.036772 |
| GO:0004674---protein serine/threonine kinase activity | 343 | 15 | 11 | 4 | 0.037708 |
| GO:0006024---glycosaminoglycan biosynthetic process | 12 | 2 | 2 | 0 | 0.038219 |
| GO:0008353---RNA polymerase II CTD heptapeptide repeat kinase activity | 12 | 2 | 1 | 1 | 0.038219 |
| GO:0016064---immunoglobulin mediated immune response | 12 | 2 | 2 | 0 | 0.038219 |
| GO:0034101---erythrocyte homeostasis | 12 | 2 | 1 | 1 | 0.038219 |
| GO:0045830---positive regulation of isotype switching | 12 | 2 | 2 | 0 | 0.038219 |
| GO:0048096---chromatin-mediated maintenance of transcription | 12 | 2 | 2 | 0 | 0.038219 |
| GO:1900016---negative regulation of cytokine production involved in inflammatory response | 12 | 2 | 1 | 1 | 0.038219 |
| GO:0016310---phosphorylation | 527 | 21 | 17 | 4 | 0.038315 |
| GO:0098794---postsynapse | 120 | 7 | 3 | 4 | 0.03895 |
| GO:0043565---sequence-specific DNA binding | 286 | 13 | 11 | 2 | 0.039325 |
| GO:0031397---negative regulation of protein ubiquitination | 49 | 4 | 3 | 1 | 0.039366 |
| GO:0071345---cellular response to cytokine stimulus | 29 | 3 | 3 | 0 | 0.039733 |
| GO:0005975---carbohydrate metabolic process | 148 | 8 | 5 | 3 | 0.041668 |
| GO:0009055---electron transfer activity | 30 | 3 | 2 | 1 | 0.043315 |
| GO:0051225---spindle assembly | 30 | 3 | 2 | 1 | 0.043315 |
| GO:0004707---MAP kinase activity | 13 | 2 | 2 | 0 | 0.044398 |
| GO:0032502---developmental process | 13 | 2 | 2 | 0 | 0.044398 |
| GO:0035641---locomotory exploration behavior | 13 | 2 | 2 | 0 | 0.044398 |
| GO:0042405---nuclear inclusion body | 13 | 2 | 1 | 1 | 0.044398 |
| GO:0051403---stress-activated MAPK cascade | 13 | 2 | 2 | 0 | 0.044398 |
| GO:0031012---extracellular matrix | 150 | 8 | 4 | 4 | 0.044508 |
| GO:0032580---Golgi cisterna membrane | 51 | 4 | 3 | 1 | 0.0446 |
| GO:0000165---MAPK cascade | 74 | 5 | 4 | 1 | 0.045028 |
| GO:0015630---microtubule cytoskeleton | 151 | 8 | 7 | 1 | 0.045975 |
| GO:0005881---cytoplasmic microtubule | 52 | 4 | 3 | 1 | 0.047356 |
| GO:0034644---cellular response to UV | 52 | 4 | 3 | 1 | 0.047356 |

**Supplemental Table 3.** Kyoto Encyclopedia of Genes and Genomes (KEGG) pathways enriched with fluoxetine treatment (FDR ≤ 5%). Sorted most significant to least significant using *p*-value. Abbreviations: Sig, significant; UP, upregulated; DOWN, downregulated

| **Term** | **Total genes** | **Sig. genes** | **# UP** | **# DOWN** | ***p* – value** |
| --- | --- | --- | --- | --- | --- |
| mmu04972 Pancreatic secretion | 57 | 7 | 5 | 2 | 0.000957 |
| mmu04728 Dopaminergic synapse | 95 | 9 | 6 | 3 | 0.001248 |
| mmu04658 Th1 and Th2 cell differentiation | 62 | 7 | 6 | 1 | 0.001585 |
| mmu00280 Valine, leucine and isoleucine degradation | 46 | 6 | 2 | 4 | 0.00162 |
| mmu04924 Renin secretion | 55 | 6 | 4 | 2 | 0.00408 |
| mmu04974 Protein digestion and absorption | 55 | 6 | 5 | 1 | 0.00408 |
| mmu04657 IL-17 signaling pathway | 59 | 6 | 5 | 1 | 0.005788 |
| mmu04380 Osteoclast differentiation | 100 | 8 | 6 | 2 | 0.006609 |
| mmu04022 cGMP-PKG signaling pathway | 123 | 9 | 5 | 4 | 0.007222 |
| mmu04960 Aldosterone-regulated sodium reabsorption | 31 | 4 | 2 | 2 | 0.010343 |
| mmu04724 Glutamatergic synapse | 68 | 6 | 2 | 4 | 0.011458 |
| mmu04261 Adrenergic signaling in cardiomyocytes | 111 | 8 | 6 | 2 | 0.012135 |
| mmu04720 Long-term potentiation | 53 | 5 | 2 | 3 | 0.01572 |
| mmu04270 Vascular smooth muscle contraction | 94 | 7 | 2 | 5 | 0.015743 |
| mmu04750 Inflammatory mediator regulation of TRP channels | 73 | 6 | 4 | 2 | 0.015912 |
| mmu04975 Fat digestion and absorption | 20 | 3 | 3 | 0 | 0.017341 |
| mmu04659 Th17 cell differentiation | 75 | 6 | 5 | 1 | 0.017989 |
| mmu04970 Salivary secretion | 55 | 5 | 3 | 2 | 0.018231 |
| mmu04611 Platelet activation | 97 | 7 | 5 | 2 | 0.018443 |
| mmu04114 Oocyte meiosis | 100 | 7 | 4 | 3 | 0.021453 |
| mmu04660 T cell receptor signaling pathway | 79 | 6 | 5 | 1 | 0.022688 |
| mmu04218 Cellular senescence | 150 | 9 | 6 | 3 | 0.024173 |
| mmu04625 C-type lectin receptor signaling pathway | 84 | 6 | 5 | 1 | 0.029644 |
| mmu04371 Apelin signaling pathway | 109 | 7 | 4 | 3 | 0.032504 |
| mmu00561 Glycerolipid metabolism | 44 | 4 | 3 | 1 | 0.033784 |
| mmu04927 Cortisol synthesis and secretion | 46 | 4 | 3 | 1 | 0.038922 |
| mmu04723 Retrograde endocannabinoid signaling | 91 | 6 | 4 | 2 | 0.041543 |
| mmu00640 Propanoate metabolism | 28 | 3 | 1 | 2 | 0.042389 |
| mmu04925 Aldosterone synthesis and secretion | 71 | 5 | 4 | 1 | 0.04799 |
| mmu00140 Steroid hormone biosynthesis | 13 | 2 | 2 | 0 | 0.049756 |

**Supplemental Table 4.** Medical Subject Headings (MeSH) pathways enriched with fluoxetine treatment (FDR ≤ 5%). Sorted most significant to least significant using *p*-value. Abbreviations: Sig, significant; UP, upregulated; DOWN, downregulated

| **Term** | **Total genes** | **Sig. genes** | **# UP** | **# DOWN** | ***p* – value** |
| --- | --- | --- | --- | --- | --- |
| D064428---Hyperpolarization-Activated Cyclic Nucleotide-Gated Channels | 138 | 15 | 9 | 6 | 4E-06 |
| D050494---Vesicular Acetylcholine Transport Proteins | 52 | 9 | 6 | 3 | 8E-06 |
| D005075---Biological Evolution | 975 | 49 | 35 | 14 | 1E-05 |
| D050496---Vesicular Glutamate Transport Proteins | 22 | 6 | 5 | 1 | 2E-05 |
| D039081---Integrin alpha5beta1 | 196 | 17 | 11 | 6 | 2E-05 |
| D054815---Cyclic Nucleotide-Gated Cation Channels | 151 | 14 | 8 | 6 | 5E-05 |
| D055758---SOX9 Transcription Factor | 648 | 35 | 25 | 10 | 5E-05 |
| D000779---Anesthetics, Local | 56 | 8 | 6 | 2 | 0.0001 |
| D019143---Evolution, Molecular | 2705 | 101 | 70 | 31 | 0.0001 |
| D005998---Glycine | 441 | 26 | 15 | 11 | 0.0001 |
| D012727---Sex Characteristics | 932 | 44 | 30 | 14 | 0.0001 |
| D000943---Antigens, Differentiation | 831 | 40 | 27 | 13 | 0.0002 |
| D002148---Calmodulin-Binding Proteins | 309 | 20 | 11 | 10 | 0.0002 |
| D060850---LIM-Homeodomain Proteins | 732 | 36 | 25 | 11 | 0.0003 |
| D036961---Matrix Attachment Region Binding Proteins | 136 | 12 | 10 | 2 | 0.0003 |
| D015222---Sodium Channels | 388 | 23 | 15 | 8 | 0.0003 |
| D003907---Dexamethasone | 769 | 37 | 27 | 11 | 0.0003 |
| D051101---Neurturin | 36 | 6 | 4 | 2 | 0.0003 |
| D064092---Calbindin 1 | 36 | 6 | 3 | 3 | 0.0003 |
| D064231---Nestin | 444 | 25 | 16 | 9 | 0.0003 |
| D014543---Uridine Phosphorylase | 6 | 3 | 3 | 0 | 0.0004 |
| D015342---DNA Probes | 1007 | 45 | 34 | 11 | 0.0004 |
| D008040---Genetic Linkage | 1906 | 74 | 52 | 23 | 0.0004 |
| D026941---Sodium Channel Blockers | 143 | 12 | 5 | 7 | 0.0004 |
| D024022---Fibrillar Collagens | 86 | 9 | 6 | 3 | 0.0005 |
| D012083---Renin | 231 | 16 | 13 | 3 | 0.0005 |
| D027981---Symporters | 327 | 20 | 12 | 8 | 0.0005 |
| D048429---Cell Size | 1018 | 45 | 33 | 12 | 0.0005 |
| D062085---RNA, Long Noncoding | 785 | 37 | 27 | 11 | 0.0005 |
| D001846---Bone Development | 704 | 34 | 22 | 12 | 0.0005 |
| D019334---Endothelin-3 | 40 | 6 | 4 | 2 | 0.0006 |
| D013755---Tetradecanoylphorbol Acetate | 882 | 40 | 29 | 11 | 0.0006 |
| D050809---POU Domain Factors | 360 | 21 | 14 | 7 | 0.0006 |
| D007797---Laminin | 797 | 37 | 24 | 13 | 0.0007 |
| D004921---NA | 263 | 17 | 12 | 5 | 0.0007 |
| D038081---NA | 828 | 38 | 27 | 11 | 0.0007 |
| D050816---Octamer Transcription Factor-6 | 57 | 7 | 5 | 2 | 0.0007 |
| D058578---Biglycan | 74 | 8 | 6 | 2 | 0.0008 |
| D009433---Neural Inhibition | 416 | 23 | 14 | 9 | 0.0008 |
| D054397---Receptors, CCR3 | 42 | 6 | 5 | 2 | 0.0008 |
| D013779---Tetrodotoxin | 175 | 13 | 8 | 5 | 0.0008 |
| D017362---Cyclic AMP Response Element-Binding Protein | 895 | 40 | 29 | 11 | 0.0008 |
| D007038---Hypotonic Solutions | 43 | 6 | 4 | 2 | 0.0009 |
| D019899---Paracrine Communication | 640 | 31 | 22 | 9 | 0.0009 |
| D044927---Electron-Transferring Flavoproteins | 8 | 3 | 1 | 2 | 0.0009 |
| D055396---NA | 272 | 17 | 8 | 9 | 0.001 |
| D060508---Wnt3 Protein | 248 | 16 | 13 | 3 | 0.001 |
| D005801---Genes, Homeobox | 643 | 31 | 26 | 6 | 0.001 |
| D039842---Neural Cell Adhesion Molecule L1 | 157 | 12 | 8 | 4 | 0.001 |
| D004164---Diphosphonates | 96 | 9 | 8 | 1 | 0.001 |
| D057126---Nuclear Receptor Subfamily 4, Group A, Member 2 | 202 | 14 | 9 | 5 | 0.001 |
| D009381---Neoplasms, Radiation-Induced | 137 | 11 | 8 | 4 | 0.0011 |
| D012045---Regulatory Sequences, Nucleic Acid | 1149 | 48 | 30 | 18 | 0.0011 |
| D017461---Receptors, Atrial Natriuretic Factor | 159 | 12 | 8 | 4 | 0.0011 |
| D002317---Cardiovascular Agents | 79 | 8 | 5 | 3 | 0.0012 |
| D030161---Avian Proteins | 302 | 18 | 13 | 5 | 0.0012 |
| D017288---Pain Threshold | 206 | 14 | 12 | 2 | 0.0012 |
| D013181---Cortical Spreading Depression | 31 | 5 | 5 | 0 | 0.0012 |
| D003094---Collagen | 1406 | 56 | 41 | 15 | 0.0013 |
| D020440---Gene Duplication | 407 | 22 | 19 | 3 | 0.0013 |
| D055755---SOXD Transcription Factors | 305 | 18 | 13 | 5 | 0.0014 |
| D007696---Kindling, Neurologic | 63 | 7 | 5 | 2 | 0.0014 |
| D005481---Flurothyl | 9 | 3 | 2 | 1 | 0.0014 |
| D050615---Sodium-Phosphate Cotransporter Proteins, Type IIc | 9 | 3 | 3 | 0 | 0.0014 |
| D018118---Chloride Channels | 331 | 19 | 12 | 7 | 0.0014 |
| D025802---Electric Capacitance | 32 | 5 | 4 | 1 | 0.0014 |
| D001285---Atropine | 64 | 7 | 5 | 2 | 0.0015 |
| D009414---Nerve Growth Factors | 747 | 34 | 22 | 13 | 0.0015 |
| D015971---Gene Expression Regulation, Enzymologic | 2737 | 96 | 63 | 34 | 0.0016 |
| D051898---NA | 102 | 9 | 6 | 3 | 0.0016 |
| D004039---Diet, Sodium-Restricted | 48 | 6 | 2 | 4 | 0.0016 |
| D018663---Adrenergic Agents | 48 | 6 | 5 | 1 | 0.0016 |
| D000305---Adrenal Cortex Hormones | 123 | 10 | 6 | 4 | 0.0016 |
| D038041---NA | 167 | 12 | 12 | 0 | 0.0017 |
| D049411---Utrophin | 84 | 8 | 4 | 4 | 0.0017 |
| D051096---Proto-Oncogene Proteins c-ret | 364 | 20 | 14 | 6 | 0.0018 |
| D062554---NAV1.5 Voltage-Gated Sodium Channel | 85 | 8 | 7 | 1 | 0.0019 |
| D017457---Receptors, Albumin | 10 | 3 | 3 | 0 | 0.0019 |
| D050480---Plasma Membrane Neurotransmitter Transport Proteins | 10 | 3 | 2 | 1 | 0.0019 |
| D058905---Purinergic Agents | 10 | 3 | 2 | 1 | 0.0019 |
| D011963---Receptors, GABA-A | 341 | 19 | 12 | 7 | 0.002 |
| D038681---Follistatin | 193 | 13 | 9 | 4 | 0.002 |
| D036141---Receptor, EphA7 | 50 | 6 | 3 | 3 | 0.002 |
| D011519---Proto-Oncogenes | 421 | 22 | 17 | 5 | 0.002 |
| D017419---Potassium, Dietary | 21 | 4 | 2 | 2 | 0.0021 |
| D012890---Sleep | 194 | 13 | 10 | 3 | 0.0021 |
| D005865---Gestational Age | 1375 | 54 | 37 | 17 | 0.0021 |
| D003345---NA | 195 | 13 | 8 | 5 | 0.0022 |
| D019653---Myeloablative Agonists | 35 | 5 | 3 | 2 | 0.0022 |
| D044502---Thymine DNA Glycosylase | 35 | 5 | 3 | 2 | 0.0022 |
| D051155---Wnt1 Protein | 397 | 21 | 13 | 8 | 0.0022 |
| D050818---Transcription Factor Brn-3 | 51 | 6 | 4 | 2 | 0.0022 |
| D007377---Interleukin-3 | 425 | 22 | 18 | 5 | 0.0023 |
| D004553---Electric Conductivity | 244 | 15 | 10 | 5 | 0.0023 |
| D027682---Cation Transport Proteins | 453 | 23 | 15 | 8 | 0.0023 |
| D019672---Genes, T-Cell Receptor | 129 | 10 | 3 | 7 | 0.0023 |
| D018927---Anti-Asthmatic Agents | 52 | 6 | 5 | 1 | 0.0025 |
| D047209---Eosinophil Peroxidase | 22 | 4 | 4 | 0 | 0.0025 |
| D058068---Endophenotypes | 22 | 4 | 4 | 0 | 0.0025 |
| D008894---Milk Proteins | 322 | 18 | 16 | 2 | 0.0025 |
| D000469---NA | 456 | 23 | 17 | 6 | 0.0025 |
| D018171---NA | 70 | 7 | 4 | 3 | 0.0025 |
| D018094---Receptors, Metabotropic Glutamate | 247 | 15 | 11 | 4 | 0.0025 |
| D000918---Antibody Specificity | 831 | 36 | 20 | 17 | 0.0026 |
| D051100---Glial Cell Line-Derived Neurotrophic Factor | 298 | 17 | 12 | 6 | 0.0026 |
| D051792---Basic Helix-Loop-Helix Transcription Factors | 1712 | 64 | 40 | 24 | 0.0027 |
| D013990---Tidal Volume | 37 | 5 | 3 | 2 | 0.0028 |
| D000222---Adaptation, Physiological | 688 | 31 | 18 | 14 | 0.0028 |
| D018721---Muscarinic Agonists | 91 | 8 | 6 | 2 | 0.0029 |
| D016236---Genes, fms | 23 | 4 | 2 | 2 | 0.0029 |
| D002897---Chromosomes, Human, Pair 7 | 354 | 19 | 9 | 10 | 0.003 |
| D014654---Vascular Patency | 54 | 6 | 4 | 2 | 0.003 |
| D013735---Testicular Hormones | 92 | 8 | 7 | 1 | 0.0031 |
| D016232---Endothelins | 92 | 8 | 5 | 3 | 0.0031 |
| D014018---Tissue Distribution | 4240 | 137 | 100 | 38 | 0.0031 |
| D008564---Membrane Potentials | 841 | 36 | 22 | 14 | 0.0031 |
| D001287---Attachment Sites, Microbiological | 38 | 5 | 3 | 2 | 0.0031 |
| D044943---Fatty Acid Desaturases | 113 | 9 | 7 | 2 | 0.0032 |
| D014178---Translocation, Genetic | 635 | 29 | 19 | 10 | 0.0032 |
| D020890---Neuregulin-1 | 304 | 17 | 10 | 7 | 0.0032 |
| D020932---Nerve Growth Factor | 521 | 25 | 14 | 12 | 0.0032 |
| D020780---Matrix Metalloproteinase 9 | 665 | 30 | 20 | 10 | 0.0033 |
| D000445---Aldehyde Oxidoreductases | 229 | 14 | 11 | 3 | 0.0033 |
| D047228---Angiotensin II Type 1 Receptor Blockers | 158 | 11 | 8 | 3 | 0.0033 |
| D050980---GATA Transcription Factors | 114 | 9 | 5 | 4 | 0.0033 |
| D017442---Histamine Agonists | 12 | 3 | 3 | 0 | 0.0034 |
| D006649---Histocompatibility Antigens | 182 | 12 | 10 | 2 | 0.0034 |
| D007473---Ion Channels | 697 | 31 | 22 | 9 | 0.0035 |
| D027182---Amino Acid Transport System y+ | 94 | 8 | 5 | 3 | 0.0035 |
| D051578---Proto-Oncogene Proteins c-fes | 39 | 5 | 4 | 1 | 0.0035 |
| D065608---Renal Reabsorption | 39 | 5 | 3 | 2 | 0.0035 |
| D019849---Sex Determination Processes | 360 | 19 | 14 | 5 | 0.0036 |
| D018343---Receptors, Adrenergic, beta-2 | 183 | 12 | 9 | 3 | 0.0036 |
| D004262---DNA Restriction Enzymes | 361 | 19 | 15 | 4 | 0.0037 |
| D009024---NA | 1095 | 44 | 31 | 14 | 0.0037 |
| D055395---Bone Morphogenetic Protein 1 | 75 | 7 | 3 | 4 | 0.0037 |
| D002795---Choline O-Acetyltransferase | 184 | 12 | 7 | 5 | 0.0038 |
| D024683---Potassium Channels, Tandem Pore Domain | 57 | 6 | 4 | 2 | 0.0039 |
| D002134---NA | 209 | 13 | 7 | 6 | 0.0039 |
| D001119---Arginase | 185 | 12 | 10 | 2 | 0.0039 |
| D006016---Glycolates | 25 | 4 | 3 | 1 | 0.004 |
| D053649---Receptors, Interleukin-5 | 25 | 4 | 4 | 0 | 0.004 |
| D002097---C-Reactive Protein | 364 | 19 | 15 | 4 | 0.004 |
| D010281---Parathyroid Hormone | 311 | 17 | 12 | 5 | 0.004 |
| D002888---Chromosomes, Human, Pair 19 | 446 | 22 | 17 | 5 | 0.004 |
| D002125---Calcium Gluconate | 4 | 2 | 2 | 0 | 0.0041 |
| D008729---Methoxamine | 4 | 2 | 2 | 0 | 0.0041 |
| D044183---Receptor, Parathyroid Hormone, Type 2 | 4 | 2 | 2 | 0 | 0.0041 |
| D015094---3-Hydroxyacyl CoA Dehydrogenases | 140 | 10 | 8 | 2 | 0.0042 |
| D044388---GTP-Binding Protein gamma Subunits | 118 | 9 | 6 | 3 | 0.0042 |
| D016559---Tacrolimus | 211 | 13 | 9 | 4 | 0.0043 |
| D010751---Phosphopyruvate Hydratase | 313 | 17 | 14 | 4 | 0.0043 |
| D059305---Diet, High-Fat | 1167 | 46 | 38 | 8 | 0.0043 |
| D002710---Chlorhexidine | 13 | 3 | 2 | 1 | 0.0044 |
| D062559---NAV1.8 Voltage-Gated Sodium Channel | 41 | 5 | 3 | 2 | 0.0044 |
| D012697---Serine Endopeptidases | 921 | 38 | 24 | 15 | 0.0045 |
| D044387---GTP-Binding Protein beta Subunits | 98 | 8 | 4 | 4 | 0.0045 |
| D053781---Transforming Growth Factor beta2 | 263 | 15 | 9 | 6 | 0.0046 |
| D019765---Chondroitin ABC Lyase | 26 | 4 | 2 | 2 | 0.0046 |
| D050861---Synaptotagmin II | 26 | 4 | 3 | 1 | 0.0046 |
| D051857---Otx Transcription Factors | 238 | 14 | 9 | 5 | 0.0046 |
| D018019---Receptors, Corticotropin-Releasing Hormone | 78 | 7 | 5 | 2 | 0.0046 |
| D011983---Receptors, Purinergic | 59 | 6 | 3 | 3 | 0.0047 |
| D020098---Natriuretic Peptide, C-Type | 59 | 6 | 5 | 1 | 0.0047 |
| D024041---Non-Fibrillar Collagens | 59 | 6 | 5 | 1 | 0.0047 |
| D064026---Calbindins | 290 | 16 | 12 | 4 | 0.0048 |
| D049108---Cell Enlargement | 166 | 11 | 8 | 3 | 0.0048 |
| D005697---NA | 143 | 10 | 7 | 3 | 0.0049 |
| D020933---Neurotrophin 3 | 143 | 10 | 7 | 3 | 0.0049 |
| D052004---Bone Morphogenetic Protein Receptors | 190 | 12 | 7 | 5 | 0.0049 |
| D008903---NA | 42 | 5 | 4 | 1 | 0.0049 |
| D019076---Transgenes | 1691 | 62 | 41 | 22 | 0.0049 |
| D007527---Isoenzymes | 2023 | 72 | 52 | 20 | 0.0049 |
| D018272---Porins | 79 | 7 | 6 | 2 | 0.005 |
| D019063---Tenascin | 266 | 15 | 6 | 9 | 0.0051 |
| D005346---Fibroblast Growth Factors | 1273 | 49 | 33 | 16 | 0.0051 |
| D013599---Systole | 144 | 10 | 8 | 2 | 0.0051 |
| D027341---Excitatory Amino Acid Transporter 1 | 100 | 8 | 5 | 3 | 0.0051 |
| D008098---Litter Size | 216 | 13 | 13 | 0 | 0.0052 |
| D004920---Erythropoiesis | 838 | 35 | 29 | 6 | 0.0053 |
| D006238---Haploidy | 168 | 11 | 9 | 2 | 0.0053 |
| D005746---Gastric Emptying | 192 | 12 | 10 | 2 | 0.0053 |
| D018028---Receptors, Neurotensin | 27 | 4 | 3 | 1 | 0.0053 |
| D050719---Crk-Associated Substrate Protein | 145 | 10 | 8 | 2 | 0.0054 |
| D013565---Sympatholytics | 43 | 5 | 3 | 2 | 0.0054 |
| D054832---Urocortins | 43 | 5 | 3 | 2 | 0.0054 |
| D004533---Egtazic Acid | 268 | 15 | 10 | 5 | 0.0054 |
| D060449---Wnt Signaling Pathway | 1026 | 41 | 26 | 15 | 0.0055 |
| D003241---Consanguinity | 146 | 10 | 5 | 5 | 0.0056 |
| D020864---Calcium Channels, N-Type | 146 | 10 | 6 | 4 | 0.0056 |
| D019485---Bone Morphogenetic Proteins | 1027 | 41 | 26 | 15 | 0.0056 |
| D009498---Neurotoxins | 322 | 17 | 11 | 7 | 0.0057 |
| D012150---Polymorphism, Restriction Fragment Length | 904 | 37 | 28 | 9 | 0.0057 |
| D003097---Collateral Circulation | 102 | 8 | 6 | 2 | 0.0058 |
| D043422---Carboxypeptidases A | 102 | 8 | 6 | 2 | 0.0058 |
| D000685---Serum Amyloid A Protein | 124 | 9 | 9 | 0 | 0.0058 |
| D015820---Cadherins | 1443 | 54 | 34 | 20 | 0.0058 |
| D050656---Transcription Factor AP-2 | 377 | 19 | 15 | 5 | 0.0058 |
| D016291---Dizocilpine Maleate | 147 | 10 | 4 | 6 | 0.0059 |
| D039481---CD11b Antigen | 461 | 22 | 19 | 3 | 0.0059 |
| D000423---Alcian Blue | 62 | 6 | 5 | 1 | 0.0059 |
| D002038---Bungarotoxins | 62 | 6 | 5 | 1 | 0.0059 |
| D002491---Central Nervous System Agents | 44 | 5 | 4 | 1 | 0.006 |
| D013744---Tetanus Toxin | 44 | 5 | 4 | 1 | 0.006 |
| D036225---Receptor, EphB1 | 44 | 5 | 4 | 1 | 0.006 |
| D051788---Myeloid-Lymphoid Leukemia Protein | 351 | 18 | 14 | 4 | 0.006 |
| D058286---Calcitonin Receptor-Like Protein | 28 | 4 | 3 | 1 | 0.0061 |
| D015221---Potassium Channels | 462 | 22 | 15 | 7 | 0.0061 |
| D052005---Bone Morphogenetic Protein Receptors, Type I | 272 | 15 | 11 | 4 | 0.0062 |
| D015698---Genomic Library | 667 | 29 | 20 | 9 | 0.0063 |
| D051550---I-kappa B Kinase | 697 | 30 | 21 | 10 | 0.0063 |
| D050796---STAT3 Transcription Factor | 1003 | 40 | 32 | 8 | 0.0064 |
| D059467---Transcriptome | 1945 | 69 | 52 | 17 | 0.0064 |
| D000880---Anthraquinones | 83 | 7 | 5 | 2 | 0.0065 |
| D042002---Chromatin Assembly and Disassembly | 789 | 33 | 25 | 8 | 0.0065 |
| D019870---1-Phosphatidylinositol 4-Kinase | 45 | 5 | 5 | 0 | 0.0066 |
| D008139---Loperamide | 15 | 3 | 2 | 1 | 0.0067 |
| D015104---3,4-Methylenedioxyamphetamine | 5 | 2 | 2 | 0 | 0.0067 |
| D044822---Biodiversity | 5 | 2 | 2 | 0 | 0.0067 |
| D058446---Ligand-Gated Ion Channels | 5 | 2 | 2 | 0 | 0.0067 |
| D051785---Smad Proteins | 553 | 25 | 20 | 5 | 0.0068 |
| D002809---Chondroitin Sulfates | 174 | 11 | 6 | 5 | 0.0068 |
| D010012---Osteogenesis | 1229 | 47 | 32 | 15 | 0.0068 |
| D049031---Sarcoglycans | 105 | 8 | 7 | 1 | 0.0069 |
| D008901---Mineralocorticoids | 29 | 4 | 2 | 2 | 0.0069 |
| D016571---Neural Networks, Computer | 29 | 4 | 3 | 1 | 0.0069 |
| D012982---Sodium, Dietary | 64 | 6 | 3 | 3 | 0.0069 |
| D050820---Transcription Factor Brn-3B | 84 | 7 | 4 | 3 | 0.0069 |
| D004357---Drug Synergism | 613 | 27 | 20 | 7 | 0.007 |
| D010507---Periodicity | 151 | 10 | 8 | 2 | 0.0071 |
| D015415---Biomarkers | 2669 | 90 | 60 | 30 | 0.0072 |
| D003602---Cytotoxicity, Immunologic | 527 | 24 | 20 | 4 | 0.0073 |
| D051097---Glial Cell Line-Derived Neurotrophic Factor Receptors | 152 | 10 | 7 | 3 | 0.0074 |
| D012143---Respiratory Physiological Phenomena | 65 | 6 | 4 | 2 | 0.0075 |
| D019521---Body Patterning | 1994 | 70 | 46 | 24 | 0.0077 |
| D040542---Mechanotransduction, Cellular | 472 | 22 | 15 | 7 | 0.0077 |
| D005919---Glomerular Filtration Rate | 177 | 11 | 9 | 2 | 0.0077 |
| D020459---Genes, rRNA | 30 | 4 | 4 | 0 | 0.0078 |
| D002117---Calcitriol | 253 | 14 | 11 | 3 | 0.0078 |
| D012879---Skin Physiological Phenomena | 228 | 13 | 10 | 3 | 0.008 |
| D009292---Narcotic Antagonists | 66 | 6 | 4 | 2 | 0.008 |
| D017473---Receptors, Opioid, kappa | 66 | 6 | 2 | 4 | 0.008 |
| D042942---3-Methyl-2-Oxobutanoate Dehydrogenase (Lipoamide) | 16 | 3 | 2 | 1 | 0.0081 |
| D054401---Receptors, CCR8 | 16 | 3 | 3 | 0 | 0.0081 |
| D055413---Growth Differentiation Factor 10 | 16 | 3 | 1 | 2 | 0.0081 |
| D062367---ortho-Aminobenzoates | 16 | 3 | 3 | 0 | 0.0081 |
| D030301---Activin Receptors, Type II | 154 | 10 | 7 | 3 | 0.0081 |
| D002772---Cholera Toxin | 229 | 13 | 9 | 4 | 0.0083 |
| D024642---Potassium Channels, Voltage-Gated | 204 | 12 | 7 | 5 | 0.0084 |
| D005938---Glucocorticoids | 652 | 28 | 19 | 9 | 0.0085 |
| D053444---Inhibitory Postsynaptic Potentials | 109 | 8 | 4 | 4 | 0.0085 |
| D007334---Insulin-Like Growth Factor I | 959 | 38 | 28 | 11 | 0.0086 |
| D016194---Receptors, N-Methyl-D-Aspartate | 653 | 28 | 16 | 12 | 0.0087 |
| D045930---Anabolic Agents | 48 | 5 | 3 | 2 | 0.0087 |
| D018161---Receptors, Mineralocorticoid | 110 | 8 | 6 | 2 | 0.009 |
| D044140---Receptor, Angiotensin, Type 1 | 232 | 13 | 9 | 4 | 0.0092 |
| D011941---Receptors, Adrenergic | 68 | 6 | 4 | 2 | 0.0093 |
| D016326---Extracellular Matrix Proteins | 1743 | 62 | 39 | 23 | 0.0094 |
| D014446---Tyrosine 3-Monooxygenase | 568 | 25 | 17 | 8 | 0.0094 |
| D018755---GABA Agonists | 49 | 5 | 4 | 1 | 0.0094 |
| D019940---Genes, bcl-1 | 49 | 5 | 3 | 2 | 0.0094 |
| D017981---Receptors, Neurotransmitter | 111 | 8 | 4 | 4 | 0.0095 |
| D060492---Low Density Lipoprotein Receptor-Related Protein-5 | 111 | 8 | 5 | 3 | 0.0095 |
| D053498---Sarcoplasmic Reticulum Calcium-Transporting ATPases | 259 | 14 | 9 | 5 | 0.0095 |
| D014373---Tuberculin | 17 | 3 | 3 | 0 | 0.0096 |
| D015256---Coenzyme A-Transferases | 17 | 3 | 2 | 1 | 0.0096 |
| D018815---Genes, erbB | 17 | 3 | 3 | 0 | 0.0096 |
| D009118---Muscimol | 32 | 4 | 2 | 2 | 0.0098 |
| D018682---GABA Agents | 32 | 4 | 2 | 2 | 0.0098 |
| D048491---Proto-Oncogene Proteins A-raf | 32 | 4 | 2 | 2 | 0.0098 |
| D053661---Interleukin-13 Receptor alpha1 Subunit | 32 | 4 | 4 | 0 | 0.0098 |
| D006025---Glycosaminoglycans | 260 | 14 | 9 | 5 | 0.0098 |
| D015198---Designer Drugs | 6 | 2 | 1 | 1 | 0.0098 |
| D038341---Glycogen Synthase Kinases | 69 | 6 | 5 | 1 | 0.0099 |
| D004167---Diphtheria Toxin | 135 | 9 | 6 | 3 | 0.01 |
| D018079---Receptors, GABA | 112 | 8 | 5 | 3 | 0.01 |
| D039943---Neuropilin-2 | 112 | 8 | 5 | 3 | 0.01 |
| D017923---Sodium-Hydrogen Exchangers | 261 | 14 | 10 | 4 | 0.0101 |
| D014161---Transduction, Genetic | 876 | 35 | 27 | 8 | 0.0102 |
| D004231---Diuresis | 50 | 5 | 4 | 1 | 0.0103 |
| D008124---Locomotion | 427 | 20 | 15 | 5 | 0.0103 |
| D020778---Matrix Metalloproteinase 2 | 543 | 24 | 16 | 8 | 0.0103 |
| D018691---Excitatory Amino Acid Antagonists | 343 | 17 | 12 | 5 | 0.0104 |
| D000242---Cyclic AMP | 1226 | 46 | 30 | 16 | 0.0104 |
| D055418---Bone Morphogenetic Protein 6 | 91 | 7 | 4 | 3 | 0.0106 |
| D018080---Receptors, GABA-B | 70 | 6 | 4 | 2 | 0.0106 |
| D046352---Freezing Reaction, Cataleptic | 70 | 6 | 4 | 2 | 0.0106 |
| D063185---Epigenetic Repression | 70 | 6 | 5 | 1 | 0.0106 |
| D018800---Thy-1 Antigens | 161 | 10 | 9 | 1 | 0.0109 |
| D058477---Receptors, Purinergic P2X3 | 33 | 4 | 4 | 0 | 0.0109 |
| D003170---Complement Pathway, Alternative | 51 | 5 | 4 | 1 | 0.0111 |
| D011415---Complement Factor B | 51 | 5 | 4 | 1 | 0.0111 |
| D011493---Protein Kinase C | 1361 | 50 | 34 | 17 | 0.0111 |
| D018092---Receptors, Kainic Acid | 92 | 7 | 5 | 2 | 0.0112 |
| D020747---Calcium Channels, T-Type | 92 | 7 | 3 | 4 | 0.0112 |
| D022702---Receptors, Adrenergic, beta-3 | 92 | 7 | 7 | 0 | 0.0112 |
| D051524---Fibroblast Growth Factor 8 | 346 | 17 | 8 | 9 | 0.0112 |
| D051784---Aryl Hydrocarbon Receptor Nuclear Translocator | 138 | 9 | 6 | 3 | 0.0114 |
| D051745---Protein Kinase C-delta | 319 | 16 | 13 | 3 | 0.0114 |
| D018125---Receptors, Transforming Growth Factor beta | 759 | 31 | 19 | 12 | 0.0114 |
| D006609---High Mobility Group Proteins | 790 | 32 | 19 | 13 | 0.0115 |
| D009812---Odorants | 115 | 8 | 6 | 2 | 0.0116 |
| D011985---Receptors, Serotonin | 115 | 8 | 6 | 2 | 0.0116 |
| D039942---Neuropilin-1 | 239 | 13 | 7 | 6 | 0.0116 |
| D051153---Wnt Proteins | 1430 | 52 | 34 | 18 | 0.0117 |
| D009043---Motor Activity | 1267 | 47 | 33 | 15 | 0.0117 |
| D056284---Histone Deacetylase 1 | 320 | 16 | 11 | 5 | 0.0117 |
| D013936---Thymidine | 266 | 14 | 10 | 4 | 0.0118 |
| D004299---Dopamine beta-Hydroxylase | 93 | 7 | 4 | 3 | 0.0119 |
| D019891---NA | 93 | 7 | 7 | 0 | 0.0119 |
| D017450---Receptors, Opioid, mu | 139 | 9 | 4 | 5 | 0.0119 |
| D005799---Genes, Dominant | 1076 | 41 | 29 | 12 | 0.012 |
| D017855---Transferases (Other Substituted Phosphate Groups) | 52 | 5 | 4 | 1 | 0.0121 |
| D036123---Receptor, EphA5 | 52 | 5 | 4 | 1 | 0.0121 |
| D050599---Vesicular Glutamate Transport Protein 2 | 72 | 6 | 3 | 3 | 0.0121 |
| D007130---Immunoglobulin Idiotypes | 34 | 4 | 1 | 3 | 0.0121 |
| D008912---Minor Histocompatibility Loci | 34 | 4 | 3 | 1 | 0.0121 |
| D009612---Nicotinamide-Nucleotide Adenylyltransferase | 34 | 4 | 2 | 2 | 0.0121 |
| D012604---Scorpion Venoms | 34 | 4 | 2 | 2 | 0.0121 |
| D019948---Receptors, Interleukin-4 | 116 | 8 | 8 | 0 | 0.0122 |
| D055760---SOXF Transcription Factors | 164 | 10 | 7 | 3 | 0.0123 |
| D000949---Histocompatibility Antigens Class II | 522 | 23 | 20 | 3 | 0.0123 |
| D009030---NA | 140 | 9 | 6 | 3 | 0.0124 |
| D001663---Bilirubin | 94 | 7 | 6 | 1 | 0.0125 |
| D054737---Calcium-Calmodulin-Dependent Protein Kinase Kinase | 94 | 7 | 5 | 2 | 0.0125 |
| D020033---Protein Isoforms | 3571 | 114 | 80 | 35 | 0.0126 |
| D002843---Chromatin | 1869 | 65 | 46 | 20 | 0.0126 |
| D002880---Chromosomes, Human, Pair 11 | 436 | 20 | 16 | 4 | 0.0127 |
| D016384---Consensus Sequence | 922 | 36 | 29 | 8 | 0.0129 |
| D050485---GABA Plasma Membrane Transport Proteins | 73 | 6 | 4 | 2 | 0.0129 |
| D011509---Proteoglycans | 891 | 35 | 21 | 14 | 0.013 |
| D051738---Origin Recognition Complex | 53 | 5 | 2 | 3 | 0.013 |
| D064030---S100 Calcium Binding Protein G | 324 | 16 | 12 | 4 | 0.0131 |
| D001947---Breeding | 352 | 17 | 12 | 5 | 0.0131 |
| D019748---Carbon-Carbon Double Bond Isomerases | 19 | 3 | 3 | 0 | 0.0132 |
| D027362---Organic Anion Transport Protein 1 | 19 | 3 | 3 | 0 | 0.0132 |
| D049993---Sodium Chloride Symporter Inhibitors | 19 | 3 | 1 | 2 | 0.0132 |
| D060171---Uroplakin III | 19 | 3 | 2 | 1 | 0.0132 |
| D009088---NA | 95 | 7 | 7 | 0 | 0.0132 |
| D007004---Hypoglycemic Agents | 496 | 22 | 16 | 6 | 0.0133 |
| D011940---Receptor Aggregation | 166 | 10 | 7 | 3 | 0.0133 |
| D014882---Water-Electrolyte Balance | 166 | 10 | 7 | 3 | 0.0133 |
| D002135---Calcium-Binding Proteins | 1807 | 63 | 40 | 23 | 0.0134 |
| D066247---Receptor, ErbB-4 | 118 | 8 | 6 | 2 | 0.0134 |
| D000102---Acetyl-CoA C-Acyltransferase | 35 | 4 | 4 | 0 | 0.0134 |
| D044302---Receptor, Serotonin, 5-HT1B | 35 | 4 | 2 | 2 | 0.0134 |
| D046353---Immobility Response, Tonic | 35 | 4 | 4 | 0 | 0.0134 |
| D018809---Proliferating Cell Nuclear Antigen | 586 | 25 | 14 | 11 | 0.0135 |
| D002859---Chromium Isotopes | 7 | 2 | 2 | 0 | 0.0135 |
| D010480---Perhexiline | 7 | 2 | 2 | 0 | 0.0135 |
| D011774---Pyrvinium Compounds | 7 | 2 | 2 | 0 | 0.0135 |
| D010770---Phosphotransferases | 410 | 19 | 12 | 7 | 0.0136 |
| D057094---Nuclear Receptor Subfamily 1, Group F, Member 1 | 142 | 9 | 9 | 0 | 0.0136 |
| D014529---Uridine | 74 | 6 | 5 | 1 | 0.0137 |
| D013912---Threonine | 587 | 25 | 17 | 8 | 0.0138 |
| D002896---Chromosomes, Human, Pair 6 | 326 | 16 | 12 | 5 | 0.0138 |
| D051525---Fibroblast Growth Factor 9 | 167 | 10 | 9 | 1 | 0.0138 |
| D003292---Convulsants | 96 | 7 | 4 | 3 | 0.014 |
| D017466---Receptors, Endothelin | 96 | 7 | 6 | 1 | 0.014 |
| D051937---Dopamine and cAMP-Regulated Phosphoprotein 32 | 96 | 7 | 5 | 2 | 0.014 |
| D053523---Amelogenin | 96 | 7 | 6 | 1 | 0.014 |
| D000661---Amphetamine | 119 | 8 | 5 | 3 | 0.014 |
| D000978---Antiparkinson Agents | 54 | 5 | 2 | 3 | 0.0141 |
| D013148---Spironolactone | 54 | 5 | 2 | 3 | 0.0141 |
| D018971---Insulin-Like Growth Factor Binding Protein 2 | 54 | 5 | 4 | 1 | 0.0141 |
| D050506---Mutant Chimeric Proteins | 54 | 5 | 2 | 3 | 0.0141 |
| D053553---Keratin-6 | 54 | 5 | 4 | 1 | 0.0141 |
| D012091---Repetitive Sequences, Nucleic Acid | 897 | 35 | 24 | 11 | 0.0143 |
| D000804---Angiotensin II | 680 | 28 | 20 | 8 | 0.0143 |
| D016229---Amyloid beta-Peptides | 680 | 28 | 21 | 7 | 0.0143 |
| D008024---NA | 1480 | 53 | 39 | 15 | 0.0144 |
| D016271---Proto-Oncogene Proteins c-myc | 1057 | 40 | 29 | 12 | 0.0145 |
| D012965---Sodium Chloride | 273 | 14 | 11 | 3 | 0.0146 |
| D013449---Sulfonamides | 560 | 24 | 16 | 9 | 0.0146 |
| D006982---Hypertonic Solutions | 75 | 6 | 4 | 2 | 0.0146 |
| D019793---Fluorescein | 75 | 6 | 4 | 2 | 0.0146 |
| D016654---Genes, RAG-1 | 97 | 7 | 6 | 1 | 0.0147 |
| D018734---Genes, erbB-2 | 144 | 9 | 7 | 2 | 0.0148 |
| D008982---Molybdenum | 36 | 4 | 2 | 2 | 0.0148 |
| D020410---Activated-Leukocyte Cell Adhesion Molecule | 36 | 4 | 3 | 1 | 0.0148 |
| D002113---Calcification, Physiologic | 472 | 21 | 16 | 5 | 0.0149 |
| D019938---Cyclin D1 | 744 | 30 | 22 | 8 | 0.015 |
| D054338---Cell Transdifferentiation | 386 | 18 | 12 | 6 | 0.0151 |
| D003810---Dentinogenesis | 55 | 5 | 4 | 1 | 0.0151 |
| D008911---Minocycline | 55 | 5 | 3 | 2 | 0.0151 |
| D015345---Oligonucleotide Probes | 532 | 23 | 17 | 6 | 0.0151 |
| D001514---Bee Venoms | 20 | 3 | 3 | 0 | 0.0152 |
| D011659---Pulmonary Gas Exchange | 20 | 3 | 2 | 1 | 0.0152 |
| D049994---Sodium Potassium Chloride Symporter Inhibitors | 20 | 3 | 2 | 1 | 0.0152 |
| D056511---Metallochaperones | 20 | 3 | 2 | 1 | 0.0152 |
| D058261---Receptor Activity-Modifying Proteins | 20 | 3 | 2 | 1 | 0.0152 |
| D016695---Glycosyltransferases | 221 | 12 | 8 | 4 | 0.0152 |
| D000262---Adenylyl Cyclases | 330 | 16 | 9 | 7 | 0.0153 |
| D010972---Platelet Activating Factor | 145 | 9 | 7 | 2 | 0.0154 |
| D058305---Sorting Nexins | 170 | 10 | 8 | 2 | 0.0155 |
| D001640---Bicuculline | 98 | 7 | 4 | 3 | 0.0155 |
| D054304---Anti-Mullerian Hormone | 98 | 7 | 6 | 1 | 0.0155 |
| D011480---Protease Inhibitors | 445 | 20 | 11 | 9 | 0.0156 |
| D020127---Recovery of Function | 416 | 19 | 14 | 5 | 0.0156 |
| D011508---Chondroitin Sulfate Proteoglycans | 222 | 12 | 5 | 7 | 0.0157 |
| D006410---Hematopoiesis | 1356 | 49 | 37 | 13 | 0.0159 |
| D004298---Dopamine | 779 | 31 | 21 | 10 | 0.016 |
| D000814---Aniline Compounds | 171 | 10 | 7 | 3 | 0.0161 |
| D008078---Cholesterol, LDL | 171 | 10 | 8 | 2 | 0.0161 |
| D003746---Dental Enamel Proteins | 122 | 8 | 7 | 1 | 0.0161 |
| D024002---HMGB Proteins | 223 | 12 | 8 | 4 | 0.0162 |
| D045702---Proprotein Convertase 5 | 37 | 4 | 2 | 2 | 0.0162 |
| D058470---Receptors, Purinergic P2Y | 37 | 4 | 3 | 1 | 0.0162 |
| D019386---Alendronate | 56 | 5 | 5 | 0 | 0.0163 |
| D016514---Genes, Neurofibromatosis 1 | 77 | 6 | 2 | 4 | 0.0165 |
| D020880---Neuregulins | 77 | 6 | 4 | 2 | 0.0165 |
| D037483---Galectin 1 | 77 | 6 | 3 | 3 | 0.0165 |
| D008262---Macrophage Activation | 812 | 32 | 24 | 8 | 0.0165 |
| D018690---Excitatory Amino Acid Agonists | 305 | 15 | 9 | 6 | 0.0165 |
| D019898---Autocrine Communication | 567 | 24 | 17 | 8 | 0.0167 |
| D002453---Cell Cycle | 2967 | 96 | 69 | 28 | 0.0168 |
| D004742---Enhancer Elements, Genetic | 1394 | 50 | 37 | 13 | 0.0169 |
| D005809---Genes, Regulator | 598 | 25 | 18 | 8 | 0.017 |
| D011954---Receptors, Dopamine | 100 | 7 | 4 | 3 | 0.0172 |
| D051300---Wiskott-Aldrich Syndrome Protein Family | 100 | 7 | 5 | 2 | 0.0172 |
| D004347---Drug Interactions | 509 | 22 | 16 | 6 | 0.0174 |
| D002810---Chondroitinases and Chondroitin Lyases | 21 | 3 | 2 | 1 | 0.0174 |
| D043885---Receptor, Cannabinoid, CB2 | 57 | 5 | 5 | 0 | 0.0175 |
| D007506---Iron-Sulfur Proteins | 78 | 6 | 4 | 2 | 0.0175 |
| D055427---Growth Differentiation Factor 2 | 78 | 6 | 3 | 3 | 0.0175 |
| D000917---Antibody Formation | 510 | 22 | 17 | 5 | 0.0177 |
| D003185---Complement C8 | 8 | 2 | 1 | 1 | 0.0177 |
| D007550---Isothiuronium | 8 | 2 | 2 | 0 | 0.0177 |
| D011999---Recruitment, Neurophysiological | 8 | 2 | 1 | 1 | 0.0177 |
| D012026---Reflex, Stretch | 8 | 2 | 2 | 0 | 0.0177 |
| D020109---Devazepide | 8 | 2 | 2 | 0 | 0.0177 |
| D020736---Ribonucleoproteins, Small Cytoplasmic | 8 | 2 | 1 | 1 | 0.0177 |
| D018075---RNA, Complementary | 280 | 14 | 9 | 5 | 0.0177 |
| D020907---Calcium Channels, Q-Type | 38 | 4 | 2 | 2 | 0.0178 |
| D004549---Elastin | 226 | 12 | 9 | 3 | 0.0178 |
| D016718---Arachidonic Acid | 253 | 13 | 10 | 3 | 0.0179 |
| D012730---Sex Chromosomes | 174 | 10 | 8 | 2 | 0.0179 |
| D051523---Fibroblast Growth Factor 7 | 174 | 10 | 6 | 4 | 0.0179 |
| D014644---Genetic Variation | 1565 | 55 | 42 | 13 | 0.018 |
| D048054---Mitogen-Activated Protein Kinase 7 | 149 | 9 | 7 | 3 | 0.0181 |
| D037181---Lectins, C-Type | 632 | 26 | 21 | 5 | 0.0181 |
| D016202---N-Methylaspartate | 309 | 15 | 10 | 5 | 0.0184 |
| D013947---Thymosin | 125 | 8 | 5 | 3 | 0.0184 |
| D010713---Phosphatidylcholines | 254 | 13 | 12 | 1 | 0.0184 |
| D037721---Pulmonary Surfactant-Associated Protein C | 79 | 6 | 3 | 3 | 0.0185 |
| D055757---SOXE Transcription Factors | 366 | 17 | 13 | 4 | 0.0186 |
| D051418---Focal Adhesion Kinase 2 | 175 | 10 | 9 | 2 | 0.0186 |
| D058787---GABA-A Receptor Antagonists | 58 | 5 | 3 | 2 | 0.0187 |
| D059016---Tumor Microenvironment | 483 | 21 | 19 | 2 | 0.0187 |
| D017239---Paclitaxel | 282 | 14 | 10 | 5 | 0.0188 |
| D047392---beta-Cyclodextrins | 150 | 9 | 5 | 4 | 0.0188 |
| D000200---Action Potentials | 665 | 27 | 19 | 8 | 0.0189 |
| D051902---Smad5 Protein | 228 | 12 | 8 | 4 | 0.019 |
| D016329---Sp1 Transcription Factor | 604 | 25 | 17 | 8 | 0.019 |
| D011498---Protein Precursors | 821 | 32 | 25 | 8 | 0.019 |
| D051741---Kruppel-Like Transcription Factors | 1437 | 51 | 38 | 13 | 0.0191 |
| D017468---Receptors, Fibroblast Growth Factor | 425 | 19 | 13 | 6 | 0.0191 |
| D051176---beta Catenin | 1538 | 54 | 35 | 19 | 0.0193 |
| D055419---Bone Morphogenetic Protein 7 | 311 | 15 | 9 | 6 | 0.0193 |
| D013741---3-Oxo-5-alpha-Steroid 4-Dehydrogenase | 39 | 4 | 2 | 2 | 0.0194 |
| D019054---Evoked Potentials, Motor | 39 | 4 | 4 | 0 | 0.0194 |
| D020865---Calcium Channels, P-Type | 39 | 4 | 2 | 2 | 0.0194 |
| D027042---Chromosomes, Artificial, P1 Bacteriophage | 39 | 4 | 4 | 0 | 0.0194 |
| D062565---Acid Sensing Ion Channels | 39 | 4 | 3 | 1 | 0.0194 |
| D051959---ELAV Proteins | 151 | 9 | 4 | 5 | 0.0195 |
| D014998---Y Chromosome | 229 | 12 | 7 | 5 | 0.0196 |
| D060528---Wnt4 Protein | 229 | 12 | 9 | 3 | 0.0196 |
| D020648---Peptide Elongation Factor 1 | 80 | 6 | 5 | 1 | 0.0196 |
| D050657---Core Binding Factor alpha Subunits | 80 | 6 | 6 | 0 | 0.0196 |
| D001609---Beta-Globulins | 22 | 3 | 3 | 0 | 0.0197 |
| D016274---Oncogene Protein p55(v-myc) | 22 | 3 | 3 | 0 | 0.0197 |
| D044022---Receptor, Endothelin A | 103 | 7 | 5 | 2 | 0.0199 |
| D012084---Renin-Angiotensin System | 203 | 11 | 7 | 4 | 0.0199 |
| D004777---Environment | 177 | 10 | 7 | 3 | 0.02 |
| D016704---Synapsins | 177 | 10 | 5 | 5 | 0.02 |
| D016212---Transforming Growth Factor beta | 1946 | 66 | 46 | 20 | 0.02 |
| D001616---beta-Galactosidase | 1541 | 54 | 38 | 17 | 0.02 |
| D013759---Dronabinol | 59 | 5 | 3 | 2 | 0.02 |
| D005341---Fibrinolysin | 127 | 8 | 5 | 3 | 0.02 |
| D044385---GTP-Binding Protein alpha Subunits | 127 | 8 | 5 | 3 | 0.02 |
| D054732---Calcium-Calmodulin-Dependent Protein Kinase Type 2 | 669 | 27 | 17 | 10 | 0.0202 |
| D011505---Protein-Tyrosine Kinases | 1377 | 49 | 37 | 13 | 0.0205 |
| D055513---Connective Tissue Growth Factor | 258 | 13 | 7 | 6 | 0.0207 |
| D051496---Receptor, Fibroblast Growth Factor, Type 1 | 342 | 16 | 11 | 5 | 0.0207 |
| D050916---TRPV Cation Channels | 400 | 18 | 11 | 7 | 0.0209 |
| D053668---Syndecan-1 | 128 | 8 | 4 | 4 | 0.0209 |
| D004258---DNA Polymerase III | 40 | 4 | 4 | 0 | 0.0211 |
| D014305---Triose-Phosphate Isomerase | 40 | 4 | 4 | 0 | 0.0211 |
| D051040---Vesicle-Associated Membrane Protein 3 | 40 | 4 | 4 | 0 | 0.0211 |
| D017868---Cyclic AMP-Dependent Protein Kinases | 1020 | 38 | 24 | 14 | 0.0213 |
| D048670---MAP Kinase Kinase 4 | 580 | 24 | 20 | 4 | 0.0213 |
| D007743---Labor, Obstetric | 60 | 5 | 3 | 2 | 0.0214 |
| D018683---Excitatory Amino Acid Agents | 60 | 5 | 2 | 3 | 0.0214 |
| D021984---Cyclophilin A | 60 | 5 | 3 | 2 | 0.0214 |
| D048768---MAP Kinase Kinase Kinase 4 | 60 | 5 | 4 | 1 | 0.0214 |
| D051656---KCNQ Potassium Channels | 60 | 5 | 3 | 2 | 0.0214 |
| D055504---Insulin Receptor Substrate Proteins | 401 | 18 | 16 | 2 | 0.0214 |
| D014746---NA | 232 | 12 | 8 | 4 | 0.0214 |
| D051957---MSX1 Transcription Factor | 232 | 12 | 8 | 4 | 0.0214 |
| D003576---Electron Transport Complex IV | 315 | 15 | 7 | 8 | 0.0214 |
| D050992---GATA5 Transcription Factor | 82 | 6 | 4 | 2 | 0.0218 |
| D053818---Chymases | 82 | 6 | 5 | 1 | 0.0218 |
| D052243---Resistin | 105 | 7 | 6 | 1 | 0.0218 |
| D016368---Frameshift Mutation | 402 | 18 | 12 | 6 | 0.0218 |
| D007958---Leukocyte Count | 316 | 15 | 14 | 1 | 0.022 |
| D026901---Membrane Transport Proteins | 926 | 35 | 23 | 12 | 0.022 |
| D051546---Cyclooxygenase 2 | 582 | 24 | 19 | 5 | 0.0221 |
| D000961---Antilymphocyte Serum | 23 | 3 | 2 | 1 | 0.0222 |
| D018271---Signal Recognition Particle | 23 | 3 | 2 | 1 | 0.0222 |
| D049408---Luminescent Agents | 23 | 3 | 2 | 1 | 0.0222 |
| D020796---Receptor, Platelet-Derived Growth Factor alpha | 345 | 16 | 12 | 4 | 0.0223 |
| D007545---Isoproterenol | 403 | 18 | 13 | 5 | 0.0223 |
| D001581---NA | 9 | 2 | 1 | 1 | 0.0224 |
| D015718---Fibrin Tissue Adhesive | 9 | 2 | 1 | 1 | 0.0224 |
| D029382---Dicarboxylic Acid Transporters | 9 | 2 | 1 | 1 | 0.0224 |
| D056152---Respiratory Rate | 9 | 2 | 2 | 0 | 0.0224 |
| D009929---Organ Size | 1451 | 51 | 34 | 18 | 0.0224 |
| D024661---Potassium Channels, Inwardly Rectifying | 261 | 13 | 6 | 7 | 0.0225 |
| D011503---Transglutaminases | 207 | 11 | 8 | 3 | 0.0226 |
| D036388---Ephrin-B2 | 207 | 11 | 6 | 5 | 0.0226 |
| D054337---Cell Dedifferentiation | 207 | 11 | 8 | 3 | 0.0226 |
| D039564---Integrin alphaV | 130 | 8 | 3 | 5 | 0.0227 |
| D000946---Antigens, Fungal | 61 | 5 | 3 | 2 | 0.0228 |
| D007142---Immunoglobulin gamma-Chains | 61 | 5 | 4 | 1 | 0.0228 |
| D039921---Neuropilins | 61 | 5 | 3 | 2 | 0.0228 |
| D064506---Solute Carrier Family 12, Member 2 | 61 | 5 | 3 | 2 | 0.0228 |
| D003346---Corticotropin-Releasing Hormone | 181 | 10 | 3 | 7 | 0.0229 |
| D004295---Dihydroxyphenylalanine | 41 | 4 | 3 | 1 | 0.0229 |
| D016278---Ventricular Function, Right | 41 | 4 | 4 | 0 | 0.0229 |
| D017480---Receptors, sigma | 41 | 4 | 4 | 0 | 0.0229 |
| D050613---Sodium-Phosphate Cotransporter Proteins, Type IIa | 41 | 4 | 4 | 0 | 0.0229 |
| D051103---1-Acylglycerol-3-Phosphate O-Acyltransferase | 41 | 4 | 4 | 0 | 0.0229 |
| D020733---RNA, Small Cytoplasmic | 83 | 6 | 5 | 1 | 0.023 |
| D022763---CCAAT-Enhancer-Binding Protein-alpha | 318 | 15 | 10 | 5 | 0.0231 |
| D019005---Cystic Fibrosis Transmembrane Conductance Regulator | 262 | 13 | 7 | 6 | 0.0231 |
| D019921---Biotinylation | 494 | 21 | 13 | 8 | 0.0233 |
| D008938---Mitosis | 2099 | 70 | 47 | 24 | 0.0234 |
| D002875---Chromosomes | 1826 | 62 | 46 | 17 | 0.0235 |
| D017874---Immediate-Early Proteins | 678 | 27 | 18 | 9 | 0.0236 |
| D002251---Carbon Tetrachloride | 319 | 15 | 9 | 6 | 0.0237 |
| D005819---Genetic Markers | 2832 | 91 | 63 | 28 | 0.0239 |
| D053547---Keratin-14 | 209 | 11 | 9 | 2 | 0.0241 |
| D024723---Ventricular Myosins | 84 | 6 | 5 | 1 | 0.0243 |
| D005768---Gastrointestinal Hormones | 62 | 5 | 4 | 1 | 0.0243 |
| D006416---Hematoxylin | 62 | 5 | 3 | 2 | 0.0243 |
| D017463---Receptors, Complement 3b | 62 | 5 | 5 | 0 | 0.0243 |
| D017261---Glycosylphosphatidylinositols | 264 | 13 | 11 | 2 | 0.0244 |
| D002921---Cicatrix | 157 | 9 | 5 | 4 | 0.0244 |
| D000954---Antigens, Surface | 775 | 30 | 21 | 9 | 0.0247 |
| D008666---Metalloendopeptidases | 619 | 25 | 18 | 7 | 0.0247 |
| D016564---Amyloid beta-Protein Precursor | 650 | 26 | 13 | 13 | 0.0248 |
| D017494---Proton Pumps | 42 | 4 | 3 | 1 | 0.0248 |
| D027963---Chloride-Bicarbonate Antiporters | 42 | 4 | 2 | 2 | 0.0248 |
| D054678---Cyclic Nucleotide Phosphodiesterases, Type 2 | 42 | 4 | 3 | 1 | 0.0248 |
| D002802---Cholinesterases | 24 | 3 | 3 | 0 | 0.0249 |
| D010852---NA | 24 | 3 | 2 | 1 | 0.0249 |
| D012191---Retroviridae Proteins | 24 | 3 | 3 | 0 | 0.0249 |
| D043588---Receptor, Muscarinic M4 | 24 | 3 | 2 | 1 | 0.0249 |
| D050484---Norepinephrine Plasma Membrane Transport Proteins | 24 | 3 | 2 | 1 | 0.0249 |
| D051347---CapZ Actin Capping Protein | 24 | 3 | 2 | 1 | 0.0249 |
| D011995---Recombination, Genetic | 2037 | 68 | 49 | 20 | 0.025 |
| D000728---Androgens | 379 | 17 | 12 | 5 | 0.025 |
| D018796---Immunoconjugates | 108 | 7 | 6 | 1 | 0.025 |
| D065095---Calcineurin Inhibitors | 108 | 7 | 5 | 2 | 0.025 |
| D025542---Neurofibromin 1 | 184 | 10 | 6 | 4 | 0.0253 |
| D017304---Annexin A5 | 238 | 12 | 9 | 3 | 0.0255 |
| D039663---Integrin beta4 | 85 | 6 | 3 | 3 | 0.0255 |
| D050612---Fatty Acid Transport Proteins | 85 | 6 | 5 | 1 | 0.0255 |
| D053284---Polyunsaturated Alkamides | 85 | 6 | 5 | 1 | 0.0255 |
| D011965---Receptors, Glucocorticoid | 529 | 22 | 16 | 6 | 0.0256 |
| D012689---Sequence Homology, Nucleic Acid | 3194 | 101 | 75 | 27 | 0.0256 |
| D015259---Dopamine Agents | 133 | 8 | 8 | 0 | 0.0256 |
| D026502---LDL-Receptor Related Proteins | 211 | 11 | 5 | 6 | 0.0256 |
| D050760---Cyclin-Dependent Kinase Inhibitor p27 | 652 | 26 | 17 | 9 | 0.0256 |
| D007608---Kainic Acid | 351 | 16 | 8 | 9 | 0.0256 |
| D003181---Complement C4 | 63 | 5 | 4 | 1 | 0.0258 |
| D018621---PrPC Proteins | 109 | 7 | 5 | 2 | 0.0262 |
| D051793---Hypoxia-Inducible Factor 1 | 239 | 12 | 9 | 4 | 0.0262 |
| D051257---Proto-Oncogene Proteins c-maf | 159 | 9 | 7 | 2 | 0.0262 |
| D020917---Receptor, trkA | 267 | 13 | 9 | 4 | 0.0264 |
| D051767---Early Growth Response Protein 2 | 267 | 13 | 9 | 4 | 0.0264 |
| D062826---Zonula Occludens-1 Protein | 267 | 13 | 12 | 1 | 0.0264 |
| D018168---Receptors, Retinoic Acid | 654 | 26 | 17 | 9 | 0.0265 |
| D001425---Bacterial Outer Membrane Proteins | 134 | 8 | 7 | 1 | 0.0266 |
| D017493---Leukocyte Common Antigens | 441 | 19 | 13 | 7 | 0.0267 |
| D000136---Acid-Base Equilibrium | 43 | 4 | 4 | 0 | 0.0268 |
| D053119---NA | 43 | 4 | 4 | 0 | 0.0268 |
| D013045---Species Specificity | 3129 | 99 | 73 | 26 | 0.0268 |
| D000697---Central Nervous System Stimulants | 160 | 9 | 5 | 4 | 0.0272 |
| D039041---Integrin alpha4beta1 | 160 | 9 | 8 | 2 | 0.0272 |
| D049030---Dystroglycans | 160 | 9 | 4 | 5 | 0.0272 |
| D050976---Basic-Leucine Zipper Transcription Factors | 563 | 23 | 16 | 7 | 0.0272 |
| D018095---Germ-Line Mutation | 442 | 19 | 12 | 7 | 0.0272 |
| D018995---Myosin Heavy Chains | 687 | 27 | 17 | 10 | 0.0273 |
| D054558---Protein Tyrosine Phosphatases, Non-Receptor | 110 | 7 | 5 | 3 | 0.0273 |
| D002302---Cardiac Output | 64 | 5 | 3 | 2 | 0.0274 |
| D007535---NA | 64 | 5 | 4 | 1 | 0.0274 |
| D039423---Integrin alpha1 | 64 | 5 | 3 | 2 | 0.0274 |
| D050763---Cyclin-Dependent Kinase Inhibitor p18 | 64 | 5 | 3 | 2 | 0.0274 |
| D051638---Ether-A-Go-Go Potassium Channels | 64 | 5 | 2 | 3 | 0.0274 |
| D000930---Antidiarrheals | 10 | 2 | 1 | 1 | 0.0275 |
| D003055---Cochlear Microphonic Potentials | 10 | 2 | 2 | 0 | 0.0275 |
| D005937---Glucaric Acid | 10 | 2 | 2 | 0 | 0.0275 |
| D008536---Meglumine | 10 | 2 | 0 | 2 | 0.0275 |
| D016281---Oncogene Protein gp140(v-fms) | 10 | 2 | 2 | 0 | 0.0275 |
| D018100---Receptors, Histamine H3 | 10 | 2 | 2 | 0 | 0.0275 |
| D050581---Excitatory Amino Acid Transporter 4 | 10 | 2 | 0 | 2 | 0.0275 |
| D055264---Mucin-4 | 10 | 2 | 2 | 0 | 0.0275 |
| D012880---Skin Pigmentation | 135 | 8 | 4 | 4 | 0.0277 |
| D016627---Oxidopamine | 135 | 8 | 5 | 3 | 0.0277 |
| D018756---GABA Antagonists | 135 | 8 | 5 | 3 | 0.0277 |
| D052006---Bone Morphogenetic Protein Receptors, Type II | 135 | 8 | 6 | 2 | 0.0277 |
| D014809---Vitamin D-Binding Protein | 25 | 3 | 2 | 1 | 0.0278 |
| D018085---Regulon | 25 | 3 | 3 | 1 | 0.0278 |
| D053402---PHEX Phosphate Regulating Neutral Endopeptidase | 25 | 3 | 3 | 0 | 0.0278 |
| D011905---Genes, ras | 626 | 25 | 16 | 9 | 0.0279 |
| D050991---GATA4 Transcription Factor | 384 | 17 | 13 | 4 | 0.0279 |
| D013269---Stochastic Processes | 326 | 15 | 9 | 7 | 0.028 |
| D006239---Haplotypes | 1041 | 38 | 31 | 7 | 0.0281 |
| D015854---Up-Regulation | 3779 | 117 | 86 | 31 | 0.0281 |
| D015321---Gene Rearrangement | 414 | 18 | 16 | 2 | 0.0282 |
| D003603---Cytotoxins | 87 | 6 | 4 | 2 | 0.0282 |
| D017475---Receptors, Nerve Growth Factor | 298 | 14 | 10 | 4 | 0.0284 |
| D009320---Atrial Natriuretic Factor | 242 | 12 | 8 | 4 | 0.0285 |
| D000841---Anisomycin | 111 | 7 | 4 | 3 | 0.0285 |
| D053671---Syndecan-4 | 111 | 7 | 5 | 2 | 0.0285 |
| D053487---DEAD-box RNA Helicases | 566 | 23 | 13 | 10 | 0.0287 |
| D051399---Aquaporin 2 | 136 | 8 | 5 | 3 | 0.0288 |
| D014660---Vasoactive Intestinal Peptide | 188 | 10 | 10 | 0 | 0.0288 |
| D001810---Blood Volume | 44 | 4 | 2 | 2 | 0.0289 |
| D003171---Complement Pathway, Classical | 44 | 4 | 4 | 0 | 0.0289 |
| D018045---Receptors, Oxytocin | 44 | 4 | 2 | 2 | 0.0289 |
| D018733---Nicotinic Antagonists | 44 | 4 | 4 | 0 | 0.0289 |
| D036121---Receptor, EphA3 | 44 | 4 | 2 | 2 | 0.0289 |
| D039223---Cleavage And Polyadenylation Specificity Factor | 44 | 4 | 4 | 0 | 0.0289 |
| D049992---Sodium Chloride Symporters | 44 | 4 | 2 | 2 | 0.0289 |
| D012964---Sodium | 445 | 19 | 12 | 7 | 0.0289 |
| D000726---Androgen Antagonists | 65 | 5 | 3 | 2 | 0.029 |
| D016275---Atrial Function | 65 | 5 | 4 | 1 | 0.029 |
| D053491---Proteinase Inhibitory Proteins, Secretory | 65 | 5 | 2 | 3 | 0.029 |
| D004318---Doxycycline | 386 | 17 | 10 | 7 | 0.0291 |
| D019706---Excitatory Postsynaptic Potentials | 567 | 23 | 13 | 10 | 0.0292 |
| D051966---Phospholipase C gamma | 357 | 16 | 10 | 6 | 0.0293 |
| D009077---Mucins | 271 | 13 | 11 | 2 | 0.0294 |
| D014886---Weaning | 216 | 11 | 9 | 2 | 0.0297 |
| D024701---Myosin Type V | 112 | 7 | 3 | 5 | 0.0298 |
| D044463---Receptor, PAR-1 | 137 | 8 | 5 | 3 | 0.0299 |
| D010146---Pain | 477 | 20 | 13 | 7 | 0.03 |
| D021382---Protein Sorting Signals | 477 | 20 | 11 | 9 | 0.03 |
| D005472---Fluorouracil | 244 | 12 | 8 | 5 | 0.0301 |
| D017448---Receptors, Dopamine D2 | 244 | 12 | 8 | 4 | 0.0301 |
| D016764---Cell Polarity | 1579 | 54 | 30 | 24 | 0.0302 |
| D015127---9,10-Dimethyl-1,2-benzanthracene | 301 | 14 | 12 | 2 | 0.0306 |
| D054878---Lipoylation | 190 | 10 | 7 | 3 | 0.0307 |
| D000083---Acetanilides | 26 | 3 | 2 | 1 | 0.0308 |
| D010095---Oxotremorine | 26 | 3 | 3 | 0 | 0.0308 |
| D051382---GRB7 Adaptor Protein | 26 | 3 | 3 | 0 | 0.0308 |
| D053380---Tumor Necrosis Factor Ligand Superfamily Member 15 | 26 | 3 | 2 | 1 | 0.0308 |
| D055398---Bone Morphogenetic Protein 3 | 26 | 3 | 1 | 2 | 0.0308 |
| D065818---Gamma Rhythm | 26 | 3 | 3 | 0 | 0.0308 |
| D020381---Interleukin-17 | 601 | 24 | 20 | 4 | 0.0308 |
| D010982---Platelet-Derived Growth Factor | 758 | 29 | 22 | 7 | 0.0308 |
| D001833---Body Temperature Regulation | 245 | 12 | 8 | 4 | 0.0309 |
| D015848---Interleukin-5 | 245 | 12 | 12 | 0 | 0.0309 |
| D005810---Multigene Family | 3720 | 115 | 82 | 34 | 0.031 |
| D050720---Retinoblastoma-Like Protein p107 | 138 | 8 | 5 | 3 | 0.031 |
| D018956---CD5 Antigens | 113 | 7 | 6 | 1 | 0.031 |
| D043705---Receptor, Adenosine A2A | 113 | 7 | 4 | 3 | 0.031 |
| D020035---NA | 45 | 4 | 3 | 1 | 0.0311 |
| D020717---Eukaryotic Initiation Factor-2B | 45 | 4 | 2 | 2 | 0.0311 |
| D007980---Levodopa | 89 | 6 | 4 | 2 | 0.0311 |
| D064235---Matrilin Proteins | 89 | 6 | 3 | 3 | 0.0311 |
| D014965---X-Rays | 164 | 9 | 6 | 3 | 0.0312 |
| D050156---Adipogenesis | 1482 | 51 | 37 | 14 | 0.0316 |
| D005966---Glucuronidase | 191 | 10 | 5 | 5 | 0.0316 |
| D003167---Complement Activation | 139 | 8 | 6 | 2 | 0.0322 |
| D007211---NA | 542 | 22 | 13 | 9 | 0.0323 |
| D011955---Receptors, Drug | 114 | 7 | 5 | 2 | 0.0323 |
| D028021---Sodium-Potassium-Chloride Symporters | 114 | 7 | 5 | 2 | 0.0323 |
| D051904---Smad8 Protein | 114 | 7 | 4 | 3 | 0.0323 |
| D010738---Type C Phospholipases | 481 | 20 | 15 | 5 | 0.0324 |
| D005814---Genes, Viral | 219 | 11 | 8 | 3 | 0.0324 |
| D018985---Epitopes, B-Lymphocyte | 90 | 6 | 4 | 2 | 0.0326 |
| D051820---TCF Transcription Factors | 333 | 15 | 11 | 4 | 0.0329 |
| D012260---Ribonucleases | 482 | 20 | 16 | 4 | 0.033 |
| D003272---Contraceptive Agents, Male | 11 | 2 | 1 | 1 | 0.0331 |
| D003487---Cyanoacrylates | 11 | 2 | 1 | 1 | 0.0331 |
| D003890---Desiccation | 11 | 2 | 1 | 1 | 0.0331 |
| D008762---Methylhistidines | 11 | 2 | 2 | 0 | 0.0331 |
| D009489---Neurosecretion | 11 | 2 | 0 | 2 | 0.0331 |
| D014914---Whole Blood Coagulation Time | 11 | 2 | 2 | 0 | 0.0331 |
| D015935---Complement C4b | 11 | 2 | 2 | 0 | 0.0331 |
| D016059---Range of Motion, Articular | 11 | 2 | 2 | 0 | 0.0331 |
| D018010---Receptors, Invertebrate Peptide | 11 | 2 | 2 | 0 | 0.0331 |
| D050937---Guanidinoacetate N-Methyltransferase | 11 | 2 | 0 | 2 | 0.0331 |
| D051561---Complement C3 Convertase, Alternative Pathway | 11 | 2 | 2 | 0 | 0.0331 |
| D053535---Keratin-13 | 11 | 2 | 2 | 0 | 0.0331 |
| D056325---Neuronal Tract-Tracers | 11 | 2 | 2 | 0 | 0.0331 |
| D005990---Glycerol | 166 | 9 | 7 | 2 | 0.0333 |
| D050822---Cytokine Receptor gp130 | 166 | 9 | 7 | 2 | 0.0333 |
| D020032---NA | 140 | 8 | 7 | 1 | 0.0334 |
| D034481---Heterogeneous-Nuclear Ribonucleoprotein Group A-B | 140 | 8 | 4 | 4 | 0.0334 |
| D042702---Angiopoietin-2 | 140 | 8 | 6 | 2 | 0.0334 |
| D000587---Oxidoreductases Acting on CH-NH Group Donors | 46 | 4 | 2 | 2 | 0.0334 |
| D004746---Enoyl-CoA Hydratase | 46 | 4 | 4 | 0 | 0.0334 |
| D005749---Gastric Inhibitory Polypeptide | 46 | 4 | 4 | 0 | 0.0334 |
| D006648---Histocompatibility | 46 | 4 | 4 | 0 | 0.0334 |
| D014563---Urodynamics | 46 | 4 | 1 | 3 | 0.0334 |
| D018004---Receptors, Bombesin | 46 | 4 | 2 | 2 | 0.0334 |
| D018009---Receptors, Glycine | 46 | 4 | 2 | 2 | 0.0334 |
| D051672---Shal Potassium Channels | 46 | 4 | 1 | 3 | 0.0334 |
| D006023---Glycoproteins | 1589 | 54 | 41 | 13 | 0.0335 |
| D005314---Embryonic and Fetal Development | 1965 | 65 | 45 | 21 | 0.0336 |
| D001141---Aromatase | 115 | 7 | 5 | 2 | 0.0337 |
| D009995---Osmosis | 115 | 7 | 4 | 3 | 0.0337 |
| D036801---Parturition | 115 | 7 | 6 | 1 | 0.0337 |
| D004251---DNA Transposable Elements | 423 | 18 | 13 | 5 | 0.0338 |
| D003348---Cortisone | 27 | 3 | 2 | 1 | 0.034 |
| D004296---Dopa Decarboxylase | 27 | 3 | 2 | 1 | 0.034 |
| D011806---Quinolinium Compounds | 27 | 3 | 2 | 1 | 0.034 |
| D014968---Xanthine Dehydrogenase | 27 | 3 | 2 | 1 | 0.034 |
| D050543---Phosphoglycerate Dehydrogenase | 27 | 3 | 1 | 2 | 0.034 |
| D050611---Sodium-Phosphate Cotransporter Proteins, Type III | 27 | 3 | 3 | 0 | 0.034 |
| D053660---Receptors, Interleukin-13 | 27 | 3 | 3 | 0 | 0.034 |
| D006497---Heparitin Sulfate | 277 | 13 | 7 | 6 | 0.0342 |
| D051557---Hepatocyte Nuclear Factor 4 | 277 | 13 | 12 | 1 | 0.0342 |
| D050778---NFATC Transcription Factors | 991 | 36 | 25 | 11 | 0.0342 |
| D012677---Sensation | 68 | 5 | 5 | 0 | 0.0344 |
| D058574---Integrin-Binding Sialoprotein | 68 | 5 | 4 | 1 | 0.0344 |
| D020962---Heterotrimeric GTP-Binding Proteins | 249 | 12 | 6 | 6 | 0.0344 |
| D009418---S100 Proteins | 335 | 15 | 12 | 3 | 0.0344 |
| D020013---Calcium Signaling | 1357 | 47 | 29 | 18 | 0.035 |
| D050811---Octamer Transcription Factor-1 | 116 | 7 | 6 | 1 | 0.0351 |
| D051401---Aquaporin 4 | 116 | 7 | 6 | 1 | 0.0351 |
| D016827---CD8 Antigens | 455 | 19 | 14 | 6 | 0.0351 |
| D007633---Keratins | 516 | 21 | 16 | 5 | 0.0351 |
| D017930---Genes, Reporter | 2950 | 93 | 65 | 28 | 0.0351 |
| D019006---Neural Cell Adhesion Molecules | 336 | 15 | 11 | 4 | 0.0352 |
| D018048---Receptors, Purinergic P2 | 222 | 11 | 8 | 3 | 0.0352 |
| D005456---Fluorescent Dyes | 896 | 33 | 22 | 11 | 0.0353 |
| D050990---GATA3 Transcription Factor | 768 | 29 | 24 | 5 | 0.0357 |
| D015816---Cell Adhesion Molecules, Neuronal | 610 | 24 | 15 | 9 | 0.0357 |
| D000451---Mineralocorticoid Receptor Antagonists | 47 | 4 | 1 | 3 | 0.0357 |
| D043585---Receptor, Muscarinic M2 | 47 | 4 | 3 | 1 | 0.0357 |
| D014759---Viral Envelope Proteins | 279 | 13 | 11 | 2 | 0.0359 |
| D019408---Platelet Endothelial Cell Adhesion Molecule-1 | 487 | 20 | 15 | 5 | 0.0361 |
| D007986---Luteinizing Hormone | 251 | 12 | 8 | 4 | 0.0362 |
| D003360---Cosmids | 367 | 16 | 8 | 8 | 0.0364 |
| D008222---Lymphokines | 457 | 19 | 14 | 5 | 0.0364 |
| D011347---Procollagen | 117 | 7 | 3 | 4 | 0.0365 |
| D020934---Ciliary Neurotrophic Factor | 117 | 7 | 5 | 2 | 0.0365 |
| D058125---Transcription Factor 7-Like 1 Protein | 117 | 7 | 6 | 1 | 0.0365 |
| D011972---Receptor, Insulin | 427 | 18 | 14 | 4 | 0.0366 |
| D010196---Pancreatic Elastase | 196 | 10 | 7 | 3 | 0.0368 |
| D018389---Codon, Nonsense | 338 | 15 | 11 | 4 | 0.0368 |
| D051766---Early Growth Response Protein 1 | 338 | 15 | 7 | 8 | 0.0368 |
| D051761---Paired Box Transcription Factors | 1030 | 37 | 23 | 15 | 0.037 |
| D012794---Sialic Acids | 143 | 8 | 4 | 4 | 0.0371 |
| D019812---Heparan Sulfate Proteoglycans | 252 | 12 | 7 | 5 | 0.0372 |
| D025721---WT1 Proteins | 224 | 11 | 9 | 2 | 0.0372 |
| D000777---Anesthetics | 28 | 3 | 3 | 0 | 0.0373 |
| D012027---Reflex, Vestibulo-Ocular | 28 | 3 | 2 | 1 | 0.0373 |
| D019081---O Antigens | 28 | 3 | 1 | 2 | 0.0373 |
| D019375---NA | 28 | 3 | 2 | 1 | 0.0373 |
| D024081---Collagen Type IX | 28 | 3 | 2 | 1 | 0.0373 |
| D026962---Amino Acid Transport System ASC | 28 | 3 | 0 | 3 | 0.0373 |
| D044102---Receptor, Melanocortin, Type 1 | 28 | 3 | 2 | 1 | 0.0373 |
| D053144---Cell Nucleus Shape | 28 | 3 | 2 | 1 | 0.0373 |
| D036386---Ephrin-A5 | 93 | 6 | 3 | 3 | 0.0374 |
| D054769---G-Protein-Coupled Receptor Kinase 2 | 93 | 6 | 3 | 3 | 0.0374 |
| D064568---S100 Calcium Binding Protein beta Subunit | 93 | 6 | 4 | 2 | 0.0374 |
| D003911---Dextrans | 170 | 9 | 8 | 1 | 0.0379 |
| D018021---Lithium Chloride | 170 | 9 | 4 | 5 | 0.0379 |
| D005696---Galactosidases | 48 | 4 | 1 | 3 | 0.0382 |
| D013433---Sulfoglycosphingolipids | 48 | 4 | 2 | 2 | 0.0382 |
| D017320---HIV Protease Inhibitors | 48 | 4 | 3 | 1 | 0.0382 |
| D022061---Tacrolimus Binding Protein 1A | 48 | 4 | 2 | 2 | 0.0382 |
| D053319---Tumor Necrosis Factor Decoy Receptors | 48 | 4 | 2 | 2 | 0.0382 |
| D054594---Protein Tyrosine Phosphatase, Non-Receptor Type 12 | 48 | 4 | 4 | 0 | 0.0382 |
| D020598---Proto-Oncogene Proteins c-myb | 225 | 11 | 9 | 2 | 0.0383 |
| D039161---Integrin alpha6beta4 | 70 | 5 | 4 | 1 | 0.0383 |
| D029961---Zebrafish Proteins | 1033 | 37 | 25 | 12 | 0.0384 |
| D007335---Insulin-Like Growth Factor II | 340 | 15 | 10 | 5 | 0.0384 |
| D004361---Drug Tolerance | 144 | 8 | 6 | 2 | 0.0384 |
| D060466---Axin Protein | 282 | 13 | 6 | 7 | 0.0386 |
| D057134---Antibodies, Neutralizing | 311 | 14 | 11 | 3 | 0.0386 |
| D015500---Chloramphenicol O-Acetyltransferase | 400 | 17 | 11 | 6 | 0.0389 |
| D017447---Receptors, Dopamine D1 | 198 | 10 | 6 | 4 | 0.039 |
| D005445---Flunitrazepam | 12 | 2 | 2 | 0 | 0.039 |
| D012472---NA | 12 | 2 | 1 | 1 | 0.039 |
| D017680---Potassium Compounds | 12 | 2 | 1 | 1 | 0.039 |
| D018042---Receptors, Neurokinin-3 | 12 | 2 | 1 | 1 | 0.039 |
| D050614---Sodium-Phosphate Cotransporter Proteins, Type IIb | 12 | 2 | 1 | 1 | 0.039 |
| D053381---Secretogranin II | 12 | 2 | 0 | 2 | 0.039 |
| D064801---Phospholipase A2 Inhibitors | 12 | 2 | 2 | 0 | 0.039 |
| D002268---NA | 94 | 6 | 3 | 3 | 0.0391 |
| D054413---Chemokine CCL11 | 94 | 6 | 6 | 0 | 0.0391 |
| D009930---Organic Chemicals | 171 | 9 | 5 | 4 | 0.0391 |
| D006085---Graft Survival | 341 | 15 | 12 | 3 | 0.0392 |
| D015675---Osteocalcin | 341 | 15 | 9 | 6 | 0.0392 |
| D044783---F-Box Proteins | 431 | 18 | 10 | 8 | 0.0395 |
| D002882---Chromosomes, Human, Pair 13 | 145 | 8 | 6 | 2 | 0.0398 |
| D008156---Luciferases | 1709 | 57 | 38 | 19 | 0.04 |
| D000431---Ethanol | 649 | 25 | 16 | 9 | 0.0402 |
| D004338---Drug Combinations | 432 | 18 | 13 | 5 | 0.0403 |
| D008734---Methoxyhydroxyphenylglycol | 71 | 5 | 3 | 2 | 0.0403 |
| D011345---Fenofibrate | 71 | 5 | 4 | 1 | 0.0403 |
| D020756---Coatomer Protein | 71 | 5 | 2 | 3 | 0.0403 |
| D040321---Vascular Endothelial Growth Factor Receptor-3 | 172 | 9 | 6 | 3 | 0.0404 |
| D053674---Aggrecans | 172 | 9 | 7 | 2 | 0.0404 |
| D020029---Base Pairing | 227 | 11 | 9 | 2 | 0.0404 |
| D050437---Genes, Developmental | 313 | 14 | 9 | 5 | 0.0404 |
| D005968---Glutamate Decarboxylase | 284 | 13 | 9 | 4 | 0.0405 |
| D018398---Homeodomain Proteins | 3469 | 107 | 74 | 34 | 0.0405 |
| D001052---Apoferritins | 49 | 4 | 3 | 1 | 0.0407 |
| D034802---RNA-Binding Protein EWS | 49 | 4 | 2 | 2 | 0.0407 |
| D043704---Receptor, Adenosine A2B | 49 | 4 | 4 | 0 | 0.0407 |
| D052119---Lysosomal-Associated Membrane Protein 2 | 95 | 6 | 2 | 4 | 0.0408 |
| D010622---Phencyclidine | 29 | 3 | 2 | 1 | 0.0409 |
| D019379---Teriparatide | 29 | 3 | 3 | 0 | 0.0409 |
| D020099---Coated Materials, Biocompatible | 29 | 3 | 2 | 1 | 0.0409 |
| D050491---Glutamate Plasma Membrane Transport Proteins | 29 | 3 | 1 | 2 | 0.0409 |
| D051179---Plakins | 29 | 3 | 2 | 1 | 0.0409 |
| D061027---Programmed Cell Death 1 Ligand 2 Protein | 29 | 3 | 3 | 0 | 0.0409 |
| D016187---Receptors, Granulocyte-Macrophage Colony-Stimulating Factor | 120 | 7 | 6 | 1 | 0.041 |
| D011188---NA | 256 | 12 | 7 | 5 | 0.0411 |
| D010959---Tissue Plasminogen Activator | 146 | 8 | 3 | 5 | 0.0412 |
| D001482---Base Composition | 314 | 14 | 13 | 1 | 0.0413 |
| D002560---Cerebrovascular Circulation | 285 | 13 | 7 | 6 | 0.0414 |
| D005337---Fibrin | 173 | 9 | 7 | 2 | 0.0416 |
| D010710---Phosphates | 315 | 14 | 9 | 5 | 0.0423 |
| D014841---von Willebrand Factor | 229 | 11 | 9 | 2 | 0.0426 |
| D054405---Chemokine CCL3 | 147 | 8 | 7 | 1 | 0.0426 |
| D058750---Epithelial-Mesenchymal Transition | 749 | 28 | 20 | 8 | 0.0429 |
| D019000---Phosphotyrosine | 528 | 21 | 17 | 4 | 0.0431 |
| D016900---Neurofilament Proteins | 316 | 14 | 8 | 6 | 0.0432 |
| D039001---Integrin alpha Chains | 258 | 12 | 9 | 3 | 0.0432 |
| D000597---Amino Acids, Branched-Chain | 50 | 4 | 3 | 1 | 0.0434 |
| D004253---DNA Nucleotidylexotransferase | 50 | 4 | 3 | 1 | 0.0434 |
| D015347---RNA Probes | 2518 | 80 | 59 | 21 | 0.0436 |
| D011980---Receptors, Progesterone | 346 | 15 | 10 | 5 | 0.0436 |
| D000494---Allosteric Regulation | 202 | 10 | 5 | 5 | 0.0437 |
| D000944---Antigens, Differentiation, B-Lymphocyte | 230 | 11 | 10 | 1 | 0.0437 |
| D003851---Deoxyribonucleases | 122 | 7 | 4 | 3 | 0.0441 |
| D009698---Nucleoproteins | 122 | 7 | 6 | 1 | 0.0441 |
| D012194---RNA-Directed DNA Polymerase | 122 | 7 | 4 | 3 | 0.0441 |
| D018053---NA | 122 | 7 | 5 | 2 | 0.0441 |
| D000254---Sodium-Potassium-Exchanging ATPase | 317 | 14 | 8 | 6 | 0.0442 |
| D003971---Diastole | 97 | 6 | 5 | 1 | 0.0445 |
| D002034---Bumetanide | 30 | 3 | 2 | 1 | 0.0445 |
| D014048---Tolonium Chloride | 30 | 3 | 3 | 0 | 0.0445 |
| D019405---Cytochrome P-450 CYP11B2 | 30 | 3 | 1 | 2 | 0.0445 |
| D051664---Kv1.3 Potassium Channel | 30 | 3 | 2 | 1 | 0.0445 |
| D056850---Cyclin-Dependent Kinase 8 | 30 | 3 | 3 | 0 | 0.0445 |
| D057891---Tetraploidy | 30 | 3 | 2 | 1 | 0.0445 |
| D001837---Body Weights and Measures | 73 | 5 | 3 | 2 | 0.0446 |
| D011627---Puberty | 73 | 5 | 4 | 1 | 0.0446 |
| D012702---Serotonin Antagonists | 73 | 5 | 5 | 0 | 0.0446 |
| D055415---Bone Morphogenetic Protein 4 | 562 | 22 | 11 | 11 | 0.0452 |
| D016259---Genes, myc | 625 | 24 | 19 | 6 | 0.0452 |
| D003175---Complement C2 | 13 | 2 | 2 | 0 | 0.0453 |
| D005977---NA | 13 | 2 | 1 | 1 | 0.0453 |
| D043424---Carboxypeptidase B | 13 | 2 | 2 | 0 | 0.0453 |
| D050559---beta-Carotene 15,15'-Monooxygenase | 13 | 2 | 2 | 0 | 0.0453 |
| D050682---Q-SNARE Proteins | 13 | 2 | 2 | 0 | 0.0453 |
| D054743---Cyclic AMP-Dependent Protein Kinase Type I | 13 | 2 | 1 | 1 | 0.0453 |
| D015431---Weight Loss | 289 | 13 | 9 | 4 | 0.0454 |
| D018793---Interleukin-13 | 378 | 16 | 14 | 3 | 0.0455 |
| D050436---Regulatory Elements, Transcriptional | 232 | 11 | 7 | 4 | 0.046 |
| D004924---Escape Reaction | 51 | 4 | 3 | 1 | 0.0461 |
| D053505---Matrix Metalloproteinases, Secreted | 51 | 4 | 1 | 3 | 0.0461 |
| D004627---Embryonic Induction | 595 | 23 | 17 | 7 | 0.0463 |
| D000951---Antigens, Neoplasm | 917 | 33 | 18 | 15 | 0.0463 |
| D018819---Dipeptidyl Peptidase 4 | 98 | 6 | 4 | 2 | 0.0464 |
| D020746---Calcium Channels, L-Type | 349 | 15 | 9 | 6 | 0.0464 |
| D047888---Receptors, Tumor Necrosis Factor, Type I | 349 | 15 | 13 | 2 | 0.0464 |
| D001823---Body Composition | 379 | 16 | 12 | 4 | 0.0464 |
| D005803---Genes, Immunoglobulin | 290 | 13 | 5 | 8 | 0.0465 |
| D003900---Desoxycorticosterone | 74 | 5 | 3 | 2 | 0.0468 |
| D018040---Receptors, Neurokinin-1 | 74 | 5 | 4 | 1 | 0.0468 |
| D050756---Cyclin-Dependent Kinase Inhibitor Proteins | 74 | 5 | 2 | 3 | 0.0468 |
| D020782---Matrix Metalloproteinases | 533 | 21 | 14 | 7 | 0.0469 |
| D054377---Chemokine CXCL12 | 471 | 19 | 14 | 5 | 0.0469 |
| D019869---Phosphatidylinositol 3-Kinases | 1795 | 59 | 38 | 21 | 0.047 |
| D004676---Myelin Basic Protein | 320 | 14 | 11 | 3 | 0.0471 |
| D011061---Poly A | 410 | 17 | 13 | 4 | 0.0471 |
| D015640---Ion Channel Gating | 350 | 15 | 10 | 5 | 0.0474 |
| D017471---Receptors, Interferon | 205 | 10 | 9 | 1 | 0.0474 |
| D050791---STAT Transcription Factors | 205 | 10 | 9 | 1 | 0.0474 |
| D052138---Genes, Neoplasm | 124 | 7 | 4 | 3 | 0.0475 |
| D019746---Intramolecular Oxidoreductases | 291 | 13 | 11 | 2 | 0.0475 |
| D017384---Sequence Deletion | 2005 | 65 | 47 | 18 | 0.048 |
| D056913---Mediator Complex Subunit 1 | 99 | 6 | 5 | 1 | 0.0483 |
| D014442---Monophenol Monooxygenase | 178 | 9 | 6 | 3 | 0.0484 |
| D020797---Receptor, Platelet-Derived Growth Factor beta | 351 | 15 | 11 | 4 | 0.0484 |
| D018847---Opioid Peptides | 31 | 3 | 1 | 2 | 0.0484 |
| D027301---Fusion Regulatory Protein 1, Light Chains | 31 | 3 | 2 | 1 | 0.0484 |
| D050580---Excitatory Amino Acid Transporter 3 | 31 | 3 | 1 | 2 | 0.0484 |
| D017470---Receptors, Glutamate | 234 | 11 | 7 | 5 | 0.0484 |
| D047495---PPAR gamma | 1086 | 38 | 27 | 11 | 0.0485 |
| D016392---Proto-Oncogene Proteins pp60(c-src) | 292 | 13 | 10 | 3 | 0.0486 |
| D002894---Chromosomes, Human, Pair 4 | 206 | 10 | 7 | 3 | 0.0487 |
| D004573---NA | 52 | 4 | 2 | 2 | 0.049 |
| D009961---Orosomucoid | 52 | 4 | 4 | 0 | 0.049 |
| D011962---Receptors, FSH | 52 | 4 | 3 | 1 | 0.049 |
| D012439---Saccharin | 52 | 4 | 3 | 1 | 0.049 |
| D062827---Zonula Occludens-2 Protein | 52 | 4 | 3 | 1 | 0.049 |
| D018919---Neovascularization, Physiologic | 1457 | 49 | 34 | 15 | 0.049 |
| D007671---Kidney Concentrating Ability | 75 | 5 | 3 | 2 | 0.0491 |
| D038982---Integrin alpha2beta1 | 75 | 5 | 4 | 1 | 0.0491 |
| D011388---Prolactin | 322 | 14 | 8 | 6 | 0.0492 |
| D018123---Receptors, Interleukin | 322 | 14 | 13 | 1 | 0.0492 |
| D016282---Aspartic Acid Endopeptidases | 352 | 15 | 11 | 4 | 0.0493 |
| D007166---Immunosuppressive Agents | 505 | 20 | 14 | 6 | 0.0494 |
| D016044---Fusion Proteins, bcr-abl | 235 | 11 | 9 | 2 | 0.0496 |
| D058539---Phosphatidylinositol 3-Kinase | 293 | 13 | 8 | 5 | 0.0497 |
| D016220---Fibroblast Growth Factor 1 | 179 | 9 | 7 | 2 | 0.0498 |
| D038362---Glycogen Synthase Kinase 3 | 825 | 30 | 21 | 9 | 0.05 |

**Supplemental Table 5.** Gene Ontology (GO) pathways enriched with folic acid supplementation (FDR ≤ 5%). Sorted most significant to least significant using *p*-value. Abbreviations: Sig, significant; UP, upregulated; DOWN, downregulated

| **Term** | **Total genes** | **Sig. genes** | **# UP** | **# DOWN** | ***p* – value** |
| --- | --- | --- | --- | --- | --- |
| GO:0010628---positive regulation of gene expression | 339 | 11 | 5 | 6 | 0.000708 |
| GO:0030199---collagen fibril organization | 40 | 4 | 3 | 1 | 0.000718 |
| GO:0043044---ATP-dependent chromatin remodeling | 19 | 3 | 2 | 1 | 0.000899 |
| GO:0033010---paranodal junction | 6 | 2 | 2 | 0 | 0.001516 |
| GO:0007017---microtubule-based process | 26 | 3 | 3 | 0 | 0.002288 |
| GO:0019226---transmission of nerve impulse | 8 | 2 | 1 | 1 | 0.002792 |
| GO:0030235---nitric-oxide synthase regulator activity | 8 | 2 | 1 | 1 | 0.002792 |
| GO:0042809---vitamin D receptor binding | 8 | 2 | 1 | 1 | 0.002792 |
| GO:0062023---collagen-containing extracellular matrix | 196 | 7 | 6 | 1 | 0.003988 |
| GO:0010524---positive regulation of calcium ion transport into cytosol | 10 | 2 | 1 | 1 | 0.004427 |
| GO:0043653---mitochondrial fragmentation involved in apoptotic process | 10 | 2 | 2 | 0 | 0.004427 |
| GO:0045892---negative regulation of transcription, DNA-templated | 443 | 11 | 7 | 4 | 0.005715 |
| GO:0001725---stress fiber | 71 | 4 | 1 | 3 | 0.005983 |
| GO:0001664---G protein-coupled receptor binding | 39 | 3 | 2 | 1 | 0.007304 |
| GO:0043407---negative regulation of MAP kinase activity | 39 | 3 | 0 | 3 | 0.007304 |
| GO:0007422---peripheral nervous system development | 13 | 2 | 1 | 1 | 0.007521 |
| GO:0045019---negative regulation of nitric oxide biosynthetic process | 13 | 2 | 1 | 1 | 0.007521 |
| GO:0004930---G protein-coupled receptor activity | 120 | 5 | 4 | 1 | 0.007737 |
| GO:0010498---proteasomal protein catabolic process | 14 | 2 | 1 | 1 | 0.008717 |
| GO:0090336---positive regulation of brown fat cell differentiation | 14 | 2 | 1 | 1 | 0.008717 |
| GO:0000188---inactivation of MAPK activity | 15 | 2 | 0 | 2 | 0.009991 |
| GO:0005200---structural constituent of cytoskeleton | 45 | 3 | 3 | 0 | 0.010848 |
| GO:1901800---positive regulation of proteasomal protein catabolic process | 16 | 2 | 2 | 0 | 0.011342 |
| GO:0043195---terminal bouton | 46 | 3 | 2 | 1 | 0.01152 |
| GO:0000976---transcription regulatory region sequence-specific DNA binding | 185 | 6 | 4 | 2 | 0.011821 |
| GO:0003700---DNA-binding transcription factor activity | 365 | 9 | 5 | 4 | 0.012603 |
| GO:0045505---dynein intermediate chain binding | 17 | 2 | 1 | 1 | 0.012769 |
| GO:0050998---nitric-oxide synthase binding | 17 | 2 | 0 | 2 | 0.012769 |
| GO:0071360---cellular response to exogenous dsRNA | 17 | 2 | 1 | 1 | 0.012769 |
| GO:0097038---perinuclear endoplasmic reticulum | 17 | 2 | 2 | 0 | 0.012769 |
| GO:1902236---negative regulation of endoplasmic reticulum stress-induced intrinsic apoptotic signaling pathway | 17 | 2 | 1 | 1 | 0.012769 |
| GO:0051959---dynein light intermediate chain binding | 18 | 2 | 1 | 1 | 0.01427 |
| GO:0006869---lipid transport | 92 | 4 | 4 | 0 | 0.014612 |
| GO:0000242---pericentriolar material | 19 | 2 | 1 | 1 | 0.015843 |
| GO:0051480---regulation of cytosolic calcium ion concentration | 19 | 2 | 1 | 1 | 0.015843 |
| GO:0080182---histone H3-K4 trimethylation | 19 | 2 | 0 | 2 | 0.015843 |
| GO:0042802---identical protein binding | 1336 | 22 | 15 | 7 | 0.016356 |
| GO:0014003---oligodendrocyte development | 20 | 2 | 0 | 2 | 0.017487 |
| GO:0031226---intrinsic component of plasma membrane | 20 | 2 | 1 | 1 | 0.017487 |
| GO:0005080---protein kinase C binding | 54 | 3 | 2 | 1 | 0.017747 |
| GO:0007186---G protein-coupled receptor signaling pathway | 205 | 6 | 4 | 2 | 0.018735 |
| GO:0042826---histone deacetylase binding | 101 | 4 | 3 | 1 | 0.019915 |
| GO:0019904---protein domain specific binding | 268 | 7 | 5 | 2 | 0.020164 |
| GO:0048306---calcium-dependent protein binding | 57 | 3 | 2 | 1 | 0.02048 |
| GO:0005868---cytoplasmic dynein complex | 22 | 2 | 1 | 1 | 0.02098 |
| GO:0005875---microtubule associated complex | 22 | 2 | 2 | 0 | 0.02098 |
| GO:0045121---membrane raft | 212 | 6 | 5 | 1 | 0.021699 |
| GO:0043433---negative regulation of DNA-binding transcription factor activity | 59 | 3 | 3 | 0 | 0.022423 |
| GO:0001782---B cell homeostasis | 23 | 2 | 1 | 1 | 0.022826 |
| GO:0033137---negative regulation of peptidyl-serine phosphorylation | 23 | 2 | 1 | 1 | 0.022826 |
| GO:0010629---negative regulation of gene expression | 215 | 6 | 5 | 1 | 0.023061 |
| GO:0001975---response to amphetamine | 24 | 2 | 0 | 2 | 0.024737 |
| GO:0006368---transcription elongation from RNA polymerase II promoter | 24 | 2 | 0 | 2 | 0.024737 |
| GO:0030286---dynein complex | 24 | 2 | 1 | 1 | 0.024737 |
| GO:0008285---negative regulation of cell population proliferation | 283 | 7 | 5 | 2 | 0.026189 |
| GO:0005102---signaling receptor binding | 222 | 6 | 5 | 1 | 0.02646 |
| GO:0051881---regulation of mitochondrial membrane potential | 25 | 2 | 2 | 0 | 0.02671 |
| GO:0008134---transcription factor binding | 286 | 7 | 6 | 1 | 0.027528 |
| GO:0045944---positive regulation of transcription by RNA polymerase II | 855 | 15 | 8 | 7 | 0.028077 |
| GO:0060090---molecular adaptor activity | 65 | 3 | 1 | 2 | 0.028834 |
| GO:0005901---caveola | 66 | 3 | 0 | 3 | 0.029987 |
| GO:0018024---histone-lysine N-methyltransferase activity | 27 | 2 | 1 | 1 | 0.030841 |
| GO:0032870---cellular response to hormone stimulus | 27 | 2 | 1 | 1 | 0.030841 |
| GO:0048147---negative regulation of fibroblast proliferation | 27 | 2 | 2 | 0 | 0.030841 |
| GO:0008013---beta-catenin binding | 68 | 3 | 3 | 0 | 0.032366 |
| GO:0006641---triglyceride metabolic process | 28 | 2 | 1 | 1 | 0.032995 |
| GO:0043388---positive regulation of DNA binding | 28 | 2 | 1 | 1 | 0.032995 |
| GO:2001244---positive regulation of intrinsic apoptotic signaling pathway | 28 | 2 | 1 | 1 | 0.032995 |
| GO:0031072---heat shock protein binding | 69 | 3 | 3 | 0 | 0.033591 |
| GO:0001228---DNA-binding transcription activator activity, RNA polymerase II-specific | 301 | 7 | 5 | 2 | 0.034921 |
| GO:0043488---regulation of mRNA stability | 29 | 2 | 2 | 0 | 0.035206 |
| GO:0048662---negative regulation of smooth muscle cell proliferation | 29 | 2 | 1 | 1 | 0.035206 |
| GO:0070615---nucleosome-dependent ATPase activity | 29 | 2 | 1 | 1 | 0.035206 |
| GO:0031624---ubiquitin conjugating enzyme binding | 30 | 2 | 2 | 0 | 0.037473 |
| GO:0035774---positive regulation of insulin secretion involved in cellular response to glucose stimulus | 30 | 2 | 2 | 0 | 0.037473 |
| GO:0043085---positive regulation of catalytic activity | 30 | 2 | 1 | 1 | 0.037473 |
| GO:0002862---negative regulation of inflammatory response to antigenic stimulus | 4 | 1 | 0 | 1 | 0.040295 |
| GO:0002904---positive regulation of B cell apoptotic process | 4 | 1 | 1 | 0 | 0.040295 |
| GO:0004859---phospholipase inhibitor activity | 4 | 1 | 1 | 0 | 0.040295 |
| GO:0005513---detection of calcium ion | 4 | 1 | 0 | 1 | 0.040295 |
| GO:0008170---N-methyltransferase activity | 4 | 1 | 0 | 1 | 0.040295 |
| GO:0008535---respiratory chain complex IV assembly | 4 | 1 | 1 | 0 | 0.040295 |
| GO:0010626---negative regulation of Schwann cell proliferation | 4 | 1 | 0 | 1 | 0.040295 |
| GO:0010636---positive regulation of mitochondrial fusion | 4 | 1 | 1 | 0 | 0.040295 |
| GO:0010753---positive regulation of cGMP-mediated signaling | 4 | 1 | 1 | 0 | 0.040295 |
| GO:0015117---thiosulfate transmembrane transporter activity | 4 | 1 | 0 | 1 | 0.040295 |
| GO:0015131---oxaloacetate transmembrane transporter activity | 4 | 1 | 0 | 1 | 0.040295 |
| GO:0015140---malate transmembrane transporter activity | 4 | 1 | 0 | 1 | 0.040295 |
| GO:0015141---succinate transmembrane transporter activity | 4 | 1 | 0 | 1 | 0.040295 |
| GO:0015709---thiosulfate transport | 4 | 1 | 0 | 1 | 0.040295 |
| GO:0015729---oxaloacetate transport | 4 | 1 | 0 | 1 | 0.040295 |
| GO:0030368---interleukin-17 receptor activity | 4 | 1 | 1 | 0 | 0.040295 |
| GO:0031315---extrinsic component of mitochondrial outer membrane | 4 | 1 | 0 | 1 | 0.040295 |
| GO:0031800---type 3 metabotropic glutamate receptor binding | 4 | 1 | 0 | 1 | 0.040295 |
| GO:0032507---maintenance of protein location in cell | 4 | 1 | 0 | 1 | 0.040295 |
| GO:0033612---receptor serine/threonine kinase binding | 4 | 1 | 0 | 1 | 0.040295 |
| GO:0035166---post-embryonic hemopoiesis | 4 | 1 | 1 | 0 | 0.040295 |
| GO:0043372---positive regulation of CD4-positive, alpha-beta T cell differentiation | 4 | 1 | 0 | 1 | 0.040295 |
| GO:0045098---type III intermediate filament | 4 | 1 | 1 | 0 | 0.040295 |
| GO:0046985---positive regulation of hemoglobin biosynthetic process | 4 | 1 | 1 | 0 | 0.040295 |
| GO:0048495---Roundabout binding | 4 | 1 | 1 | 0 | 0.040295 |
| GO:0048550---negative regulation of pinocytosis | 4 | 1 | 0 | 1 | 0.040295 |
| GO:0048730---epidermis morphogenesis | 4 | 1 | 1 | 0 | 0.040295 |
| GO:0050994---regulation of lipid catabolic process | 4 | 1 | 1 | 0 | 0.040295 |
| GO:0051005---negative regulation of lipoprotein lipase activity | 4 | 1 | 1 | 0 | 0.040295 |
| GO:0051343---positive regulation of cyclic-nucleotide phosphodiesterase activity | 4 | 1 | 0 | 1 | 0.040295 |
| GO:0051861---glycolipid binding | 4 | 1 | 1 | 0 | 0.040295 |
| GO:0055069---zinc ion homeostasis | 4 | 1 | 1 | 0 | 0.040295 |
| GO:0055131---C3HC4-type RING finger domain binding | 4 | 1 | 1 | 0 | 0.040295 |
| GO:0060058---positive regulation of apoptotic process involved in mammary gland involution | 4 | 1 | 1 | 0 | 0.040295 |
| GO:0060355---positive regulation of cell adhesion molecule production | 4 | 1 | 0 | 1 | 0.040295 |
| GO:0060452---positive regulation of cardiac muscle contraction | 4 | 1 | 0 | 1 | 0.040295 |
| GO:0060510---type II pneumocyte differentiation | 4 | 1 | 1 | 0 | 0.040295 |
| GO:0061041---regulation of wound healing | 4 | 1 | 0 | 1 | 0.040295 |
| GO:0061673---mitotic spindle astral microtubule | 4 | 1 | 0 | 1 | 0.040295 |
| GO:0061689---tricellular tight junction | 4 | 1 | 1 | 0 | 0.040295 |
| GO:0070242---thymocyte apoptotic process | 4 | 1 | 1 | 0 | 0.040295 |
| GO:0070307---lens fiber cell development | 4 | 1 | 1 | 0 | 0.040295 |
| GO:0070579---methylcytosine dioxygenase activity | 4 | 1 | 0 | 1 | 0.040295 |
| GO:0070842---aggresome assembly | 4 | 1 | 1 | 0 | 0.040295 |
| GO:0070966---nuclear-transcribed mRNA catabolic process, no-go decay | 4 | 1 | 1 | 0 | 0.040295 |
| GO:0071409---cellular response to cycloheximide | 4 | 1 | 1 | 0 | 0.040295 |
| GO:0071422---succinate transmembrane transport | 4 | 1 | 0 | 1 | 0.040295 |
| GO:0071423---malate transmembrane transport | 4 | 1 | 0 | 1 | 0.040295 |
| GO:0072584---caveolin-mediated endocytosis | 4 | 1 | 0 | 1 | 0.040295 |
| GO:0098684---photoreceptor ribbon synapse | 4 | 1 | 1 | 0 | 0.040295 |
| GO:0098901---regulation of cardiac muscle cell action potential | 4 | 1 | 0 | 1 | 0.040295 |
| GO:1902262---apoptotic process involved in blood vessel morphogenesis | 4 | 1 | 1 | 0 | 0.040295 |
| GO:1902356---oxaloacetate(2-) transmembrane transport | 4 | 1 | 0 | 1 | 0.040295 |
| GO:1902512---positive regulation of apoptotic DNA fragmentation | 4 | 1 | 1 | 0 | 0.040295 |
| GO:1903377---negative regulation of oxidative stress-induced neuron intrinsic apoptotic signaling pathway | 4 | 1 | 1 | 0 | 0.040295 |
| GO:1903542---negative regulation of exosomal secretion | 4 | 1 | 1 | 0 | 0.040295 |
| GO:1903598---positive regulation of gap junction assembly | 4 | 1 | 0 | 1 | 0.040295 |
| GO:1904399---heparan sulfate binding | 4 | 1 | 0 | 1 | 0.040295 |
| GO:1904798---positive regulation of core promoter binding | 4 | 1 | 1 | 0 | 0.040295 |
| GO:1990254---keratin filament binding | 4 | 1 | 1 | 0 | 0.040295 |
| GO:1990444---F-box domain binding | 4 | 1 | 1 | 0 | 0.040295 |
| GO:2000535---regulation of entry of bacterium into host cell | 4 | 1 | 0 | 1 | 0.040295 |
| GO:0000978---RNA polymerase II cis-regulatory region sequence-specific DNA binding | 668 | 12 | 7 | 5 | 0.040713 |
| GO:0032092---positive regulation of protein binding | 75 | 3 | 1 | 2 | 0.041443 |
| GO:0050873---brown fat cell differentiation | 33 | 2 | 1 | 1 | 0.044595 |
| GO:0051898---negative regulation of protein kinase B signaling | 33 | 2 | 1 | 1 | 0.044595 |
| GO:0000122---negative regulation of transcription by RNA polymerase II | 681 | 12 | 7 | 5 | 0.046003 |
| GO:0001541---ovarian follicle development | 34 | 2 | 1 | 1 | 0.047072 |
| GO:0042803---protein homodimerization activity | 534 | 10 | 5 | 5 | 0.047073 |
| GO:0005515---protein binding | 4927 | 60 | 39 | 21 | 0.04982 |

**Supplemental Table 6.** Kyoto Encyclopedia of Genes and Genomes (KEGG) pathways enriched with folic acid supplementation (FDR ≤ 5%). Sorted most significant to least significant using *p*-value. Abbreviations: Sig, significant; UP, upregulated; DOWN, downregulated

| **Term** | **Total genes** | **Sig. genes** | **# UP** | **# DOWN** | ***p* – value** |
| --- | --- | --- | --- | --- | --- |
| mmu04740 Olfactory transduction | 30 | 2 | 0 | 2 | 0.033712 |
| mmu00430 Taurine and hypotaurine metabolism | 5 | 1 | 1 | 0 | 0.047591 |

**Supplemental Table 7.** Medical Subject Headings (MeSH) pathways enriched with folic acid supplementation (FDR ≤ 5%). Sorted most significant to least significant using *p*-value. Abbreviations: Sig, significant; UP, upregulated; DOWN, downregulated

| **Term** | **Total genes** | **Sig. genes** | **# UP** | **# DOWN** | ***p* – value** |
| --- | --- | --- | --- | --- | --- |
| D050813---Octamer Transcription Factor-2 | 47 | 6 | 6 | 0 | 8.35E-06 |
| D016212---Transforming Growth Factor beta | 1946 | 40 | 24 | 16 | 8.99E-06 |
| D015854---Up-Regulation | 3779 | 63 | 37 | 26 | 9.03E-06 |
| D008070---Lipopolysaccharides | 1855 | 38 | 25 | 13 | 1.79E-05 |
| D014157---Transcription Factors | 5988 | 86 | 54 | 32 | 2.08E-05 |
| D015533---Transcriptional Activation | 2951 | 52 | 32 | 20 | 2.13E-05 |
| D012334---RNA, Neoplasm | 589 | 18 | 10 | 8 | 3.59E-05 |
| D013997---Time Factors | 4671 | 71 | 42 | 29 | 4.44E-05 |
| D010012---Osteogenesis | 1229 | 28 | 16 | 12 | 4.94E-05 |
| D053823---Hedgehog Proteins | 1810 | 36 | 20 | 16 | 6.06E-05 |
| D005247---Feeding Behavior | 352 | 13 | 6 | 7 | 7.28E-05 |
| D009805---Odontogenesis | 407 | 14 | 9 | 5 | 8.17E-05 |
| D003094---Collagen | 1406 | 30 | 18 | 12 | 8.48E-05 |
| D016147---Genes, Tumor Suppressor | 1003 | 24 | 14 | 10 | 8.86E-05 |
| D051863---Host Cell Factor C1 | 102 | 7 | 5 | 2 | 8.9E-05 |
| D016335---Zinc Fingers | 1482 | 31 | 22 | 9 | 9.1E-05 |
| D005136---Eye Proteins | 1483 | 31 | 17 | 14 | 9.22E-05 |
| D051900---Smad3 Protein | 883 | 22 | 11 | 11 | 0.000101 |
| D051096---Proto-Oncogene Proteins c-ret | 364 | 13 | 8 | 5 | 0.000102 |
| D003907---Dexamethasone | 769 | 20 | 11 | 9 | 0.000123 |
| D051762---PAX2 Transcription Factor | 322 | 12 | 7 | 5 | 0.000128 |
| D006655---Histone Deacetylases | 1098 | 25 | 14 | 11 | 0.000136 |
| D006023---Glycoproteins | 1589 | 32 | 20 | 12 | 0.00014 |
| D011976---Receptors, Muscarinic | 147 | 8 | 3 | 5 | 0.000143 |
| D014409---Tumor Necrosis Factor-alpha | 1967 | 37 | 24 | 13 | 0.000151 |
| D017354---Point Mutation | 1675 | 33 | 20 | 13 | 0.000163 |
| D008868---Microtubule Proteins | 150 | 8 | 6 | 2 | 0.000164 |
| D053766---Presenilin-2 | 115 | 7 | 4 | 3 | 0.000189 |
| D020836---Protein Structure, Quaternary | 734 | 19 | 11 | 8 | 0.000196 |
| D039081---Integrin alpha5beta1 | 196 | 9 | 3 | 6 | 0.0002 |
| D034284---Dynamin I | 118 | 7 | 6 | 1 | 0.000221 |
| D002453---Cell Cycle | 2967 | 49 | 27 | 22 | 0.000235 |
| D013329---Structure-Activity Relationship | 1561 | 31 | 21 | 10 | 0.000236 |
| D020825---T-Box Domain Proteins | 940 | 22 | 12 | 10 | 0.000249 |
| D029721---Drosophila Proteins | 1495 | 30 | 19 | 11 | 0.000254 |
| D011956---Receptors, Cell Surface | 1941 | 36 | 23 | 13 | 0.000255 |
| D009363---Neoplasm Proteins | 2335 | 41 | 27 | 14 | 0.00027 |
| D000266---Adenylyl Imidodiphosphate | 31 | 4 | 3 | 1 | 0.000278 |
| D019849---Sex Determination Processes | 360 | 12 | 6 | 6 | 0.000358 |
| D014018---Tissue Distribution | 4240 | 63 | 40 | 23 | 0.000395 |
| D005938---Glucocorticoids | 652 | 17 | 9 | 8 | 0.000401 |
| D020782---Matrix Metalloproteinases | 533 | 15 | 10 | 5 | 0.000407 |
| D016601---RNA-Binding Proteins | 2550 | 43 | 24 | 19 | 0.000442 |
| D017869---Cyclic GMP-Dependent Protein Kinases | 175 | 8 | 3 | 5 | 0.000467 |
| D053773---Transforming Growth Factor beta1 | 919 | 21 | 12 | 9 | 0.00048 |
| D020125---Mutation, Missense | 1555 | 30 | 19 | 11 | 0.0005 |
| D050958---HSP47 Heat-Shock Proteins | 36 | 4 | 3 | 1 | 0.000501 |
| D050778---NFATC Transcription Factors | 991 | 22 | 11 | 11 | 0.000516 |
| D000316---Adrenergic alpha-Agonists | 98 | 6 | 2 | 4 | 0.000532 |
| D004338---Drug Combinations | 432 | 13 | 6 | 7 | 0.000539 |
| D001823---Body Composition | 379 | 12 | 6 | 6 | 0.000567 |
| D002843---Chromatin | 1869 | 34 | 19 | 15 | 0.000572 |
| D019210---Troponin I | 228 | 9 | 5 | 4 | 0.000605 |
| D029867---Xenopus Proteins | 741 | 18 | 8 | 10 | 0.000623 |
| D005712---Galvanic Skin Response | 4 | 2 | 2 | 0 | 0.00063 |
| D051153---Wnt Proteins | 1430 | 28 | 16 | 12 | 0.000634 |
| D054338---Cell Transdifferentiation | 386 | 12 | 7 | 5 | 0.000666 |
| D018124---Receptors, Tumor Necrosis Factor | 620 | 16 | 12 | 4 | 0.00067 |
| D013755---Tetradecanoylphorbol Acetate | 882 | 20 | 11 | 9 | 0.00073 |
| D003994---Bucladesine | 235 | 9 | 4 | 5 | 0.000752 |
| D002873---Chromosome Fragility | 40 | 4 | 2 | 2 | 0.000752 |
| D016693---Receptors, Antigen, T-Cell, alpha-beta | 448 | 13 | 9 | 4 | 0.000757 |
| D019655---Quantitative Trait, Heritable | 392 | 12 | 7 | 5 | 0.000763 |
| D019070---Cell Lineage | 3115 | 49 | 29 | 20 | 0.000772 |
| D005865---Gestational Age | 1375 | 27 | 14 | 13 | 0.000773 |
| D014975---Lutein | 18 | 3 | 3 | 0 | 0.000791 |
| D008970---Molecular Weight | 1531 | 29 | 23 | 6 | 0.000852 |
| D001324---Autoantigens | 829 | 19 | 12 | 7 | 0.000883 |
| D007633---Keratins | 516 | 14 | 8 | 6 | 0.000913 |
| D011499---Protein Processing, Post-Translational | 2638 | 43 | 25 | 18 | 0.000922 |
| D020123---Sirolimus | 517 | 14 | 7 | 7 | 0.00093 |
| D009435---Synaptic Transmission | 1248 | 25 | 15 | 10 | 0.000941 |
| D014212---Tretinoin | 1177 | 24 | 13 | 11 | 0.000944 |
| D046988---Proteasome Endopeptidase Complex | 1541 | 29 | 16 | 13 | 0.000945 |
| D015536---Down-Regulation | 3833 | 57 | 34 | 23 | 0.000948 |
| D004742---Enhancer Elements, Genetic | 1394 | 27 | 10 | 17 | 0.000953 |
| D043222---Ubiquitin Thiolesterase | 521 | 14 | 8 | 6 | 0.001001 |
| D019010---Vascular Cell Adhesion Molecule-1 | 462 | 13 | 8 | 5 | 0.001004 |
| D054322---tat Gene Products, Human Immunodeficiency Virus | 111 | 6 | 4 | 2 | 0.001026 |
| D002452---Cell Count | 1554 | 29 | 15 | 14 | 0.001079 |
| D051538---Hepatocyte Nuclear Factor 1-alpha | 200 | 8 | 5 | 3 | 0.00112 |
| D018168---Receptors, Retinoic Acid | 654 | 16 | 12 | 4 | 0.00118 |
| D019319---Okadaic Acid | 251 | 9 | 6 | 3 | 0.001196 |
| D047992---TNF Receptor-Associated Factor 2 | 251 | 9 | 5 | 4 | 0.001196 |
| D054377---Chemokine CXCL12 | 471 | 13 | 7 | 6 | 0.001196 |
| D008869---Microtubule-Associated Proteins | 1872 | 33 | 24 | 9 | 0.001223 |
| D009955---Ornithine Decarboxylase | 157 | 7 | 4 | 3 | 0.001227 |
| D002940---Circadian Rhythm | 657 | 16 | 6 | 10 | 0.001238 |
| D005346---Fibroblast Growth Factors | 1273 | 25 | 13 | 12 | 0.001248 |
| D013047---Specific Pathogen-Free Organisms | 788 | 18 | 9 | 9 | 0.001264 |
| D005038---Ethylnitrosourea | 534 | 14 | 8 | 6 | 0.001267 |
| D002265---Carboxylic Ester Hydrolases | 158 | 7 | 5 | 2 | 0.001273 |
| D044127---Epigenesis, Genetic | 1647 | 30 | 15 | 15 | 0.001286 |
| D010453---Peptide Synthases | 116 | 6 | 5 | 1 | 0.001289 |
| D013718---Tensile Strength | 116 | 6 | 3 | 3 | 0.001289 |
| D055751---SOXC Transcription Factors | 417 | 12 | 6 | 6 | 0.0013 |
| D016760---Proto-Oncogene Proteins c-fos | 661 | 16 | 8 | 8 | 0.001318 |
| D011347---Procollagen | 117 | 6 | 3 | 3 | 0.001347 |
| D012689---Sequence Homology, Nucleic Acid | 3194 | 49 | 33 | 16 | 0.001383 |
| D000200---Action Potentials | 665 | 16 | 9 | 7 | 0.001403 |
| D002097---C-Reactive Protein | 364 | 11 | 7 | 4 | 0.001405 |
| D011945---Receptors, Angiotensin | 119 | 6 | 2 | 4 | 0.00147 |
| D036122---Receptor, EphA4 | 162 | 7 | 4 | 3 | 0.00147 |
| D009479---Neuropeptides | 1216 | 24 | 14 | 10 | 0.001479 |
| D016373---DNA, Antisense | 209 | 8 | 4 | 4 | 0.001483 |
| D020928---Mitogen-Activated Protein Kinases | 1436 | 27 | 14 | 13 | 0.001484 |
| D002878---Chromosomes, Human, Pair 1 | 544 | 14 | 12 | 2 | 0.001509 |
| D000683---Serum Amyloid P-Component | 260 | 9 | 6 | 3 | 0.001527 |
| D005807---Genes, Overlapping | 82 | 5 | 3 | 2 | 0.001593 |
| D001343---Autophagy | 1296 | 25 | 17 | 8 | 0.001604 |
| D004365---Drugs, Chinese Herbal | 165 | 7 | 5 | 2 | 0.001634 |
| D058263---Receptor Activity-Modifying Protein 2 | 23 | 3 | 2 | 1 | 0.001652 |
| D001667---Binding, Competitive | 1013 | 21 | 10 | 11 | 0.001664 |
| D014161---Transduction, Genetic | 876 | 19 | 12 | 7 | 0.001684 |
| D002384---Catalysis | 1015 | 21 | 11 | 10 | 0.001705 |
| D004249---DNA Damage | 1525 | 28 | 19 | 9 | 0.001711 |
| D044783---F-Box Proteins | 431 | 12 | 7 | 5 | 0.001719 |
| D019521---Body Patterning | 1994 | 34 | 21 | 13 | 0.001802 |
| D004273---DNA, Neoplasm | 555 | 14 | 9 | 5 | 0.00182 |
| D018160---Receptors, Cytoplasmic and Nuclear | 1309 | 25 | 15 | 10 | 0.001842 |
| D013091---Spermatogenesis | 1534 | 28 | 19 | 9 | 0.001868 |
| D006490---Hemostatics | 51 | 4 | 1 | 3 | 0.001885 |
| D050818---Transcription Factor Brn-3 | 51 | 4 | 2 | 2 | 0.001885 |
| D020929---Mitogen-Activated Protein Kinase Kinases | 685 | 16 | 9 | 7 | 0.001901 |
| D013049---Spectrin | 170 | 7 | 5 | 2 | 0.001937 |
| D051176---beta Catenin | 1538 | 28 | 17 | 11 | 0.001942 |
| D060449---Wnt Signaling Pathway | 1026 | 21 | 11 | 10 | 0.001945 |
| D039422---Integrin alpha3 | 86 | 5 | 1 | 4 | 0.001967 |
| D004398---Dyneins | 380 | 11 | 8 | 3 | 0.001974 |
| D019012---Integrin beta1 | 624 | 15 | 7 | 8 | 0.002004 |
| D006019---Glycolysis | 825 | 18 | 8 | 10 | 0.002103 |
| D001835---Body Weight | 1547 | 28 | 14 | 14 | 0.002118 |
| D017362---Cyclic AMP Response Element-Binding Protein | 895 | 19 | 9 | 10 | 0.002151 |
| D011794---Quercetin | 88 | 5 | 4 | 1 | 0.002177 |
| D004318---Doxycycline | 386 | 11 | 5 | 6 | 0.002231 |
| D018377---Neurotransmitter Agents | 445 | 12 | 7 | 5 | 0.002242 |
| D005353---Fibronectins | 830 | 18 | 9 | 9 | 0.002246 |
| D014446---Tyrosine 3-Monooxygenase | 568 | 14 | 10 | 4 | 0.002253 |
| D018836---Inflammation Mediators | 1110 | 22 | 15 | 7 | 0.002259 |
| D010446---Peptide Fragments | 2182 | 36 | 25 | 11 | 0.002284 |
| D015973---Gene Expression Regulation, Leukemic | 446 | 12 | 7 | 5 | 0.002284 |
| D039421---Integrin alpha2 | 89 | 5 | 2 | 3 | 0.002287 |
| D018118---Chloride Channels | 331 | 10 | 5 | 5 | 0.002317 |
| D059467---Transcriptome | 1945 | 33 | 17 | 16 | 0.002327 |
| D053553---Keratin-6 | 54 | 4 | 2 | 2 | 0.002329 |
| D053605---Leukemia Inhibitory Factor | 508 | 13 | 9 | 4 | 0.002339 |
| D009431---Neural Conduction | 176 | 7 | 4 | 3 | 0.002357 |
| D020891---Raclopride | 26 | 3 | 3 | 0 | 0.002371 |
| D050988---Vesicle-Associated Membrane Protein 2 | 131 | 6 | 5 | 1 | 0.002394 |
| D005033---Ethylmaleimide | 90 | 5 | 3 | 2 | 0.002402 |
| D015739---Nocodazole | 278 | 9 | 6 | 3 | 0.002409 |
| D011518---Proto-Oncogene Proteins | 2600 | 41 | 24 | 17 | 0.002453 |
| D053496---Inositol 1,4,5-Trisphosphate Receptors | 227 | 8 | 3 | 5 | 0.002487 |
| D008027---Light | 575 | 14 | 11 | 3 | 0.00252 |
| D001896---Boron Compounds | 91 | 5 | 3 | 2 | 0.002521 |
| D009418---S100 Proteins | 335 | 10 | 6 | 4 | 0.002527 |
| D005810---Multigene Family | 3720 | 54 | 35 | 19 | 0.002579 |
| D015759---Ionomycin | 281 | 9 | 6 | 3 | 0.002589 |
| D014841---von Willebrand Factor | 229 | 8 | 3 | 5 | 0.002626 |
| D029681---Arabidopsis Proteins | 229 | 8 | 5 | 3 | 0.002626 |
| D044023---Receptor, Endothelin B | 92 | 5 | 3 | 2 | 0.002644 |
| D040121---Semaphorin-3A | 180 | 7 | 4 | 3 | 0.002673 |
| D051821---Lymphoid Enhancer-Binding Factor 1 | 338 | 10 | 4 | 6 | 0.002695 |
| D015534---Trans-Activators | 2950 | 45 | 29 | 16 | 0.002721 |
| D005314---Embryonic and Fetal Development | 1965 | 33 | 21 | 12 | 0.002752 |
| D012542---Scattering, Radiation | 93 | 5 | 4 | 1 | 0.002771 |
| D018398---Homeodomain Proteins | 3469 | 51 | 33 | 18 | 0.002781 |
| D009842---NA | 457 | 12 | 10 | 2 | 0.002789 |
| D000642---Ammonia-Lyases | 8 | 2 | 2 | 0 | 0.002861 |
| D053675---Versicans | 136 | 6 | 3 | 3 | 0.002887 |
| D005326---Fetal Proteins | 460 | 12 | 6 | 6 | 0.002942 |
| D053138---Cyclohexenes | 28 | 3 | 2 | 1 | 0.002943 |
| D058286---Calcitonin Receptor-Like Protein | 28 | 3 | 2 | 1 | 0.002943 |
| D013869---Thiolester Hydrolases | 137 | 6 | 3 | 3 | 0.002994 |
| D016384---Consensus Sequence | 922 | 19 | 10 | 9 | 0.003001 |
| D020409---Molecular Motor Proteins | 184 | 7 | 5 | 2 | 0.003022 |
| D001665---Binding Sites | 4829 | 66 | 42 | 24 | 0.003023 |
| D018396---Mucin-1 | 95 | 5 | 3 | 2 | 0.003039 |
| D020033---Protein Isoforms | 3571 | 52 | 32 | 20 | 0.003056 |
| D015513---Oncogene Proteins | 924 | 19 | 13 | 6 | 0.003074 |
| D043562---Receptors, G-Protein-Coupled | 1287 | 24 | 14 | 10 | 0.003141 |
| D011500---Protein Synthesis Inhibitors | 721 | 16 | 9 | 7 | 0.003171 |
| D013388---Succinimides | 96 | 5 | 3 | 2 | 0.00318 |
| D017466---Receptors, Endothelin | 96 | 5 | 3 | 2 | 0.00318 |
| D051524---Fibroblast Growth Factor 8 | 346 | 10 | 3 | 7 | 0.003187 |
| D050800---STAT6 Transcription Factor | 290 | 9 | 8 | 1 | 0.003194 |
| D015820---Cadherins | 1443 | 26 | 15 | 11 | 0.003328 |
| D004268---DNA-Binding Proteins | 5219 | 70 | 44 | 26 | 0.003347 |
| D019205---GTP-Binding Protein alpha Subunits, Gs | 188 | 7 | 2 | 5 | 0.003404 |
| D051539---Hepatocyte Nuclear Factor 1-beta | 141 | 6 | 2 | 4 | 0.003452 |
| D003545---Cysteine | 864 | 18 | 12 | 6 | 0.003456 |
| D007797---Laminin | 797 | 17 | 10 | 7 | 0.00354 |
| D051057---Proto-Oncogene Proteins c-akt | 2158 | 35 | 19 | 16 | 0.00357 |
| D055293---Receptors, Urokinase Plasminogen Activator | 142 | 6 | 4 | 2 | 0.003574 |
| D014268---Trifluoperazine | 30 | 3 | 0 | 3 | 0.003593 |
| D051340---Cofilin 2 | 30 | 3 | 0 | 3 | 0.003593 |
| D064446---Orexin Receptors | 30 | 3 | 1 | 2 | 0.003593 |
| D051840---Y-Box-Binding Protein 1 | 99 | 5 | 2 | 3 | 0.003631 |
| D015850---Interleukin-6 | 1302 | 24 | 14 | 10 | 0.003645 |
| D018807---Polymorphism, Single-Stranded Conformational | 537 | 13 | 8 | 5 | 0.003765 |
| D018342---Receptors, Adrenergic, beta-1 | 100 | 5 | 1 | 4 | 0.003791 |
| D016213---Cyclins | 735 | 16 | 7 | 9 | 0.003826 |
| D000423---Alcian Blue | 62 | 4 | 2 | 2 | 0.003855 |
| D008213---Lymphocyte Activation | 1770 | 30 | 18 | 12 | 0.003905 |
| D014704---Veratrum Alkaloids | 101 | 5 | 2 | 3 | 0.003956 |
| D018840---HSP70 Heat-Shock Proteins | 541 | 13 | 10 | 3 | 0.004008 |
| D015415---Biomarkers | 2669 | 41 | 21 | 20 | 0.004018 |
| D048049---Extracellular Signal-Regulated MAP Kinases | 1392 | 25 | 13 | 12 | 0.0042 |
| D006720---Homozygote | 2261 | 36 | 22 | 14 | 0.004204 |
| D009024---NA | 1095 | 21 | 11 | 10 | 0.004216 |
| D009419---Nerve Tissue Proteins | 5262 | 70 | 43 | 27 | 0.004245 |
| D002465---Cell Movement | 3362 | 49 | 28 | 21 | 0.004266 |
| D001285---Atropine | 64 | 4 | 1 | 3 | 0.004321 |
| D015087---2',3'-Cyclic-Nucleotide Phosphodiesterases | 64 | 4 | 4 | 0 | 0.004321 |
| D039423---Integrin alpha1 | 64 | 4 | 2 | 2 | 0.004321 |
| D053379---Chromogranin A | 64 | 4 | 2 | 2 | 0.004321 |
| D048430---Cell Shape | 1098 | 21 | 11 | 10 | 0.004351 |
| D014443---Tyrosine | 1398 | 25 | 15 | 10 | 0.004441 |
| D006706---Homeostasis | 2602 | 40 | 20 | 20 | 0.004513 |
| D019797---3-Iodobenzylguanidine | 10 | 2 | 2 | 0 | 0.004535 |
| D019805---alpha-Methyltyrosine | 10 | 2 | 2 | 0 | 0.004535 |
| D002374---Catalase | 306 | 9 | 5 | 4 | 0.004541 |
| D001054---Apolipoproteins A | 65 | 4 | 2 | 2 | 0.004568 |
| D002352---Carrier Proteins | 4903 | 66 | 46 | 20 | 0.004587 |
| D058750---Epithelial-Mesenchymal Transition | 749 | 16 | 8 | 8 | 0.004589 |
| D019408---Platelet Endothelial Cell Adhesion Molecule-1 | 487 | 12 | 6 | 6 | 0.004644 |
| D051545---Cyclooxygenase 1 | 150 | 6 | 4 | 2 | 0.004671 |
| D008024---NA | 1480 | 26 | 13 | 13 | 0.004675 |
| D003598---Cytoskeletal Proteins | 1952 | 32 | 22 | 10 | 0.004711 |
| D029961---Zebrafish Proteins | 1033 | 20 | 11 | 9 | 0.004719 |
| D001464---NA | 33 | 3 | 1 | 2 | 0.00472 |
| D051237---Receptors, Pituitary Adenylate Cyclase-Activating Polypeptide, Type I | 33 | 3 | 2 | 1 | 0.00472 |
| D011509---Proteoglycans | 891 | 18 | 9 | 9 | 0.004768 |
| D011973---Receptors, LDL | 821 | 17 | 8 | 9 | 0.004769 |
| D002455---Cell Division | 2779 | 42 | 24 | 18 | 0.004787 |
| D005680---gamma-Aminobutyric Acid | 553 | 13 | 9 | 4 | 0.004814 |
| D017930---Genes, Reporter | 2950 | 44 | 26 | 18 | 0.004818 |
| D025521---Tumor Suppressor Proteins | 2528 | 39 | 23 | 16 | 0.004822 |
| D017473---Receptors, Opioid, kappa | 66 | 4 | 1 | 3 | 0.004825 |
| D023083---beta-Defensins | 66 | 4 | 4 | 0 | 0.004825 |
| D016315---Proto-Oncogene Proteins c-abl | 255 | 8 | 5 | 3 | 0.005031 |
| D054756---Cyclic AMP-Dependent Protein Kinase RIalpha Subunit | 67 | 4 | 2 | 2 | 0.005091 |
| D035581---Transcription Factor TFIIB | 34 | 3 | 2 | 1 | 0.005138 |
| D012084---Renin-Angiotensin System | 203 | 7 | 3 | 4 | 0.005174 |
| D000956---Antigens, Viral | 371 | 10 | 4 | 6 | 0.0052 |
| D036341---Intercellular Signaling Peptides and Proteins | 1965 | 32 | 19 | 13 | 0.005212 |
| D018492---Dopamine Antagonists | 108 | 5 | 4 | 1 | 0.005257 |
| D044168---Receptor, Parathyroid Hormone, Type 1 | 108 | 5 | 3 | 2 | 0.005257 |
| D011905---Genes, ras | 626 | 14 | 9 | 5 | 0.005363 |
| D002892---Chromosomes, Human, Pair 22 | 258 | 8 | 3 | 5 | 0.005392 |
| D034741---RNA, Small Interfering | 4201 | 58 | 34 | 24 | 0.005425 |
| D009928---Organ Specificity | 10025 | 116 | 69 | 47 | 0.005442 |
| D002619---Chemical Warfare Agents | 11 | 2 | 2 | 0 | 0.005506 |
| D056325---Neuronal Tract-Tracers | 11 | 2 | 2 | 0 | 0.005506 |
| D004801---Eosine Yellowish-(YS) | 35 | 3 | 2 | 1 | 0.005578 |
| D019653---Myeloablative Agonists | 35 | 3 | 2 | 1 | 0.005578 |
| D053552---Keratin-7 | 35 | 3 | 1 | 2 | 0.005578 |
| D057167---Claudins | 110 | 5 | 2 | 3 | 0.005679 |
| D012701---Serotonin | 500 | 12 | 7 | 5 | 0.005707 |
| D011503---Transglutaminases | 207 | 7 | 5 | 2 | 0.005745 |
| D022763---CCAAT-Enhancer-Binding Protein-alpha | 318 | 9 | 6 | 3 | 0.005814 |
| D054884---Host-Pathogen Interactions | 700 | 15 | 9 | 6 | 0.005867 |
| D018006---Myogenic Regulatory Factors | 1506 | 26 | 15 | 11 | 0.005876 |
| D019898---Autocrine Communication | 567 | 13 | 8 | 5 | 0.005915 |
| D004317---Doxorubicin | 503 | 12 | 8 | 4 | 0.005978 |
| D000728---Androgens | 379 | 10 | 6 | 4 | 0.006021 |
| D053531---Keratins, Type II | 36 | 3 | 2 | 1 | 0.006039 |
| D053221---TNF-Related Apoptosis-Inducing Ligand | 209 | 7 | 2 | 5 | 0.006048 |
| D056284---Histone Deacetylase 1 | 320 | 9 | 4 | 5 | 0.006051 |
| D009218---Myosins | 504 | 12 | 7 | 5 | 0.00607 |
| D019009---Proto-Oncogene Proteins c-kit | 504 | 12 | 8 | 4 | 0.00607 |
| D039943---Neuropilin-2 | 112 | 5 | 2 | 3 | 0.006123 |
| D001831---Body Temperature | 264 | 8 | 4 | 4 | 0.006172 |
| D009599---Nitroprusside | 159 | 6 | 3 | 3 | 0.006183 |
| D005656---Fungal Proteins | 845 | 17 | 11 | 6 | 0.006332 |
| D011493---Protein Kinase C | 1361 | 24 | 12 | 12 | 0.006349 |
| D018679---Cholinergic Agonists | 113 | 5 | 1 | 4 | 0.006355 |
| D047428---Protein Kinase Inhibitors | 1210 | 22 | 14 | 8 | 0.006368 |
| D003513---Cycloheximide | 573 | 13 | 6 | 7 | 0.006444 |
| D019063---Tenascin | 266 | 8 | 5 | 3 | 0.00645 |
| D019899---Paracrine Communication | 640 | 14 | 9 | 5 | 0.006485 |
| D018959---CD57 Antigens | 37 | 3 | 2 | 1 | 0.006523 |
| D051400---Aquaporin 3 | 37 | 3 | 2 | 1 | 0.006523 |
| D019001---Nitric Oxide Synthase | 574 | 13 | 6 | 7 | 0.006536 |
| D015687---Cell Hypoxia | 919 | 18 | 11 | 7 | 0.006537 |
| D009281---NA | 161 | 6 | 3 | 3 | 0.006562 |
| D050486---Serotonin Plasma Membrane Transport Proteins | 161 | 6 | 3 | 3 | 0.006562 |
| D006510---Hepatitis B Antibodies | 12 | 2 | 1 | 1 | 0.006562 |
| D013830---NA | 12 | 2 | 2 | 0 | 0.006562 |
| D050884---HSP72 Heat-Shock Proteins | 72 | 4 | 4 | 0 | 0.006568 |
| D051776---Early Growth Response Protein 3 | 72 | 4 | 3 | 1 | 0.006568 |
| D008431---Maternal-Fetal Exchange | 267 | 8 | 5 | 3 | 0.006593 |
| D004298---Dopamine | 779 | 16 | 9 | 7 | 0.006648 |
| D011948---Receptors, Antigen, T-Cell | 995 | 19 | 11 | 8 | 0.006806 |
| D001760---Bleeding Time | 115 | 5 | 2 | 3 | 0.006836 |
| D036801---Parturition | 115 | 5 | 2 | 3 | 0.006836 |
| D018975---Insulin-Like Growth Factor Binding Protein 5 | 73 | 4 | 1 | 3 | 0.006894 |
| D055605---NA | 73 | 4 | 1 | 3 | 0.006894 |
| D025461---Feedback, Physiological | 925 | 18 | 9 | 9 | 0.006979 |
| D001426---Bacterial Proteins | 926 | 18 | 10 | 8 | 0.007055 |
| D013844---NA | 328 | 9 | 2 | 7 | 0.007074 |
| D050811---Octamer Transcription Factor-1 | 116 | 5 | 5 | 0 | 0.007086 |
| D010766---Phosphorylation | 4892 | 65 | 37 | 28 | 0.007113 |
| D000431---Ethanol | 649 | 14 | 10 | 4 | 0.007302 |
| D053583---Interleukin-1beta | 858 | 17 | 14 | 3 | 0.007339 |
| D015682---Retroviridae Proteins, Oncogenic | 165 | 6 | 4 | 2 | 0.007371 |
| D056950---Period Circadian Proteins | 217 | 7 | 3 | 4 | 0.007381 |
| D050783---Proto-Oncogene Proteins c-ets | 454 | 11 | 6 | 5 | 0.007512 |
| D007671---Kidney Concentrating Ability | 75 | 4 | 2 | 2 | 0.007577 |
| D016880---Anisotropy | 75 | 4 | 2 | 2 | 0.007577 |
| D026422---S-Nitrosoglutathione | 75 | 4 | 3 | 1 | 0.007577 |
| D007333---Insulin Resistance | 1229 | 22 | 11 | 11 | 0.007612 |
| D000931---Antidotes | 13 | 2 | 2 | 0 | 0.007703 |
| D002569---Cerulenin | 13 | 2 | 1 | 1 | 0.007703 |
| D008768---NA | 13 | 2 | 2 | 0 | 0.007703 |
| D042787---Receptor, TIE-2 | 333 | 9 | 5 | 4 | 0.007777 |
| D000255---Adenosine Triphosphate | 1384 | 24 | 14 | 10 | 0.00778 |
| D038041---NA | 167 | 6 | 4 | 2 | 0.007801 |
| D054648---Protein Phosphatase 2 | 457 | 11 | 7 | 4 | 0.007875 |
| D018727---Muscarinic Antagonists | 76 | 4 | 1 | 3 | 0.007935 |
| D007157---Immunologic Surveillance | 168 | 6 | 3 | 3 | 0.008023 |
| D019006---Neural Cell Adhesion Molecules | 336 | 9 | 7 | 2 | 0.008224 |
| D016244---Guanosine 5'-O-(3-Thiotriphosphate) | 169 | 6 | 1 | 5 | 0.00825 |
| D011508---Chondroitin Sulfate Proteoglycans | 222 | 7 | 3 | 4 | 0.008317 |
| D020868---Gene Silencing | 2193 | 34 | 18 | 16 | 0.008381 |
| D010064---Embryo Implantation | 729 | 15 | 10 | 5 | 0.008411 |
| D005856---Germ-Free Life | 121 | 5 | 3 | 2 | 0.008432 |
| D044139---Receptor, Angiotensin, Type 2 | 121 | 5 | 1 | 4 | 0.008432 |
| D049294---Phospholipid Transfer Proteins | 170 | 6 | 4 | 2 | 0.008481 |
| D006487---Hemostasis | 223 | 7 | 4 | 3 | 0.008514 |
| D011988---Receptors, Thyroid Hormone | 338 | 9 | 7 | 2 | 0.008533 |
| D013045---Species Specificity | 3129 | 45 | 26 | 19 | 0.008585 |
| D015500---Chloramphenicol O-Acetyltransferase | 400 | 10 | 5 | 5 | 0.008662 |
| D009185---Myelin Proteins | 339 | 9 | 7 | 2 | 0.00869 |
| D060850---LIM-Homeodomain Proteins | 732 | 15 | 10 | 5 | 0.008718 |
| D051792---Basic Helix-Loop-Helix Transcription Factors | 1712 | 28 | 13 | 15 | 0.008753 |
| D011374---Progesterone | 530 | 12 | 5 | 7 | 0.008904 |
| D008466---Mechlorethamine | 14 | 2 | 2 | 0 | 0.008927 |
| D019869---Phosphatidylinositol 3-Kinases | 1795 | 29 | 15 | 14 | 0.008954 |
| D001205---Ascorbic Acid | 172 | 6 | 3 | 3 | 0.008956 |
| D019927---NA | 172 | 6 | 5 | 1 | 0.008956 |
| D040321---Vascular Endothelial Growth Factor Receptor-3 | 172 | 6 | 2 | 4 | 0.008956 |
| D060466---Axin Protein | 282 | 8 | 5 | 3 | 0.009033 |
| D012097---Repressor Proteins | 3754 | 52 | 33 | 19 | 0.009035 |
| D015514---Oncogene Proteins, Fusion | 466 | 11 | 6 | 5 | 0.009046 |
| D002317---Cardiovascular Agents | 79 | 4 | 1 | 3 | 0.009076 |
| D012259---Ribonuclease, Pancreatic | 79 | 4 | 4 | 0 | 0.009076 |
| D018013---Receptors, Neuropeptide | 79 | 4 | 3 | 1 | 0.009076 |
| D051496---Receptor, Fibroblast Growth Factor, Type 1 | 342 | 9 | 5 | 4 | 0.009177 |
| D049109---Cell Proliferation | 4943 | 65 | 38 | 27 | 0.009248 |
| D008623---Mercaptoethanol | 42 | 3 | 3 | 0 | 0.009283 |
| D011809---NA | 124 | 5 | 4 | 1 | 0.009319 |
| D019253---Proto-Oncogene Proteins c-bcl-2 | 1026 | 19 | 11 | 8 | 0.009327 |
| D002918---Chymotrypsin | 80 | 4 | 2 | 2 | 0.009478 |
| D019002---CD56 Antigen | 80 | 4 | 0 | 4 | 0.009478 |
| D056404---Chaperonin Containing TCP-1 | 80 | 4 | 3 | 1 | 0.009478 |
| D020218---Response Elements | 1028 | 19 | 10 | 9 | 0.009512 |
| D055372---AMP-Activated Protein Kinases | 670 | 14 | 9 | 5 | 0.009527 |
| D051744---Protein Kinase C-epsilon | 228 | 7 | 2 | 5 | 0.009553 |
| D020440---Gene Duplication | 407 | 10 | 5 | 5 | 0.009717 |
| D019175---DNA Methylation | 1411 | 24 | 9 | 15 | 0.009792 |
| D002135---Calcium-Binding Proteins | 1807 | 29 | 18 | 11 | 0.009793 |
| D016329---Sp1 Transcription Factor | 604 | 13 | 8 | 5 | 0.009815 |
| D013739---Testosterone | 472 | 11 | 5 | 6 | 0.009899 |
| D018833---Chaperonins | 126 | 5 | 4 | 1 | 0.009945 |
| D049368---Receptors, Lysophosphatidic Acid | 126 | 5 | 2 | 3 | 0.009945 |
| D050257---Tubulin Modulators | 126 | 5 | 3 | 2 | 0.009945 |
| D051219---Pituitary Adenylate Cyclase-Activating Polypeptide | 126 | 5 | 4 | 1 | 0.009945 |
| D051757---PAX5 Transcription Factor | 126 | 5 | 4 | 1 | 0.009945 |
| D056931---Cryptochromes | 126 | 5 | 0 | 5 | 0.009945 |
| D053245---RANK Ligand | 675 | 14 | 8 | 6 | 0.010129 |
| D004815---Epidermal Growth Factor | 961 | 18 | 9 | 9 | 0.010169 |
| D020134---Catalytic Domain | 961 | 18 | 11 | 7 | 0.010169 |
| D016704---Synapsins | 177 | 6 | 2 | 4 | 0.010229 |
| D064307---Microbiota | 177 | 6 | 2 | 4 | 0.010229 |
| D017998---Leukotriene D4 | 15 | 2 | 2 | 0 | 0.010231 |
| D020111---Chlorodiphenyl (54% Chlorine) | 15 | 2 | 2 | 0 | 0.010231 |
| D000957---Antigens, Viral, Tumor | 127 | 5 | 3 | 2 | 0.010269 |
| D016376---Oligonucleotides, Antisense | 889 | 17 | 9 | 8 | 0.01027 |
| D007700---NA | 2059 | 32 | 20 | 12 | 0.010362 |
| D050814---Octamer Transcription Factor-3 | 890 | 17 | 11 | 6 | 0.010378 |
| D009414---Nerve Growth Factors | 747 | 15 | 10 | 5 | 0.01039 |
| D051858---Forkhead Transcription Factors | 1896 | 30 | 17 | 13 | 0.01039 |
| D015815---Cell Adhesion Molecules | 1497 | 25 | 11 | 14 | 0.010442 |
| D011971---Receptors, Immunologic | 891 | 17 | 10 | 7 | 0.010487 |
| D047629---Estrogen Receptor beta | 178 | 6 | 3 | 3 | 0.010498 |
| D015398---Signal Transduction | 7389 | 90 | 55 | 35 | 0.010645 |
| D009043---Motor Activity | 1267 | 22 | 13 | 9 | 0.010705 |
| D017951---Antigen Presentation | 611 | 13 | 8 | 5 | 0.01074 |
| D002118---Calcium | 2481 | 37 | 23 | 14 | 0.010753 |
| D011720---NA | 179 | 6 | 5 | 1 | 0.010773 |
| D018797---Cell Cycle Proteins | 3081 | 44 | 25 | 19 | 0.010781 |
| D019746---Intramolecular Oxidoreductases | 291 | 8 | 4 | 4 | 0.01079 |
| D011487---Protein Conformation | 2482 | 37 | 25 | 12 | 0.010821 |
| D002899---Chromosomes, Human, Pair 9 | 234 | 7 | 5 | 2 | 0.010919 |
| D016877---Oxidants | 415 | 10 | 6 | 4 | 0.01104 |
| D005456---Fluorescent Dyes | 896 | 17 | 11 | 6 | 0.011047 |
| D007213---Indomethacin | 180 | 6 | 4 | 2 | 0.011052 |
| D016326---Extracellular Matrix Proteins | 1743 | 28 | 19 | 9 | 0.011065 |
| D055550---Protein Stability | 1195 | 21 | 15 | 6 | 0.01113 |
| D020738---Leptin | 614 | 13 | 8 | 5 | 0.011156 |
| D001777---Blood Coagulation | 235 | 7 | 5 | 2 | 0.01116 |
| D047630---Depsipeptides | 84 | 4 | 0 | 4 | 0.011204 |
| D050820---Transcription Factor Brn-3B | 84 | 4 | 3 | 1 | 0.011204 |
| D018016---Receptors, Parathyroid Hormone | 45 | 3 | 1 | 2 | 0.01122 |
| D020717---Eukaryotic Initiation Factor-2B | 45 | 3 | 1 | 2 | 0.01122 |
| D039482---Integrin alpha5 | 130 | 5 | 1 | 4 | 0.011283 |
| D013820---Thermolysin | 16 | 2 | 1 | 1 | 0.011613 |
| D058987---Phosphodiesterase 3 Inhibitors | 16 | 2 | 0 | 2 | 0.011613 |
| D061209---Proton Ionophores | 16 | 2 | 2 | 0 | 0.011613 |
| D015395---Histocompatibility Antigens Class I | 419 | 10 | 4 | 6 | 0.011751 |
| D008666---Metalloendopeptidases | 619 | 13 | 7 | 6 | 0.011879 |
| D050784---Proto-Oncogene Protein c-ets-1 | 238 | 7 | 4 | 3 | 0.011905 |
| D050989---GATA2 Transcription Factor | 238 | 7 | 4 | 3 | 0.011905 |
| D011673---Pulsatile Flow | 46 | 3 | 1 | 2 | 0.011913 |
| D002791---Cholesterol, Dietary | 183 | 6 | 3 | 3 | 0.01192 |
| D048008---TNF Receptor-Associated Factor 3 | 132 | 5 | 3 | 2 | 0.011996 |
| D002784---Cholesterol | 1128 | 20 | 10 | 10 | 0.01206 |
| D003285---Contractile Proteins | 297 | 8 | 3 | 5 | 0.012094 |
| D011829---Radiation Dosage | 86 | 4 | 4 | 0 | 0.012139 |
| D051793---Hypoxia-Inducible Factor 1 | 239 | 7 | 3 | 4 | 0.012161 |
| D013696---Temperature | 761 | 15 | 11 | 4 | 0.01217 |
| D002147---Calmodulin | 621 | 13 | 8 | 5 | 0.012178 |
| D025542---Neurofibromin 1 | 184 | 6 | 2 | 4 | 0.01222 |
| D009866---Oogenesis | 422 | 10 | 6 | 4 | 0.012306 |
| D017475---Receptors, Nerve Growth Factor | 298 | 8 | 4 | 4 | 0.012322 |
| D048052---Mitogen-Activated Protein Kinase 3 | 1057 | 19 | 10 | 9 | 0.012556 |
| D017384---Sequence Deletion | 2005 | 31 | 15 | 16 | 0.012575 |
| D017766---Acid Anhydride Hydrolases | 47 | 3 | 3 | 0 | 0.012631 |
| D050722---Oncogene Protein v-cbl | 47 | 3 | 2 | 1 | 0.012631 |
| D016158---Genes, p53 | 556 | 12 | 8 | 4 | 0.012684 |
| D002864---Chromogranins | 134 | 5 | 2 | 3 | 0.012739 |
| D020935---MAP Kinase Signaling System | 2007 | 31 | 19 | 12 | 0.012747 |
| D009320---Atrial Natriuretic Factor | 242 | 7 | 2 | 5 | 0.012954 |
| D020848---Chimerin 1 | 17 | 2 | 2 | 0 | 0.013073 |
| D053304---Apolipoprotein C-II | 17 | 2 | 1 | 1 | 0.013073 |
| D002787---Sterol Esterase | 135 | 5 | 3 | 2 | 0.013122 |
| D016627---Oxidopamine | 135 | 5 | 4 | 1 | 0.013122 |
| D017089---Ankyrin Repeat | 135 | 5 | 1 | 4 | 0.013122 |
| D038203---alpha-Crystallin B Chain | 135 | 5 | 5 | 0 | 0.013122 |
| D048948---14-3-3 Proteins | 768 | 15 | 13 | 2 | 0.013146 |
| D002472---Cell Transformation, Viral | 363 | 9 | 4 | 5 | 0.013181 |
| D020744---rac GTP-Binding Proteins | 628 | 13 | 8 | 5 | 0.01327 |
| D008564---Membrane Potentials | 841 | 16 | 11 | 5 | 0.013275 |
| D019014---fas Receptor | 560 | 12 | 9 | 3 | 0.013361 |
| D001552---NA | 48 | 3 | 3 | 0 | 0.013373 |
| D019950---Mitogen-Activated Protein Kinase 1 | 1064 | 19 | 10 | 9 | 0.013396 |
| D057093---Orphan Nuclear Receptors | 244 | 7 | 3 | 4 | 0.013503 |
| D019332---Endothelin-1 | 303 | 8 | 3 | 5 | 0.013511 |
| D017405---Herpes Simplex Virus Protein Vmw65 | 136 | 5 | 2 | 3 | 0.013513 |
| D008938---Mitosis | 2099 | 32 | 18 | 14 | 0.013579 |
| D018841---HSP90 Heat-Shock Proteins | 429 | 10 | 7 | 3 | 0.01368 |
| D001057---Apolipoproteins E | 844 | 16 | 11 | 5 | 0.013694 |
| D057895---Haploinsufficiency | 701 | 14 | 9 | 5 | 0.013759 |
| D018179---Receptors, Thrombin | 137 | 5 | 2 | 3 | 0.013911 |
| D000107---Acetylation | 1298 | 22 | 13 | 9 | 0.013924 |
| D047628---Estrogen Receptor alpha | 431 | 10 | 6 | 4 | 0.014092 |
| D009856---Oncogene Proteins, Viral | 190 | 6 | 4 | 2 | 0.014133 |
| D013752---Tetracycline | 190 | 6 | 4 | 2 | 0.014133 |
| D003510---NA | 49 | 3 | 3 | 0 | 0.014139 |
| D011753---NA | 49 | 3 | 2 | 1 | 0.014139 |
| D020832---EF Hand Motifs | 49 | 3 | 3 | 0 | 0.014139 |
| D026403---S-Nitrosothiols | 49 | 3 | 2 | 1 | 0.014139 |
| D053220---Receptors, TNF-Related Apoptosis-Inducing Ligand | 90 | 4 | 2 | 2 | 0.014153 |
| D020574---Proto-Oncogene Proteins c-sis | 306 | 8 | 3 | 5 | 0.014264 |
| D016328---NF-kappa B | 2027 | 31 | 20 | 11 | 0.01458 |
| D017504---Adenovirus E4 Proteins | 18 | 2 | 1 | 1 | 0.014609 |
| D018721---Muscarinic Agonists | 91 | 4 | 1 | 3 | 0.014688 |
| D040502---Calgranulin B | 91 | 4 | 3 | 1 | 0.014688 |
| D050050---Protein Carbonylation | 91 | 4 | 3 | 1 | 0.014688 |
| D016173---Macrophage Colony-Stimulating Factor | 500 | 11 | 8 | 3 | 0.014728 |
| D009569---Nitric Oxide | 1000 | 18 | 10 | 8 | 0.014851 |
| D010750---NA | 1866 | 29 | 18 | 11 | 0.014921 |
| D036141---Receptor, EphA7 | 50 | 3 | 2 | 1 | 0.01493 |
| D020962---Heterotrimeric GTP-Binding Proteins | 249 | 7 | 3 | 4 | 0.014948 |
| D018932---Chemokine CCL2 | 638 | 13 | 8 | 5 | 0.014961 |
| D042702---Angiopoietin-2 | 140 | 5 | 2 | 3 | 0.015154 |
| D009478---Neuropeptide Y | 193 | 6 | 1 | 5 | 0.015163 |
| D034521---Nuclear Matrix-Associated Proteins | 193 | 6 | 3 | 3 | 0.015163 |
| D038681---Follistatin | 193 | 6 | 2 | 4 | 0.015163 |
| D016232---Endothelins | 92 | 4 | 0 | 4 | 0.015236 |
| D010940---Plant Proteins | 310 | 8 | 5 | 3 | 0.015315 |
| D015220---Calcium Channels | 640 | 13 | 9 | 4 | 0.015319 |
| D008562---Membrane Glycoproteins | 2286 | 34 | 22 | 12 | 0.015443 |
| D044843---SKP Cullin F-Box Protein Ligases | 373 | 9 | 6 | 3 | 0.015491 |
| D015544---Inositol 1,4,5-Trisphosphate | 194 | 6 | 2 | 4 | 0.015518 |
| D055419---Bone Morphogenetic Protein 7 | 311 | 8 | 3 | 5 | 0.015586 |
| D016193---G1 Phase | 642 | 13 | 7 | 6 | 0.015682 |
| D043244---Ribonuclease III | 374 | 9 | 4 | 5 | 0.015737 |
| D049312---Receptors, Lysophospholipid | 51 | 3 | 1 | 2 | 0.015745 |
| D062085---RNA, Long Noncoding | 785 | 15 | 7 | 8 | 0.015773 |
| D004299---Dopamine beta-Hydroxylase | 93 | 4 | 3 | 1 | 0.015796 |
| D018000---Receptors, Peptide | 252 | 7 | 5 | 2 | 0.015867 |
| D005987---Glyceraldehyde-3-Phosphate Dehydrogenases | 195 | 6 | 5 | 1 | 0.015879 |
| D050759---Cyclin-Dependent Kinase Inhibitor p21 | 859 | 16 | 8 | 8 | 0.01595 |
| D015972---Gene Expression Regulation, Neoplastic | 2717 | 39 | 22 | 17 | 0.015987 |
| D008051---Lipid Bilayers | 142 | 5 | 5 | 0 | 0.016022 |
| D000858---Anovulation | 19 | 2 | 1 | 1 | 0.016218 |
| D065128---Endothelin Receptor Antagonists | 19 | 2 | 1 | 1 | 0.016218 |
| D009638---Norepinephrine | 376 | 9 | 3 | 6 | 0.016238 |
| D004265---DNA Helicases | 935 | 17 | 11 | 6 | 0.016273 |
| D047495---PPAR gamma | 1086 | 19 | 10 | 9 | 0.016333 |
| D042002---Chromatin Assembly and Disassembly | 789 | 15 | 9 | 6 | 0.016446 |
| D012794---Sialic Acids | 143 | 5 | 1 | 4 | 0.016469 |
| D010713---Phosphatidylcholines | 254 | 7 | 3 | 4 | 0.016501 |
| D024161---Collagen Type XI | 52 | 3 | 2 | 1 | 0.016585 |
| D037521---Virulence Factors | 197 | 6 | 3 | 3 | 0.016617 |
| D004032---Diet | 864 | 16 | 10 | 6 | 0.016763 |
| D019518---Postprandial Period | 95 | 4 | 2 | 2 | 0.016955 |
| D059305---Diet, High-Fat | 1167 | 20 | 10 | 10 | 0.01697 |
| D017447---Receptors, Dopamine D1 | 198 | 6 | 4 | 2 | 0.016995 |
| D000375---Aging | 2557 | 37 | 22 | 15 | 0.017085 |
| D017209---Apoptosis | 4424 | 58 | 34 | 24 | 0.017323 |
| D007519---Isoantigens | 199 | 6 | 5 | 1 | 0.017379 |
| D051938---Munc18 Proteins | 145 | 5 | 1 | 4 | 0.017386 |
| D013152---Splanchnic Circulation | 53 | 3 | 0 | 3 | 0.01745 |
| D015088---2',5'-Oligoadenylate Synthetase | 53 | 3 | 3 | 0 | 0.01745 |
| D005904---Glial Fibrillary Acidic Protein | 795 | 15 | 11 | 4 | 0.017498 |
| D014110---Touch | 96 | 4 | 3 | 1 | 0.017554 |
| D016337---RNA, Catalytic | 96 | 4 | 4 | 0 | 0.017554 |
| D051937---Dopamine and cAMP-Regulated Phosphoprotein 32 | 96 | 4 | 3 | 1 | 0.017554 |
| D048055---Mitogen-Activated Protein Kinase 8 | 318 | 8 | 6 | 2 | 0.017583 |
| D051357---Cyclin-Dependent Kinase 2 | 318 | 8 | 3 | 5 | 0.017583 |
| D053263---Gene Regulatory Networks | 2995 | 42 | 29 | 13 | 0.017697 |
| D010587---Phagocytosis | 944 | 17 | 13 | 4 | 0.017718 |
| D056464---Histone Deacetylase 2 | 258 | 7 | 3 | 4 | 0.017823 |
| D051541---Hepatocyte Nuclear Factor 3-alpha | 146 | 5 | 2 | 3 | 0.017858 |
| D005467---Floxuridine | 20 | 2 | 1 | 1 | 0.017899 |
| D015060---1,2-Dipalmitoylphosphatidylcholine | 20 | 2 | 2 | 0 | 0.017899 |
| D051542---Hepatocyte Nuclear Factor 3-beta | 654 | 13 | 7 | 6 | 0.018006 |
| D002450---Cell Communication | 1252 | 21 | 13 | 8 | 0.018058 |
| D002454---Cell Differentiation | 6321 | 78 | 52 | 26 | 0.018081 |
| D001216---Asparagine | 201 | 6 | 4 | 2 | 0.018165 |
| D015632---1-Methyl-4-phenyl-1,2,3,6-tetrahydropyridine | 201 | 6 | 4 | 2 | 0.018165 |
| D002448---Cell Adhesion | 2061 | 31 | 20 | 11 | 0.018188 |
| D012717---Sesquiterpenes | 147 | 5 | 5 | 0 | 0.018337 |
| D014333---Tropoelastin | 54 | 3 | 0 | 3 | 0.018339 |
| D014654---Vascular Patency | 54 | 3 | 1 | 2 | 0.018339 |
| D019309---Epstein-Barr Virus Nuclear Antigens | 54 | 3 | 1 | 2 | 0.018339 |
| D000894---Anti-Inflammatory Agents, Non-Steroidal | 321 | 8 | 7 | 1 | 0.018494 |
| D010718---Phosphatidylserines | 260 | 7 | 5 | 2 | 0.018511 |
| D000252---Calcium-Transporting ATPases | 202 | 6 | 1 | 5 | 0.018567 |
| D053778---NA | 98 | 4 | 1 | 3 | 0.018791 |
| D000123---NA | 322 | 8 | 5 | 3 | 0.018804 |
| D004305---Dose-Response Relationship, Drug | 2319 | 34 | 20 | 14 | 0.018901 |
| D018925---Chemokines | 803 | 15 | 10 | 5 | 0.01898 |
| D000727---Androgen-Binding Protein | 55 | 3 | 2 | 1 | 0.019253 |
| D018598---Minisatellite Repeats | 55 | 3 | 2 | 1 | 0.019253 |
| D020856---AT Rich Sequence | 55 | 3 | 1 | 2 | 0.019253 |
| D001786---Blood Glucose | 1029 | 18 | 10 | 8 | 0.019328 |
| D024642---Potassium Channels, Voltage-Gated | 204 | 6 | 3 | 3 | 0.019388 |
| D054460---rho-Associated Kinases | 521 | 11 | 4 | 7 | 0.019389 |
| D002614---Chelating Agents | 324 | 8 | 3 | 5 | 0.019437 |
| D000109---Acetylcholine | 388 | 9 | 5 | 4 | 0.019498 |
| D051761---Paired Box Transcription Factors | 1030 | 18 | 11 | 7 | 0.019499 |
| D053781---Transforming Growth Factor beta2 | 263 | 7 | 4 | 3 | 0.019578 |
| D000662---NA | 21 | 2 | 2 | 0 | 0.01965 |
| D005464---NA | 21 | 2 | 1 | 1 | 0.01965 |
| D010935---Plant Diseases | 21 | 2 | 2 | 0 | 0.01965 |
| D024745---Smooth Muscle Myosins | 21 | 2 | 0 | 2 | 0.01965 |
| D024510---Muscle Development | 2157 | 32 | 18 | 14 | 0.01966 |
| D050982---GATA1 Transcription Factor | 325 | 8 | 4 | 4 | 0.019759 |
| D002875---Chromosomes | 1826 | 28 | 16 | 12 | 0.019813 |
| D011994---Recombinant Proteins | 4087 | 54 | 31 | 23 | 0.019842 |
| D011993---Recombinant Fusion Proteins | 4088 | 54 | 34 | 20 | 0.019938 |
| D004789---Enzyme Activation | 2931 | 41 | 22 | 19 | 0.020072 |
| D011954---Receptors, Dopamine | 100 | 4 | 2 | 2 | 0.020081 |
| D018993---Myelin P0 Protein | 100 | 4 | 2 | 2 | 0.020081 |
| D008126---Lod Score | 390 | 9 | 4 | 5 | 0.020084 |
| D002258---Carbonyl Cyanide m-Chlorophenyl Hydrazone | 56 | 3 | 3 | 0 | 0.020193 |
| D006514---Hepatitis B Surface Antigens | 56 | 3 | 2 | 1 | 0.020193 |
| D011942---Receptors, Adrenergic, alpha | 56 | 3 | 1 | 2 | 0.020193 |
| D017366---Serotonin Receptor Agonists | 56 | 3 | 2 | 1 | 0.020193 |
| D055429---Growth Differentiation Factor 9 | 56 | 3 | 1 | 2 | 0.020193 |
| D007334---Insulin-Like Growth Factor I | 959 | 17 | 11 | 6 | 0.020345 |
| D012733---Sex Differentiation | 327 | 8 | 4 | 4 | 0.020415 |
| D008262---Macrophage Activation | 812 | 15 | 10 | 5 | 0.020761 |
| D051097---Glial Cell Line-Derived Neurotrophic Factor Receptors | 152 | 5 | 3 | 2 | 0.020864 |
| D051720---Upstream Stimulatory Factors | 152 | 5 | 4 | 1 | 0.020864 |
| D066257---Heparin-binding EGF-like Growth Factor | 152 | 5 | 2 | 3 | 0.020864 |
| D018628---Gene Dosage | 1039 | 18 | 9 | 9 | 0.021094 |
| D001710---Biotin | 208 | 6 | 3 | 3 | 0.021106 |
| D048370---MAP Kinase Kinase 2 | 208 | 6 | 2 | 4 | 0.021106 |
| D054339---Steroidogenic Factor 1 | 208 | 6 | 3 | 3 | 0.021106 |
| D005780---Gelatin | 57 | 3 | 2 | 1 | 0.021157 |
| D008560---Membrane Fluidity | 57 | 3 | 2 | 1 | 0.021157 |
| D016062---Porosity | 57 | 3 | 1 | 2 | 0.021157 |
| D011965---Receptors, Glucocorticoid | 529 | 11 | 6 | 5 | 0.021426 |
| D017395---Plasminogen Activator Inhibitor 1 | 268 | 7 | 4 | 3 | 0.021452 |
| D020319---Oligodeoxyribonucleotides, Antisense | 268 | 7 | 2 | 5 | 0.021452 |
| D006835---NA | 22 | 2 | 2 | 0 | 0.021471 |
| D002134---NA | 209 | 6 | 3 | 3 | 0.021551 |
| D018952---Antigens, CD34 | 395 | 9 | 3 | 6 | 0.021606 |
| D016283---Proto-Oncogene Proteins p21(ras) | 530 | 11 | 6 | 5 | 0.021691 |
| D002149---Energy Intake | 269 | 7 | 7 | 0 | 0.021841 |
| D019278---Matrix Metalloproteinase 3 | 210 | 6 | 4 | 2 | 0.022003 |
| D002842---Chromatids | 103 | 4 | 4 | 0 | 0.022116 |
| D051822---T Cell Transcription Factor 1 | 103 | 4 | 3 | 1 | 0.022116 |
| D051547---Heme Oxygenase-1 | 332 | 8 | 5 | 3 | 0.022121 |
| D043182---Carboxylesterase | 58 | 3 | 3 | 0 | 0.022146 |
| D051843---Synucleins | 58 | 3 | 3 | 0 | 0.022146 |
| D060589---Zyxin | 58 | 3 | 0 | 3 | 0.022146 |
| D020690---GTPase-Activating Proteins | 1045 | 18 | 9 | 9 | 0.022212 |
| D060833---Cellular Microenvironment | 270 | 7 | 2 | 5 | 0.022235 |
| D024681---Potassium Channels, Calcium-Activated | 155 | 5 | 4 | 1 | 0.022484 |
| D051740---Ikaros Transcription Factor | 155 | 5 | 3 | 2 | 0.022484 |
| D020449---Repetitive Sequences, Amino Acid | 271 | 7 | 3 | 4 | 0.022634 |
| D016161---Genes, Retinoblastoma | 104 | 4 | 3 | 1 | 0.022821 |
| D056151---Airway Remodeling | 104 | 4 | 1 | 3 | 0.022821 |
| D002217---Carbachol | 212 | 6 | 1 | 5 | 0.022925 |
| D011983---Receptors, Purinergic | 59 | 3 | 0 | 3 | 0.023159 |
| D059505---Telomere Homeostasis | 59 | 3 | 2 | 1 | 0.023159 |
| D004128---Dimethylnitrosamine | 23 | 2 | 1 | 1 | 0.023358 |
| D005228---Fatty Acids, Essential | 23 | 2 | 2 | 0 | 0.023358 |
| D014874---Water Pollutants, Chemical | 23 | 2 | 2 | 0 | 0.023358 |
| D015122---Mercaptopurine | 23 | 2 | 2 | 0 | 0.023358 |
| D050886---HSP20 Heat-Shock Proteins | 23 | 2 | 1 | 1 | 0.023358 |
| D049031---Sarcoglycans | 105 | 4 | 2 | 2 | 0.02354 |
| D057050---Receptor Tyrosine Kinase-like Orphan Receptors | 105 | 4 | 1 | 3 | 0.02354 |
| D009336---NA | 336 | 8 | 4 | 4 | 0.023558 |
| D051876---Sex-Determining Region Y Protein | 157 | 5 | 3 | 2 | 0.023608 |
| D052247---Nitric Oxide Synthase Type II | 752 | 14 | 9 | 5 | 0.023741 |
| D053764---Presenilin-1 | 337 | 8 | 6 | 2 | 0.023927 |
| D007527---Isoenzymes | 2023 | 30 | 16 | 14 | 0.024166 |
| D013925---Thromboplastin | 158 | 5 | 4 | 1 | 0.024183 |
| D047228---Angiotensin II Type 1 Receptor Blockers | 158 | 5 | 1 | 4 | 0.024183 |
| D015538---Nitrosation | 60 | 3 | 2 | 1 | 0.024198 |
| D042643---Vascular Endothelial Growth Factor D | 60 | 3 | 1 | 2 | 0.024198 |
| D051696---Activating Transcription Factors | 106 | 4 | 3 | 1 | 0.024273 |
| D014945---Wound Healing | 1133 | 19 | 13 | 6 | 0.024298 |
| D051766---Early Growth Response Protein 1 | 338 | 8 | 4 | 4 | 0.0243 |
| D059748---Proteolysis | 1451 | 23 | 14 | 9 | 0.02459 |
| D066253---Vascular Remodeling | 276 | 7 | 2 | 5 | 0.024704 |
| D010084---Oxidation-Reduction | 1372 | 22 | 11 | 11 | 0.024782 |
| D055551---HSP27 Heat-Shock Proteins | 216 | 6 | 5 | 1 | 0.024846 |
| D012685---Sepharose | 107 | 4 | 2 | 2 | 0.025019 |
| D007211---NA | 542 | 11 | 5 | 6 | 0.025068 |
| D004357---Drug Synergism | 613 | 12 | 7 | 5 | 0.025191 |
| D039921---Neuropilins | 61 | 3 | 1 | 2 | 0.025262 |
| D015694---Gene Products, rev | 24 | 2 | 1 | 1 | 0.025311 |
| D015752---Genes, env | 24 | 2 | 2 | 0 | 0.025311 |
| D018680---Cholinergic Antagonists | 24 | 2 | 2 | 0 | 0.025311 |
| D044067---Receptors, Lipoxin | 24 | 2 | 1 | 1 | 0.025311 |
| D000477---Alkylating Agents | 217 | 6 | 3 | 3 | 0.025342 |
| D002492---Central Nervous System Depressants | 217 | 6 | 5 | 1 | 0.025342 |
| D051705---Sp3 Transcription Factor | 160 | 5 | 3 | 2 | 0.025361 |
| D020778---Matrix Metalloproteinase 2 | 543 | 11 | 5 | 6 | 0.025366 |
| D018972---Insulin-Like Growth Factor Binding Protein 3 | 108 | 4 | 1 | 3 | 0.025779 |
| D008239---Lysine | 1219 | 20 | 12 | 8 | 0.025834 |
| D000975---Antioxidants | 762 | 14 | 7 | 7 | 0.026213 |
| D001616---beta-Galactosidase | 1541 | 24 | 15 | 9 | 0.026236 |
| D007632---Keratan Sulfate | 62 | 3 | 1 | 2 | 0.02635 |
| D050957---HSP110 Heat-Shock Proteins | 62 | 3 | 2 | 1 | 0.02635 |
| D051839---NFI Transcription Factors | 219 | 6 | 3 | 3 | 0.026355 |
| D021382---Protein Sorting Signals | 477 | 10 | 5 | 5 | 0.0264 |
| D001427---Bacterial Toxins | 344 | 8 | 5 | 3 | 0.026625 |
| D024141---Collagen Type IV | 344 | 8 | 4 | 4 | 0.026625 |
| D000111---Acetylcysteine | 410 | 9 | 4 | 5 | 0.026669 |
| D000942---Antigens, Bacterial | 345 | 8 | 6 | 2 | 0.027028 |
| D005976---Glutaral | 25 | 2 | 2 | 0 | 0.027329 |
| D006157---Guanosine Monophosphate | 25 | 2 | 1 | 1 | 0.027329 |
| D012021---Reflex, Abnormal | 25 | 2 | 2 | 0 | 0.027329 |
| D051239---Receptors, Vasoactive Intestinal Peptide, Type II | 25 | 2 | 1 | 1 | 0.027329 |
| D001683---Biological Clocks | 282 | 7 | 1 | 6 | 0.027355 |
| D004261---DNA Replication | 841 | 15 | 7 | 8 | 0.027375 |
| D016695---Glycosyltransferases | 221 | 6 | 3 | 3 | 0.027395 |
| D018816---Genes, cdc | 221 | 6 | 4 | 2 | 0.027395 |
| D058847---Inflammasomes | 412 | 9 | 7 | 2 | 0.027403 |
| D011980---Receptors, Progesterone | 346 | 8 | 6 | 2 | 0.027434 |
| D051880---Receptors, Notch | 1071 | 18 | 10 | 8 | 0.027592 |
| D010738---Type C Phospholipases | 481 | 10 | 4 | 6 | 0.02775 |
| D017781---Genes, Immediate-Early | 283 | 7 | 2 | 5 | 0.027815 |
| D016923---Cell Death | 1879 | 28 | 14 | 14 | 0.027832 |
| D015032---NA | 347 | 8 | 4 | 4 | 0.027845 |
| D021381---Protein Transport | 4161 | 54 | 34 | 20 | 0.027997 |
| D012260---Ribonucleases | 482 | 10 | 7 | 3 | 0.028095 |
| D017981---Receptors, Neurotransmitter | 111 | 4 | 3 | 1 | 0.028143 |
| D015321---Gene Rearrangement | 414 | 9 | 6 | 3 | 0.028151 |
| D017630---Connexins | 414 | 9 | 3 | 6 | 0.028151 |
| D006152---Cyclic GMP | 348 | 8 | 4 | 4 | 0.02826 |
| D018994---Myosin Light Chains | 284 | 7 | 3 | 4 | 0.02828 |
| D051785---Smad Proteins | 553 | 11 | 4 | 7 | 0.028488 |
| D012492---NA | 64 | 3 | 1 | 2 | 0.028602 |
| D013447---Sulfites | 64 | 3 | 1 | 2 | 0.028602 |
| D015243---Cholesterol, VLDL | 64 | 3 | 2 | 1 | 0.028602 |
| D005799---Genes, Dominant | 1076 | 18 | 11 | 7 | 0.028732 |
| D006358---Hot Temperature | 484 | 10 | 6 | 4 | 0.028795 |
| D024701---Myosin Type V | 112 | 4 | 2 | 2 | 0.028958 |
| D025721---WT1 Proteins | 224 | 6 | 3 | 3 | 0.029005 |
| D000903---Antibiotics, Antineoplastic | 485 | 10 | 6 | 4 | 0.029149 |
| D017353---Gene Deletion | 7919 | 93 | 61 | 32 | 0.029288 |
| D011944---Receptors, Androgen | 417 | 9 | 4 | 5 | 0.0293 |
| D009953---Ornithine-Oxo-Acid Transaminase | 26 | 2 | 1 | 1 | 0.029408 |
| D010410---Penile Erection | 26 | 2 | 1 | 1 | 0.029408 |
| D015245---Deoxyribonuclease BamHI | 26 | 2 | 1 | 1 | 0.029408 |
| D015636---Magnesium Chloride | 26 | 2 | 1 | 1 | 0.029408 |
| D018960---Hyaluronan Receptors | 486 | 10 | 6 | 4 | 0.029507 |
| D020797---Receptor, Platelet-Derived Growth Factor beta | 351 | 8 | 3 | 5 | 0.02953 |
| D009020---Morphine | 225 | 6 | 4 | 2 | 0.029556 |
| D004435---Eating | 487 | 10 | 5 | 5 | 0.029868 |
| D005819---Genetic Markers | 2832 | 39 | 27 | 12 | 0.029872 |
| D024042---Collagen Type I | 703 | 13 | 6 | 7 | 0.030288 |
| D050980---GATA Transcription Factors | 114 | 4 | 3 | 1 | 0.030631 |
| D053504---Matrix Metalloproteinases, Membrane-Associated | 114 | 4 | 0 | 4 | 0.030631 |
| D048749---Cytokinesis | 227 | 6 | 5 | 1 | 0.030677 |
| D058105---Polymerization | 227 | 6 | 4 | 2 | 0.030677 |
| D015342---DNA Probes | 1007 | 17 | 13 | 4 | 0.030803 |
| D002897---Chromosomes, Human, Pair 7 | 354 | 8 | 4 | 4 | 0.03084 |
| D014644---Genetic Variation | 1565 | 24 | 14 | 10 | 0.030841 |
| D037181---Lectins, C-Type | 632 | 12 | 6 | 6 | 0.030906 |
| D017302---Annexins | 66 | 3 | 2 | 1 | 0.030952 |
| D044402---Receptor, Serotonin, 5-HT2A | 66 | 3 | 2 | 1 | 0.030952 |
| D055537---Light Signal Transduction | 66 | 3 | 2 | 1 | 0.030952 |
| D000110---Acetylcholinesterase | 169 | 5 | 3 | 2 | 0.031115 |
| D058570---TOR Serine-Threonine Kinases | 1325 | 21 | 11 | 10 | 0.031461 |
| D009995---Osmosis | 115 | 4 | 2 | 2 | 0.031489 |
| D055415---Bone Morphogenetic Protein 4 | 562 | 11 | 5 | 6 | 0.031528 |
| D007768---Lactalbumin | 27 | 2 | 2 | 0 | 0.031549 |
| D010120---Oxytocics | 27 | 2 | 0 | 2 | 0.031549 |
| D012460---Sulfasalazine | 27 | 2 | 1 | 1 | 0.031549 |
| D043262---Ribonuclease P | 27 | 2 | 1 | 1 | 0.031549 |
| D051790---G-Box Binding Factors | 27 | 2 | 1 | 1 | 0.031549 |
| D013312---Stress, Physiological | 1246 | 20 | 13 | 7 | 0.031659 |
| D019718---Receptors, CXCR4 | 423 | 9 | 5 | 4 | 0.031696 |
| D004121---Dimethyl Sulfoxide | 170 | 5 | 3 | 2 | 0.031801 |
| D020126---Brefeldin A | 229 | 6 | 5 | 1 | 0.031826 |
| D051883---Receptor, Notch2 | 229 | 6 | 2 | 4 | 0.031826 |
| D019281---Dimerization | 1652 | 25 | 15 | 10 | 0.031852 |
| D010835---Phytohemagglutinins | 67 | 3 | 1 | 2 | 0.032165 |
| D013261---NA | 67 | 3 | 1 | 2 | 0.032165 |
| D017465---Receptors, Opioid, delta | 67 | 3 | 1 | 2 | 0.032165 |
| D051966---Phospholipase C gamma | 357 | 8 | 5 | 3 | 0.032189 |
| D014335---Tropomyosin | 116 | 4 | 2 | 2 | 0.03236 |
| D019948---Receptors, Interleukin-4 | 116 | 4 | 4 | 0 | 0.03236 |
| D044786---S-Phase Kinase-Associated Proteins | 230 | 6 | 6 | 0 | 0.03241 |
| D010101---Oxygen Consumption | 711 | 13 | 6 | 7 | 0.032766 |
| D053487---DEAD-box RNA Helicases | 566 | 11 | 5 | 6 | 0.032951 |
| D003300---Copper | 231 | 6 | 5 | 1 | 0.033002 |
| D007976---NA | 231 | 6 | 4 | 2 | 0.033002 |
| D016753---Interleukin-10 | 712 | 13 | 9 | 4 | 0.033086 |
| D045726---Metalloproteases | 172 | 5 | 4 | 1 | 0.033201 |
| D008715---Methionine | 427 | 9 | 7 | 2 | 0.033367 |
| D014746---NA | 232 | 6 | 4 | 2 | 0.033601 |
| D050436---Regulatory Elements, Transcriptional | 232 | 6 | 3 | 3 | 0.033601 |
| D051957---MSX1 Transcription Factor | 232 | 6 | 3 | 3 | 0.033601 |
| D048429---Cell Size | 1018 | 17 | 11 | 6 | 0.033684 |
| D009124---Muscle Proteins | 1827 | 27 | 12 | 15 | 0.033721 |
| D001384---NA | 28 | 2 | 1 | 1 | 0.03375 |
| D010122---Cystinyl Aminopeptidase | 28 | 2 | 1 | 1 | 0.03375 |
| D019081---O Antigens | 28 | 2 | 1 | 1 | 0.03375 |
| D019759---Carbon-Nitrogen Lyases | 28 | 2 | 1 | 1 | 0.03375 |
| D050808---ets-Domain Protein Elk-4 | 28 | 2 | 0 | 2 | 0.03375 |
| D050810---Octamer Transcription Factors | 28 | 2 | 2 | 0 | 0.03375 |
| D039961---Semaphorins | 295 | 7 | 3 | 4 | 0.033751 |
| D016474---Weight-Bearing | 173 | 5 | 4 | 1 | 0.033915 |
| D016588---Anticarcinogenic Agents | 173 | 5 | 4 | 1 | 0.033915 |
| D002470---Cell Survival | 3122 | 42 | 26 | 16 | 0.033955 |
| D010539---Permeability | 569 | 11 | 8 | 3 | 0.034047 |
| D004262---DNA Restriction Enzymes | 361 | 8 | 8 | 0 | 0.03405 |
| D009126---Muscle Relaxation | 118 | 4 | 1 | 3 | 0.034145 |
| D036183---Receptor, EphB2 | 118 | 4 | 2 | 2 | 0.034145 |
| D017868---Cyclic AMP-Dependent Protein Kinases | 1020 | 17 | 7 | 10 | 0.034229 |
| D023061---Gene Order | 570 | 11 | 8 | 3 | 0.034419 |
| D005801---Genes, Homeobox | 643 | 12 | 8 | 4 | 0.034616 |
| D053903---DNA Breaks, Double-Stranded | 430 | 9 | 7 | 2 | 0.03466 |
| D016084---Bronchoconstriction | 69 | 3 | 0 | 3 | 0.034663 |
| D020257---Ventricular Remodeling | 571 | 11 | 7 | 4 | 0.034793 |
| D053422---Receptor-Interacting Protein Serine-Threonine Kinases | 297 | 7 | 4 | 3 | 0.034817 |
| D007378---Interleukins | 500 | 10 | 6 | 4 | 0.034846 |
| D054730---Protein Interaction Domains and Motifs | 2086 | 30 | 17 | 13 | 0.035029 |
| D019704---Protein Disulfide-Isomerases | 119 | 4 | 4 | 0 | 0.035059 |
| D048051---p38 Mitogen-Activated Protein Kinases | 1261 | 20 | 11 | 9 | 0.035298 |
| D016203---CDC2 Protein Kinase | 298 | 7 | 4 | 3 | 0.035358 |
| D017127---Glycation End Products, Advanced | 175 | 5 | 2 | 3 | 0.035372 |
| D018931---Antineoplastic Agents, Hormonal | 175 | 5 | 2 | 3 | 0.035372 |
| D016222---Fibroblast Growth Factor 2 | 646 | 12 | 5 | 7 | 0.035681 |
| D059372---Nucleotide Motifs | 299 | 7 | 6 | 1 | 0.035905 |
| D003571---Cytochalasin B | 70 | 3 | 2 | 1 | 0.035949 |
| D009584---NA | 70 | 3 | 2 | 1 | 0.035949 |
| D026362---Serum Response Factor | 365 | 8 | 3 | 5 | 0.035983 |
| D000535---Aluminum | 29 | 2 | 1 | 1 | 0.036008 |
| D003703---Demecolcine | 29 | 2 | 2 | 0 | 0.036008 |
| D011255---Pregnancy Maintenance | 29 | 2 | 1 | 1 | 0.036008 |
| D012838---Silymarin | 29 | 2 | 2 | 0 | 0.036008 |
| D013652---Taste Threshold | 29 | 2 | 2 | 0 | 0.036008 |
| D015283---Citalopram | 29 | 2 | 2 | 0 | 0.036008 |
| D020156---Salicylic Acid | 29 | 2 | 2 | 0 | 0.036008 |
| D024341---Xanthophylls | 29 | 2 | 2 | 0 | 0.036008 |
| D051179---Plakins | 29 | 2 | 2 | 0 | 0.036008 |
| D000620---Aminoimidazole Carboxamide | 176 | 5 | 4 | 1 | 0.036115 |
| D007381---Intermediate Filament Proteins | 575 | 11 | 6 | 5 | 0.036319 |
| D019098---Telomerase | 300 | 7 | 4 | 3 | 0.036457 |
| D051844---alpha-Synuclein | 366 | 8 | 5 | 3 | 0.036478 |
| D020794---Receptor Protein-Tyrosine Kinases | 951 | 16 | 7 | 9 | 0.036699 |
| D006593---Hexokinase | 177 | 5 | 4 | 1 | 0.036868 |
| D015967---Gene Expression Regulation, Viral | 435 | 9 | 4 | 5 | 0.036892 |
| D014763---Viral Matrix Proteins | 121 | 4 | 1 | 3 | 0.036929 |
| D015127---9,10-Dimethyl-1,2-benzanthracene | 301 | 7 | 5 | 2 | 0.037015 |
| D000970---Antineoplastic Agents | 1268 | 20 | 12 | 8 | 0.037101 |
| D003564---Cytidine Deaminase | 238 | 6 | 4 | 2 | 0.037342 |
| D003609---Dactinomycin | 368 | 8 | 5 | 3 | 0.037481 |
| D014442---Monophenol Monooxygenase | 178 | 5 | 3 | 2 | 0.03763 |
| D053179---Caspase 7 | 178 | 5 | 5 | 0 | 0.03763 |
| D006147---NA | 122 | 4 | 2 | 2 | 0.037885 |
| D014314---Trisomy | 122 | 4 | 2 | 2 | 0.037885 |
| D019030---Receptors, Vitronectin | 122 | 4 | 1 | 3 | 0.037885 |
| D024061---Collagen Type III | 122 | 4 | 2 | 2 | 0.037885 |
| D051559---Hepatocyte Nuclear Factor 6 | 122 | 4 | 1 | 3 | 0.037885 |
| D009416---Nerve Regeneration | 508 | 10 | 5 | 5 | 0.038184 |
| D000578---NA | 30 | 2 | 1 | 1 | 0.038324 |
| D005583---Fourier Analysis | 30 | 2 | 1 | 1 | 0.038324 |
| D017378---Quinolinic Acid | 30 | 2 | 1 | 1 | 0.038324 |
| D009195---Peroxidase | 370 | 8 | 6 | 2 | 0.038502 |
| D020170---Caspase 1 | 370 | 8 | 7 | 1 | 0.038502 |
| D017430---Prostate-Specific Antigen | 72 | 3 | 2 | 1 | 0.038593 |
| D004347---Drug Interactions | 509 | 10 | 6 | 4 | 0.038617 |
| D015688---Oncogene Protein pp60(v-src) | 123 | 4 | 3 | 1 | 0.038856 |
| D017306---Annexin A2 | 123 | 4 | 3 | 1 | 0.038856 |
| D019808---Losartan | 123 | 4 | 1 | 3 | 0.038856 |
| D053331---Ectodysplasins | 123 | 4 | 2 | 2 | 0.038856 |
| D051741---Kruppel-Like Transcription Factors | 1437 | 22 | 11 | 11 | 0.038944 |
| D052250---Nitric Oxide Synthase Type III | 510 | 10 | 4 | 6 | 0.039052 |
| D051546---Cyclooxygenase 2 | 582 | 11 | 5 | 6 | 0.039102 |
| D000914---Antibodies, Viral | 180 | 5 | 5 | 0 | 0.039183 |
| D004229---Dithiothreitol | 180 | 5 | 4 | 1 | 0.039183 |
| D049231---Saposins | 180 | 5 | 4 | 1 | 0.039183 |
| D020013---Calcium Signaling | 1357 | 21 | 9 | 12 | 0.039297 |
| D017493---Leukocyte Common Antigens | 441 | 9 | 6 | 3 | 0.039698 |
| D052138---Genes, Neoplasm | 124 | 4 | 0 | 4 | 0.03984 |
| D008136---Longevity | 512 | 10 | 7 | 3 | 0.039934 |
| D008170---Lung Compliance | 73 | 3 | 1 | 2 | 0.039952 |
| D009129---Muscle Tonus | 73 | 3 | 0 | 3 | 0.039952 |
| D053607---Adrenomedullin | 73 | 3 | 2 | 1 | 0.039952 |
| D007145---Immunoglobulin kappa-Chains | 181 | 5 | 3 | 2 | 0.039974 |
| D017479---Receptors, Platelet-Derived Growth Factor | 242 | 6 | 2 | 4 | 0.039979 |
| D050880---p300-CBP Transcription Factors | 373 | 8 | 4 | 4 | 0.04007 |
| D006861---Hydrogen Peroxide | 962 | 16 | 10 | 6 | 0.040112 |
| D013629---Tamoxifen | 659 | 12 | 6 | 6 | 0.040569 |
| D019076---Transgenes | 1691 | 25 | 14 | 11 | 0.04061 |
| D053078---Membrane Potential, Mitochondrial | 443 | 9 | 6 | 3 | 0.040665 |
| D007168---IMP Dehydrogenase | 31 | 2 | 1 | 1 | 0.040695 |
| D053507---Matrix Metalloproteinase 11 | 31 | 2 | 1 | 1 | 0.040695 |
| D001610---Beta Particles | 4 | 1 | 1 | 0 | 0.040785 |
| D002936---Cinnarizine | 4 | 1 | 1 | 0 | 0.040785 |
| D005558---Arylformamidase | 4 | 1 | 1 | 0 | 0.040785 |
| D008935---Mitoguazone | 4 | 1 | 1 | 0 | 0.040785 |
| D009762---o-Aminoazotoluene | 4 | 1 | 1 | 0 | 0.040785 |
| D011754---Pyronine | 4 | 1 | 1 | 0 | 0.040785 |
| D013910---NA | 4 | 1 | 1 | 0 | 0.040785 |
| D014128---Chromomycin A3 | 4 | 1 | 1 | 0 | 0.040785 |
| D014334---NA | 4 | 1 | 1 | 0 | 0.040785 |
| D051964---Receptors, Mating Factor | 4 | 1 | 1 | 0 | 0.040785 |
| D062907---Oxaloacetic Acid | 4 | 1 | 1 | 0 | 0.040785 |
| D007371---Interferon-gamma | 1364 | 21 | 14 | 7 | 0.04119 |
| D005325---Fetal Organ Maturity | 74 | 3 | 0 | 3 | 0.041334 |
| D018040---Receptors, Neurokinin-1 | 74 | 3 | 1 | 2 | 0.041334 |
| D000199---Actins | 2116 | 30 | 20 | 10 | 0.041376 |
| D018992---Myelin-Associated Glycoprotein | 183 | 5 | 4 | 1 | 0.041586 |
| D011480---Protease Inhibitors | 445 | 9 | 7 | 2 | 0.041648 |
| D019444---Vaccines, DNA | 126 | 4 | 3 | 1 | 0.041852 |
| D017382---Reactive Oxygen Species | 1613 | 24 | 18 | 6 | 0.041888 |
| D014404---Tubulin | 1046 | 17 | 9 | 8 | 0.041918 |
| D002273---Carcinogens | 589 | 11 | 6 | 5 | 0.042032 |
| D019837---Ryanodine Receptor Calcium Release Channel | 245 | 6 | 2 | 4 | 0.042032 |
| D051796---Inhibitor of Differentiation Proteins | 1286 | 20 | 10 | 10 | 0.04205 |
| D053594---Myeloid Differentiation Factor 88 | 517 | 10 | 6 | 4 | 0.042199 |
| D005328---Fetal Viability | 184 | 5 | 2 | 3 | 0.042406 |
| D017467---Receptors, Erythropoietin | 184 | 5 | 3 | 2 | 0.042406 |
| D039621---Eukaryotic Initiation Factor-3 | 184 | 5 | 5 | 0 | 0.042406 |
| D051879---Twist-Related Protein 1 | 246 | 6 | 3 | 3 | 0.042731 |
| D006185---Habituation, Psychophysiologic | 75 | 3 | 2 | 1 | 0.042741 |
| D010528---Peristalsis | 75 | 3 | 1 | 2 | 0.042741 |
| D019793---Fluorescein | 75 | 3 | 1 | 2 | 0.042741 |
| D037021---Thyroid Hormone Receptors alpha | 75 | 3 | 1 | 2 | 0.042741 |
| D038982---Integrin alpha2beta1 | 75 | 3 | 3 | 0 | 0.042741 |
| D060746---Endoplasmic Reticulum-Associated Degradation | 75 | 3 | 2 | 1 | 0.042741 |
| D001053---Apolipoproteins | 127 | 4 | 2 | 2 | 0.042879 |
| D027701---Organic Cation Transport Proteins | 127 | 4 | 3 | 1 | 0.042879 |
| D044385---GTP-Binding Protein alpha Subunits | 127 | 4 | 2 | 2 | 0.042879 |
| D001498---3-Pyridinecarboxylic acid, 1,4-dihydro-2,6-dimethyl-5-nitro-4-(2-(trifluoromethyl)phenyl)-, Methyl ester | 32 | 2 | 1 | 1 | 0.043119 |
| D010745---NA | 32 | 2 | 1 | 1 | 0.043119 |
| D011341---Probucol | 32 | 2 | 1 | 1 | 0.043119 |
| D038202---alpha-Crystallin A Chain | 32 | 2 | 1 | 1 | 0.043119 |
| D043283---Chromosome Fragile Sites | 32 | 2 | 1 | 1 | 0.043119 |
| D053305---Apolipoprotein C-III | 32 | 2 | 1 | 1 | 0.043119 |
| D009707---Nucleosomes | 379 | 8 | 6 | 2 | 0.043332 |
| D006820---Hyaluronic Acid | 247 | 6 | 3 | 3 | 0.043438 |
| D018094---Receptors, Metabotropic Glutamate | 247 | 6 | 2 | 4 | 0.043438 |
| D007106---Immune Sera | 449 | 9 | 7 | 2 | 0.043662 |
| D019255---NADPH Oxidases | 593 | 11 | 7 | 4 | 0.043773 |
| D042421---Histone Code | 128 | 4 | 0 | 4 | 0.04392 |
| D002166---Camptothecin | 186 | 5 | 2 | 3 | 0.044076 |
| D051701---Activating Transcription Factor 4 | 248 | 6 | 5 | 1 | 0.044151 |
| D001685---Biological Factors | 76 | 3 | 1 | 2 | 0.044171 |
| D039841---Leukocyte L1 Antigen Complex | 76 | 3 | 3 | 0 | 0.044171 |
| D051402---Aquaporin 5 | 76 | 3 | 2 | 1 | 0.044171 |
| D051573---Proto-Oncogene Proteins c-pim-1 | 76 | 3 | 2 | 1 | 0.044171 |
| D005075---Biological Evolution | 975 | 16 | 10 | 6 | 0.044437 |
| D015971---Gene Expression Regulation, Enzymologic | 2737 | 37 | 18 | 19 | 0.044511 |
| D011498---Protein Precursors | 821 | 14 | 7 | 7 | 0.044854 |
| D016708---Synaptophysin | 249 | 6 | 3 | 3 | 0.044873 |
| D000682---Amyloid | 187 | 5 | 3 | 2 | 0.044925 |
| D012079---Renal Circulation | 129 | 4 | 1 | 3 | 0.044976 |
| D008156---Luciferases | 1709 | 25 | 13 | 12 | 0.045216 |
| D010749---Phosphoprotein Phosphatases | 900 | 15 | 7 | 8 | 0.045517 |
| D016271---Proto-Oncogene Proteins c-myc | 1057 | 17 | 9 | 8 | 0.045524 |
| D005073---Evoked Potentials, Somatosensory | 33 | 2 | 2 | 0 | 0.045597 |
| D007854---NA | 33 | 2 | 2 | 0 | 0.045597 |
| D008689---NA | 33 | 2 | 2 | 0 | 0.045597 |
| D010715---Phosphatidylglycerols | 33 | 2 | 0 | 2 | 0.045597 |
| D013453---Sulfonylurea Compounds | 33 | 2 | 0 | 2 | 0.045597 |
| D019830---Adenosine-5'-(N-ethylcarboxamide) | 33 | 2 | 0 | 2 | 0.045597 |
| D044405---Receptor, Serotonin, 5-HT2C | 33 | 2 | 2 | 0 | 0.045597 |
| D054522---Group VI Phospholipases A2 | 33 | 2 | 1 | 1 | 0.045597 |
| D059004---Topoisomerase I Inhibitors | 33 | 2 | 0 | 2 | 0.045597 |
| D005072---Evoked Potentials, Auditory | 77 | 3 | 3 | 0 | 0.045625 |
| D001370---Axonal Transport | 188 | 5 | 5 | 0 | 0.045785 |
| D018167---Receptors, Calcitriol | 188 | 5 | 3 | 2 | 0.045785 |
| D020741---rho GTP-Binding Proteins | 824 | 14 | 7 | 7 | 0.046003 |
| D016159---Tumor Suppressor Protein p53 | 1796 | 26 | 16 | 10 | 0.046033 |
| D039564---Integrin alphaV | 130 | 4 | 2 | 2 | 0.046045 |
| D013438---Sulfhydryl Compounds | 189 | 5 | 3 | 2 | 0.046654 |
| D000950---Antigens, Ly | 455 | 9 | 7 | 2 | 0.046805 |
| D003602---Cytotoxicity, Immunologic | 527 | 10 | 5 | 5 | 0.04699 |
| D005978---Glutathione | 527 | 10 | 7 | 3 | 0.04699 |
| D058207---Sumoylation | 252 | 6 | 3 | 3 | 0.04708 |
| D000478---Alkylation | 78 | 3 | 2 | 1 | 0.047103 |
| D011073---NA | 78 | 3 | 3 | 0 | 0.047103 |
| D018435---ATP Binding Cassette Transporter, Subfamily B | 131 | 4 | 2 | 2 | 0.047129 |
| D019831---Sodium-Calcium Exchanger | 131 | 4 | 2 | 2 | 0.047129 |
| D013917---Thrombin | 386 | 8 | 2 | 6 | 0.047355 |
| D053614---Janus Kinase 2 | 386 | 8 | 6 | 2 | 0.047355 |
| D010802---Phylogeny | 10331 | 115 | 70 | 45 | 0.047437 |
| D015335---Molecular Probes | 190 | 5 | 5 | 0 | 0.047533 |
| D011312---Pressure | 253 | 6 | 3 | 3 | 0.047831 |
| D016718---Arachidonic Acid | 253 | 6 | 3 | 3 | 0.047831 |
| D018950---Lipopolysaccharide Receptors | 253 | 6 | 5 | 1 | 0.047831 |
| D051745---Protein Kinase C-delta | 319 | 7 | 1 | 6 | 0.048035 |
| D012604---Scorpion Venoms | 34 | 2 | 2 | 0 | 0.048125 |
| D017293---Protein S | 34 | 2 | 0 | 2 | 0.048125 |
| D051269---Sodium-Glucose Transporter 1 | 34 | 2 | 2 | 0 | 0.048125 |
| D051929---CD146 Antigen | 34 | 2 | 1 | 1 | 0.048125 |
| D009573---NA | 132 | 4 | 2 | 2 | 0.048227 |
| D010958---Plasminogen | 132 | 4 | 2 | 2 | 0.048227 |
| D051266---Nuclear Respiratory Factor 1 | 132 | 4 | 2 | 2 | 0.048227 |
| D000444---Aldehyde Dehydrogenase | 191 | 5 | 5 | 0 | 0.048421 |
| D020800---Receptor, Nerve Growth Factor | 191 | 5 | 1 | 4 | 0.048421 |
| D051519---Fibroblast Growth Factor 4 | 191 | 5 | 3 | 2 | 0.048421 |
| D017874---Immediate-Early Proteins | 678 | 12 | 5 | 7 | 0.048535 |
| D018008---NA | 254 | 6 | 2 | 4 | 0.048588 |
| D012337---RNA, Ribosomal, 18S | 79 | 3 | 3 | 0 | 0.048604 |
| D046188---Protein Modification, Translational | 79 | 3 | 3 | 0 | 0.048604 |
| D048748---MAP Kinase Kinase Kinase 3 | 79 | 3 | 1 | 2 | 0.048604 |
| D004676---Myelin Basic Protein | 320 | 7 | 4 | 3 | 0.048702 |
| D000943---Antigens, Differentiation | 831 | 14 | 8 | 6 | 0.048764 |
| D010455---Peptides | 2757 | 37 | 21 | 16 | 0.048954 |
| D011958---NA | 531 | 10 | 5 | 5 | 0.049006 |
| D019943---Amino Acid Substitution | 1640 | 24 | 15 | 9 | 0.04928 |
| D005746---Gastric Emptying | 192 | 5 | 3 | 2 | 0.04932 |
| D015259---Dopamine Agents | 133 | 4 | 2 | 2 | 0.049339 |
| D024501---Nonmuscle Myosin Type IIB | 133 | 4 | 2 | 2 | 0.049339 |
| D012045---Regulatory Sequences, Nucleic Acid | 1149 | 18 | 9 | 9 | 0.049696 |
| D020121---5' Untranslated Regions | 606 | 11 | 6 | 5 | 0.049775 |
| D002633---Chemotaxis | 681 | 12 | 8 | 4 | 0.049885 |
